# Non-enzymatic pyridine ring formation in the biosynthesis of the rubrolone tropolone alkaloids

**DOI:** 10.1038/ncomms13083

**Published:** 2016-10-07

**Authors:** Yijun Yan, Jing Yang, Zhiyin Yu, Mingming Yu, Ya-Tuan Ma, Li Wang, Can Su, Jianying Luo, Geoffrey P. Horsman, Sheng-Xiong Huang

**Affiliations:** 1 State Key Laboratory of Phytochemistry and Plant Resources in West China, Kunming Institute of Botany, Chinese Academy of Sciences, Kunming 650201, China; 2 Department of Chemistry & Biochemistry, Wilfrid Laurier University, Waterloo, Ontario, Canada N2L 3C5

## Abstract

The pyridine ring is a potent pharmacophore in alkaloid natural products. Nonetheless, its biosynthetic pathways are poorly understood. Rubrolones A and B are tropolone alkaloid natural products possessing a unique tetra-substituted pyridine moiety. Here, we report the gene cluster and propose a biosynthetic pathway for rubrolones, identifying a key intermediate that accumulates upon inactivation of sugar biosynthetic genes. Critically, this intermediate was converted to the aglycones of rubrolones by non-enzymatic condensation and cyclization with either ammonia or anthranilic acid to generate the respective pyridine rings. We propose that this non-enzymatic reaction occurs via hydrolysis of the key intermediate, which possesses a 1,5-dione moiety as an amine acceptor capable of cyclization. This study suggests that 1,5-dione moieties may represent a general strategy for pyridine ring biosynthesis, and more broadly highlights the utility of non-enzymatic diversification for exploring and expanding natural product chemical space.

## Abstract (web summary)

The biosynthesis of pyridine rings is still poorly understood. Here the authors propose a biosynthetic pathway for pyridine-containing rubrolones, which is characterized by a non-enzymatic condensation and cyclization of the pyridine moiety.

Rubrolone A (**1**) was first reported in 1978 as a red pigment produced by *Streptomyces enchinoruber*^1^, and subsequent structure elucidation revealed a nearly coplanar and highly conjugated aglycone consisting of a tropolone ring, a five-membered cyclopentanone, and a pyridine ring^2^ (Fig. 1). A unique carbon–carbon bond links C-2 of the deoxysugar to the tropolone ring of the aglycone. This highly oxygenated hybrid structure with distinct ring assembly has inspired chemists and microbiologists, but only two total synthetic routes to the rubrolone aglycone have been devised^3^,^4^ and the biosynthetic pathway has not been elucidated.

Although rubrolone A has shown no significant biological activity in cytotoxicity and antimicrobial assays^1^, we recently isolated rubrolone B (**2**) (Fig. 1) as a new potentially cardioprotective rubrolone from the endophytic strain *Streptomyces* sp. KIB-H033 (*S.* sp. KIB-H033)^5^. Importantly, feeding experiments with [^13^C]-acetate demonstrated aglycone assembly via type II PKS chemistry followed by complex oxidative rearrangements to afford the tropolone, which differs from the pinacol rearrangement-based ring expansion during fungal stipitatic acid tropolone biosynthesis^6^. Moreover, the additional benzoic acid moiety of rubrolone B (**2**) compared with rubrolone A (**1**) provides an example of a unique cationic inner salt moiety, the presence of which raises questions about biosynthetic pathway logic and timing; for example, is rubrolone A the biosynthetic precursor to rubrolone B, or vice versa?

The rubrolones are members of the pyridine alkaloid family of natural products (Fig. 1) found widely in nature and possessing antimalarial, anticancer and antibacterial activities^7^,^8^,^9^,^10^,^11^. The biologically significant pyridyl moiety is assembled via diverse biosynthetic pathways that primarily originate from amino acid precursors. For example, the pyridine rings of virginiamycin S (ref. 12) and nikkomycin^13^ derive from L-lysine, while aspartate generates the pyridyl moiety of nicotine^14^ and pyridomycin^15^ and L-tryptophan is the precursor for the pyridyl moiety of thiocoraline^16^ (Fig. 1). Only in rare cases are pyridine alkaloids derived from other sources; for example, one of the collismycin pyridine rings is derived from L-cysteine and acetate (Fig. 1)^17^. However, because most of these insights have been achieved from feeding isotope-labelled precursors, the detailed mechanisms of alkaloid pyridine ring formation are poorly understood.

To understand the mechanism of rubrolone biosynthesis in *S*. sp. KIB-H033, and pyridyl construction in particular, we herein report the biosynthetic gene cluster together with heterologous expression experiments that enable us to propose a biosynthetic pathway. Critically, inactivation of rubrolone sugar biosynthetic genes led to the accumulation of a key intermediate that was shown to react non-enzymatically with either ammonia or anthranilic acid to afford the respective aglycones of rubrolones A (**1**) and B (**2**). This study provides important new insight into the non-enzymatic and non-amino acid origins of the pyridyl moiety in alkaloid natural products.

## Results

### Identification of the rubrolone biosynthetic gene cluster

**Figure 2:**
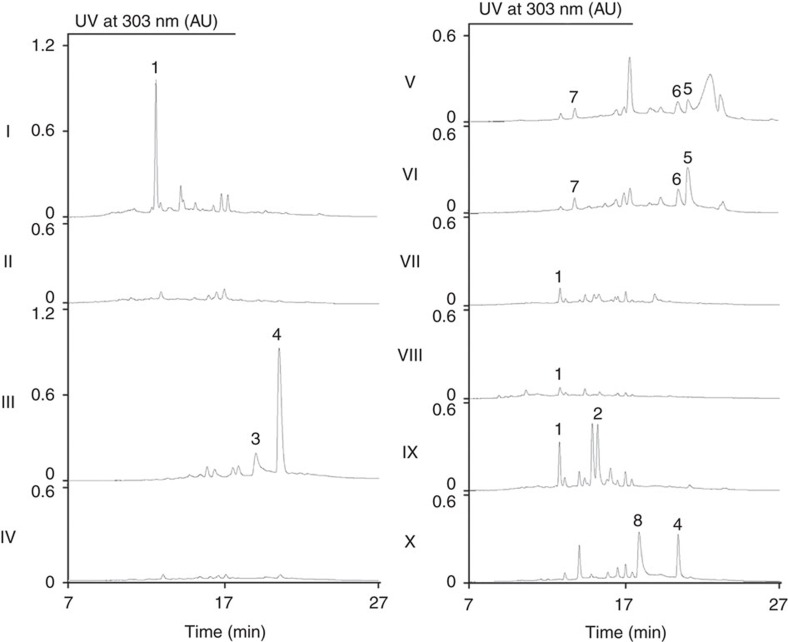
HPLC profiles of the fermentation extracts of engineered heterologous expression strains. I: *S. albus* 9B10; II: *S. albus* pJTU2554; III: *S. albus* 9B10-Δ*S1*; IV: *S. albus* 9B10-Δ*E9*; V: *S. albus* 9B10-Δ*B*; VI: *S. albus* 9B10-Δ*C*; VII: *S. albus* 9B10-Δ*E9* feeding with **3**; VIII: *S. albus* 9B10-Δ*E9* feeding with **4**; IX: *S. albus* 9B10 feeding with anthranilic acid; and X: *S. albus* 9B10-Δ*S1* feeding with anthranilic acid.

Our strategy for identifying the rubrolone (*rub*) biosynthetic gene cluster was based on our hypotheses that (i) the aglycone of rubrolones A and B is of polyketide synthase origin^5^, and (ii) a sugar 4,6-dehydratase installs the C-6 methyl group of the deoxysugar. The dehydratase-encoding gene has been used as a probe to successfully identify the gene cluster of the enediyne antibiotic C-1027 (ref. 18). To search for these two gene loci, the genomic deoxyribonucleic acid (DNA) of *S.* sp. KIB-H033 was subjected to Illumina sequencing. Using KSα and sugar 4,6-dehydratase genes as sequence queries to scan the assembled scaffolds, we identified a 20-kb region of the genome possessing both genes. To validate the involvement of this DNA region in rubrolone biosynthesis, a genomic library of *S.* sp. KIB-H033 was constructed based on cosmid pJTU2554 (ref. 19), from which the cosmid p9B10 containing this DNA region was identified through library screening. Integration of p9B10 into the chromosome of *Streptomyces albus* J1074 (ref. 20) by conjugation generated strain *S. albus* 9B10, from which heterologous expression of the putative rubrolone gene cluster could be monitored by a colour change on an agar plate; the recombinant strain produced a ruby red pigment, whereas control strain containing the relevant empty vector pJTU2554 did not (Supplementary Fig. 1). To confirm the identity of this red metabolite, liquid culture extracts of recombinant strain *S. albus* 9B10 and appropriate control were analysed by high-performance liquid chromatography (HPLC). The putative *rub*-containing clone produced a peak with retention time identical to that of rubrolone A (Fig. 2, trace I), and this peak was absent from the *S. albus* control strain containing the empty vector (Fig. 2, trace II). The presence of rubrolone A (**1**) was further confirmed by high-resolution electrospray ionization mass spectrometry (HRESIMS) and ^1^H NMR spectroscopy. Overall, these results demonstrated that cosmid 9B10 possesses the requisite gene cluster encoding the biosynthesis of rubrolone A.

### Determining the *rub* gene cluster boundaries

**Figure 3:**
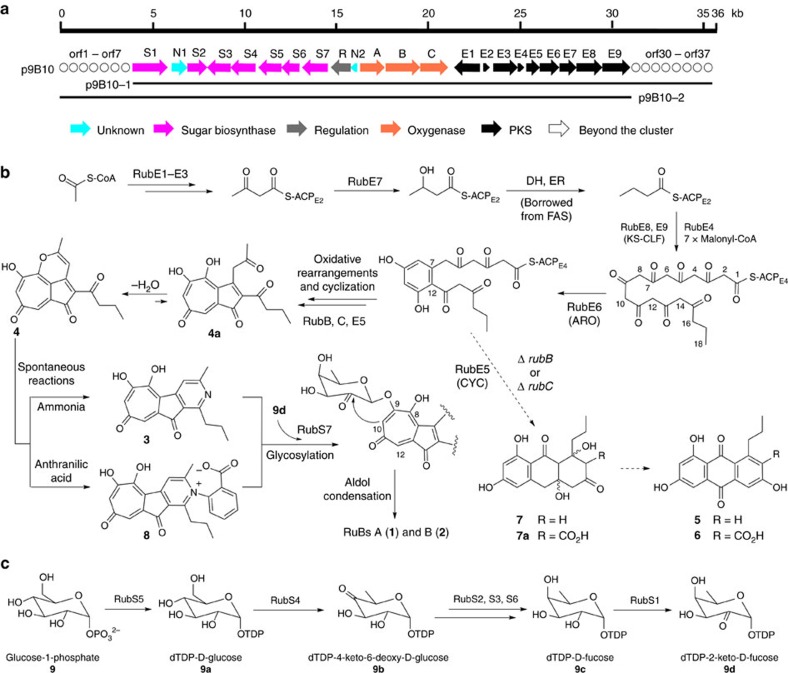
Biosynthetic gene cluster and proposed biosynthetic pathway of rubrolones. (**a**) Organization of the *rub* biosynthetic gene cluster, with functional assignment of genes including PKS (black), oxygenases (orange), deoxysugar synthases (pink), regulation (green), unknown (cyan) and genes outside the cluster (white). (**b**) Proposed biosynthetic pathway for PKS and post-PKS modifications, with dashed arrows indicating the pathway generating the shunt metabolites R1128A (**5**), **6** and **7**. (**c**) Proposed biosynthetic pathway for the deoxysugar dTDP-2-keto-D-fucose.

The sequence of the 37 open reading frames identified in cosmid 9B10 (Fig. 3a) has been deposited in Genbank with accession number KX218108. To determine the *rub* gene cluster boundaries, we first constructed cosmid p9B10-1 (from genes *rubS1* to *orf37*, Fig. 3a) using λ red-mediated polymerase chain reaction (PCR) targeting mutagenesis^21^, and this strain still produced rubrolone A (Supplementary Fig. 2, trace I). In contrast, inactivation of the oxidoreductase-encoding *rubS1* gene abolished production of **1** in the new recombinant strain *S. albus* 9B10-Δ*S1*, but resulted in the accumulation of two new compounds **3** and **4** (Fig. 2, trace III). Therefore, the left boundary of this gene cluster was determined to be *rubS1*. To locate the right boundary of the gene cluster, cosmid p9B10-2 (genes from *orf1* to *rubE9,* Fig. 3a) was constructed and was then conjugated into *S. albus* generating the engineered strain *S. albus* 9B10-2, which did not affect the production of **1** (Supplementary Fig. 2, trace II). The gene at the right boundary of cosmid 9B10-2 is the putative ketosynthase (KS) chain length factor (CLF) encoding gene *rubE9*, in-frame deletion of which in strain *S. albus* 9B10-Δ*E9* abolished production of **1** (Fig. 2, trace IV), confirming type II PKS involvement in rubrolone biosynthesis. Having established *rubS1* and *rubE9* as the boundaries of the ∼22.5 kb *rub* gene cluster (Fig. 3a), we then analysed all 22 ORFs using a basic local alignment search tool to assign putative functions (Supplementary Table 1). In summary, the *rub* biosynthetic genes encode: (i) PKS and associated enzymes (RubE1–E9), (ii) sugar biosynthesis enzymes (RubS1–S7), (iii) oxygenases (RubA–C) and the regulatory protein RubR and (iv) two unknown proteins (RubN1 and RubN2).

**Figure 4:**
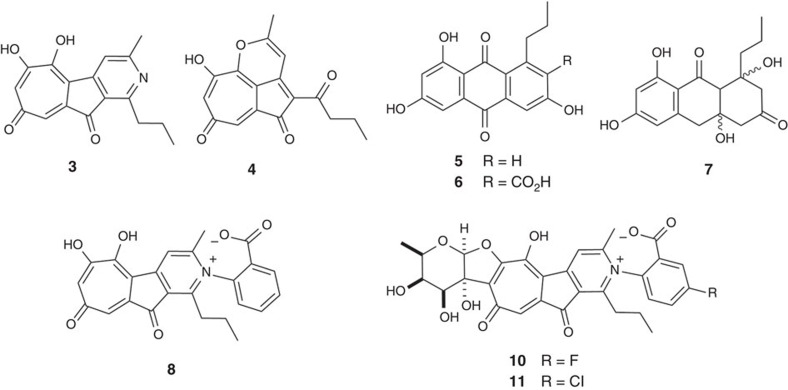
Chemical structures of rubrolone analogues and related metabolites produced by mutants. Compounds **3** and **4** were isolated from mutant *S. albus* 9B10-ΔS1; **5**–**7** were isolated from mutant *S. albus* 9B10-ΔB; **8** was obtained from *S. albus* 9B10-Δ*S1* feeding with anthranilic acid; and compounds **9** and **10** were obtained from *S. albus* 9B10 feeding with 2-amino-5-fluorobenzoic acid and 2-amino-5-chlorobenzoic acid, respectively.

A scale-up fermentation of *S. albus* 9B10-Δ*S1* was carried out to provide sufficient amounts of compounds **3** and **4** for structural characterization. The HRESIMS peak at *m/z* 298.1077 [M+H]^+^ for C_17_H_16_NO_4_ (calcd. 298.1074), together with the nuclear magnetic resonance (NMR) data comparison with literature values confirmed that **3** was the rubrolone aglycone^3^,^4^. For compound **4**, the observed exact mass at *m/z* 297.0780 [M−H]^−^ for C_17_H_13_O_5_ (calcd. 297.0769) indicated that the nitrogen atom in **3** was replaced by oxygen in **4**. Full NMR analysis including ^1^H and ^13^C NMR as well as two-dimensional (2D) NMR experiments (Supplementary Table 2; Supplementary Fig. 3), ^1^H−^1^H COSY, HSQC, and HMBC, identified the structure of **4** as shown in Fig. 4.

### Enzymes in the biosynthesis of the rubrolone scaffold

Our previous ^13^C-acetate feeding experiments demonstrated that the aglycone originates from type II PKS chemistry followed by complex oxidative rearrangements^5^. Bioinformatic analysis yields the expected minimal type II PKS encoded by *rubE8* (KSα), *rubE9* (CLF/KS*β*) and *rubE4* (acyl carrier protein, ACP), which share high sequence similarity with the type II PKS involved in R1128 biosynthesis (ZhuB, 79% identity; ZhuA, 71% identity; and ZhuN, 68% identity)^22^. Additionally, *rubE1*, *rubE2* and *rubE3* encode β-ketoacyl/ACP synthase III (KSIII), ACP and an acyltransferase, respectively, which share high sequence similarity with a set of starter unit butyryl-ACP generating enzymes (ZhuH, 61% identity; ZhuG, 53% identity; and ZhuC, 56% identity) in R1128 biosynthesis^23^,^24^. Two putative polyketide cyclases, RubE5 and RubE6, are also found within the *rub* gene cluster and share high sequence similarity with the cyclase/dehydratase protein ZhuJ (69% identity) and cyclase ZhuI (79% identity), respectively^25^. Cyclase RubE6 may catalyse the C7/C12 cyclization and aromatization of the linear polyketide intermediate as does the ZhuI ARO/CYC in R1128 biosynthesis (Fig. 3b), with subsequent cyclization catalysed by the ZhuJ cyclase homologue RubE5. Significantly, the R1128 and rubrolone biosynthetic gene clusters share the same suite of PKS genes (Supplementary Fig. 4, comparison of the two gene clusters), which is consistent with common structural features of a propyl side chain and the same PKS chain length in both R1128 and rubrolones (Fig. 3b). Interestingly, the putative reductase RubE7 is not found in the R1128 biosynthetic gene cluster, and may function as the missing ketoreductase (KR) responsible for generating the butyryl starter unit (Fig. 3b). Inactivation of *rubE7* slightly reduced the production of **1** to ∼60% of production in *S. albus* 9B10 (Supplementary Fig. 2, trace III), consistent with previous assumption upon partial complementation by the KR from fatty acid biosynthesis^22^.

Overall, bioinformatic analysis of the *rub* gene cluster has identified PKS genes with high sequence similarity to genes responsible for the biosynthesis of R1128 (Supplementary Table 1)^22^. This supports our previous proposal of KSIII-catalysed formation of the butyryl-ACP starter unit for type II PKS-catalysed extension with seven malonyl-CoA additions to generate the highly reactive poly-β-ketone backbone (Fig. 3b).

### Enzymes for oxidative formation of the tropolone scaffold

Although clearly of polyketide origin, the rubrolone carbon skeleton possesses a labelling pattern consistent with a series of complex oxidative rearrangements^5^. There are three putative oxygenases in the robulone gene cluster: RubA belongs to the bacterial luciferase-like monooxygenase superfamily^26^; RubB possesses 55% identity to ZhuM, a cytochrome P450 oxygenase catalysing anthraquinone formation in R1128 biosynthesis^22^; RubC is similar to naphthocyclinone hydroxylases like ActVA-orf5 (59% identity) that catalyse ring hydroxylation in the actinorhodin biosynthetic pathway^27^.

To determine if any of these three oxygenases may be involved in tropolone ring formation, we constructed the respective in-frame gene deletion mutants *S. albus* 9B10-Δ*A*, *S. albus* 9B10-Δ*B* and *S. albus* 9B10-Δ*C* for metabolite analysis by HPLC. Although inactivation of *rubA* did not affect rubrolone production (Supplementary Fig. 2, trace IV), both *S. albus* 9B10-Δ*B* and *S. albus* 9B10-Δ*C* shared a similar metabolite profile that included abolished production of **1** together with three new peaks **5**–**7** (Fig. 2, traces V and VI). Large-scale fermentation of *S. albus* 9B10-Δ*B* enabled the purification of sufficient quantities of **5**–**7** for structure elucidation by mass spectrometry (MS) and NMR. The known structure of R1128A (**5**) was determined by comparison to the ^1^H and ^13^C NMR data in the literature^28^. The structures of the new compounds **6** and **7** were elucidated by a combination of HRESIMS and one-dimensional (1D) and 2D NMR spectroscopic analyses (Supplementary Table 3; Supplementary Fig. 3).

Because deletion of either *rubB* or *rubC* completely abolished production but resulted in the accumulation of R1128A (**5**) and the related compounds **6** and **7** (Fig. 3b), both RubB and RubC are required for tropolone ring formation, and without both of these enzymes present RubE5 and/or RubE6 can catalyse cyclization and dehydration reactions analogous to those catalysed by their homologues ZhuJ and ZhuI to furnish R1128A. In addition, RubB, RubC and/or an oxidase in the host strain may catalyse oxidation and possibly also the dehydration reactions that transform **7** to R1128A (**5**) (Fig. 3b); a similar bifunctional oxygenase/dehydratase has been found in jadomycin biosynthesis^29^. The isolation of **5**–**7** from mutant strains is unexpected but consistent with the presence of homologues of all R1128 biosynthetic genes in the *rub* gene cluster (Supplementary Fig. 4). Taken together, the data suggest that RubB and RubC together may catalyse the complex oxidative rearrangements leading to tropolone ring formation, and that these oxygenases may work on the ACP-bound intermediate before the second cyclization to direct biosynthetic divergence between rubrolones and R1128 (Fig. 3b).

### Sugar gene mutants accumulate 3 and 4 as precursors to 1

Glycosylation can vastly expand natural product chemical space due to the structural diversity of sugars and their varying modes of aglycone attachment^30^,^31^. Rubrolones possess a rare D-fucose deoxysugar with a unique 1,2-linkage to the seven-membered tropolone ring that implies a dTDP-2-keto-D-fucose precursor. Seven genes (*rubS1*–*rubS7*) in the *rub* gene cluster encode enzymes putatively assigned to the biosynthesis of the deoxysugar moiety and its attachment to the aglycone. We propose that the glucose-1-phosphate thymidylyltransferase (RubS5)^32^ and dTDP-glucose 4,6-dehydratase (RubS4)^33^ sequentially transform glucose-1-phosphate (**9**) to dTDP-4-keto-6-deoxy-D-glucose (**9b**) via dTDP-D-glucose (**9a**), followed by RubS3-catalysed ketoreduction to dTDP-D-fucose (**9c**) (Fig. 3c)^34^. RubS2 (ref. 35) and RubS6 (ref. 36) are putative NAD-dependent epimerases that may also catalyse this ketoreduction. RubS1 possesses an FAD binding motif and 52% identity to the AknOx oxidoreductase catalysing the final two steps of aclacinomycin biosynthesis, the oxidation of the terminal sugar rhodinose to L-aculose^37^. We therefore propose that RubS1 transforms dTDP-D-fucose to dTDP-2-keto-D-fucose (**9d**) (Fig. 3c) before installation on the rubrolone scaffold by the glycosyltransferase RubS7 (Fig. 3b). Finally, we propose an intramolecular aldol reaction between the 2-keto group of the deoxysugar and C-10 of the aglycone to yield the rubrolones (Fig. 3b).

To probe the biosynthetic functions of RubS1–S7, we inactivated the *rubS2*, *rubS3* and *rubS7* genes to afford mutant strains *S. albus* 9B10-Δ*S2*, *S. albus* 9B10-Δ*S3* and *S. albus* 9B10-Δ*S7*, respectively. All three mutations completely abolished rubrolone production, but accumulated **3** and **4** and shared the same metabolite profile as that of *S. albus* 9B10-Δ*S1* (Supplementary Fig. 2, traces V–VII). Both **3** and **4** possess the characteristic tropolone ring, and **3** is the rubrolone aglycone (Fig. 4).

To determine if these compounds are biosynthetic intermediates or shunt products, **3** and **4** were fed to a mutant with rubrolone biosynthesis blocked at an early stage. RubE8 (KSα) and RubE9 (CLF) form a heterodimer catalysing Claisen-like condensation of malonyl-ACP extender units. The *rubE9*-inactivated mutant *S. albus* 9B10-Δ*E9* generated a clean background with no rubrolone-related metabolites detected by HPLC (Fig. 2, trace IV), and therefore was used as the host to carry out feeding experiments with **3** and **4**. HPLC chromatograms revealed that both compounds were completely converted to **1** (Fig. 2, traces VII and VIII), suggesting that both **3** and **4** are biosynthetically competent intermediates en route to rubrolone A (**1**).

To investigate the timing and specificity of the putative glycosyltransferase RubS7, we sought to present this enzyme with large quantities of **4**, which is the precursor of the pyridyl-containing aglycone. This was achieved by decreasing the amount of nitrogen source in the culture media, which dramatically decreased the production of **1** and accumulated large amounts of **4** in the heterologous expression strain *S. albus* 9B10 (Supplementary Fig. 2, trace VIII). This suggests that the downstream glycosyltransferase RubS7 is specific for the pyridyl-containing aglycone, and therefore pyridyl moiety formation occurs before glycosylation.

Our collective interrogation of the sugar biosynthetic genes suggests a biosynthetic pathway from glucose-1-phosphate to dTDP-2-keto-D-fucose, which is installed on the fully maturated pyridyl-containing rubrolone aglycone by the glycosyltransferase RubS7 (Fig. 3c). An unprecedented carbon–carbon bond between C-2 of the sugar and C-10 of the aglycone is then formed by an unknown mechanism. Significantly, the compounds **3** and **4** that accumulate upon sugar biosynthetic gene inactivation are biosynthetic intermediates en route to rubrolones.

### Anthranilic acid is a biosynthetic precursor to product 2

**Figure 5:**
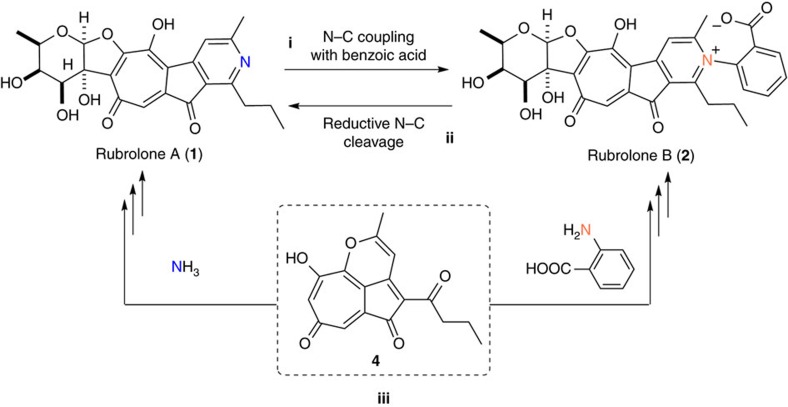
Different possible biosynthetic relationships between 1 and 2. (**i**) **2** being generated by the oxidative N–C coupling of benzoic acid and **1**, (**ii**) **1** generated by the reductive N–C cleavage of **2** and (**iii**) both **1** and **2** arising from divergent amination of **4**.

Having established the intermediacy of **3** and **4** in the biosynthesis of rubolone A (**1**), we next sought to understand the biosynthetic incorporation of the benzoic acid moiety of rubrolone B (**2**). Although a 2-l fermentation of the wild type strain *S*. sp. KIB-H033 produces large quantities of **1** (60 mg l^−1^) and **2** (50 mg l^−1^) under even suboptimal conditions^5^, the heterologous expression strain *S. albus* 9B10 showed a similar titre to the WT strain for **1**, but only a very small amount of **2** (∼0.2 mg) was isolated from 2-l fermentation broth. The varying titre ratios of rubrolones A (**1**) and B (**2**) in different strains may arise from biosynthetic pathways in which (i) one is the precursor of the other or (ii) they arise from divergent amination of a common intermediate **4** (Fig. 5). For example, if **1** is the precursor of **2**, then the benzoic acid moiety would need to be attached to the nitrogen atom of **1** by oxidative N–C coupling to afford **2** (Fig. 5). Conversely, initial production of **2** would require subsequent N–C bond cleavage to liberate the benzoic acid moiety and yield **1** (Fig. 5). Alternatively, **3** and **4** may generate **1** and **2** via divergent elaboration of **4** with either ammonia or anthranilic acid as respective nitrogen sources (Fig. 5). Interestingly, the latter hypothesis is favored by the presence of large quantities of anthranilic acid only in the wild type strain, which produced large quantities of **2**. In contrast, anthranilic acid was not detected in the heterologous expression strain *S. albus* 9B10, which yielded only trace amount of **2**.

To begin to differentiate among these three possible routes, we fed ^15^N-labelled anthranilic acid to the fermentation media of wild type strain *S*. sp. KIB-H033 at 24 h (the time point at which **1** and **2** could first be detected) and 48 h after inoculation, and then isolated and analysed **1** and **2** by HRMS, ^15^N NMR and ^1^H–^15^N long range correlation experiments to characterize ^15^N incorporation. Consistent with the divergent pathway hypothesis, adding 200 mg l^−1^ of ^15^N-labelled anthranilic acid to cultures yielded ^15^N enrichment in **2** but not **1** (Supplementary Fig. 5). Moreover, adding exogenous anthranilic acid to the heterologous expression strain *S. albus* 9B10 completely restored production of **2** (Fig. 2, trace IX). In summary, the incorporation of exogenous anthranilic acid supports its role as a biosynthetic intermediate en route to rubrolone B (**2**).

### The pyridyl moiety arises from non-enzymatic amination of 4

The divergent biosynthetic pathways for **1** and **2** implied by anthranilic acid incorporation, together with the production of intermediate **4** in sugar biosynthetic gene knockout mutants, strongly suggested that **4** can react with free ammonia and anthranilic acid to generate the pyridine ring and pyridine inner salt moiety, respectively (Fig. 5). To further test this hypothesis, we fed 200 mg l^−1^ anthranilic acid to the **4**-producing mutant *S. albus* 9B10-Δ*S1*. HPLC analyses of the fermentation extracts revealed production of a new compound **8** (Fig. 2, trace X), of which we isolated 7.9 mg from a 2-l culture. Evaluation of the HRESIMS at *m/z* 416.1140 [M−H]^−^ for C_24_H_18_NO_6_ and 1D and 2D NMR spectra of **8** (Supplementary Table 3; Supplementary Fig. 3) in comparison to the NMR data of **2** and **3** (Supplementary Table 2) led to the structural assignment of **8** (Fig. 4) as the aglycone of rubrolone B (**2**).

The chemical structure and reactivity of the biosynthetic intermediate **4**, the presence of excess anthranilic acid in the fermentation culture of the wild type strain *S*. sp. KIB-H033, and the absence of suitable gene candidates in the *rub* cluster together suggest that amination may occur non-enzymatically. This would enable production of designer RUB analogues with altered benzoate moieties if the RubS7 glycosyltransferase were sufficiently promiscuous to attach different aglycones to the NDP sugar. Feeding F, Cl, Br or OH substituted anthranilic acids to the culture medium of *S. albus* 9B10 led to the production of several novel rubrolones based on HRMS data (Supplementary Figs 6–11), two of which (Fig. 4) were isolated and characterized in detail by NMR (rubrolones C (**10**) and D (**11**), Supplementary Fig. 3; Supplementary Table 4).

**Figure 6:**
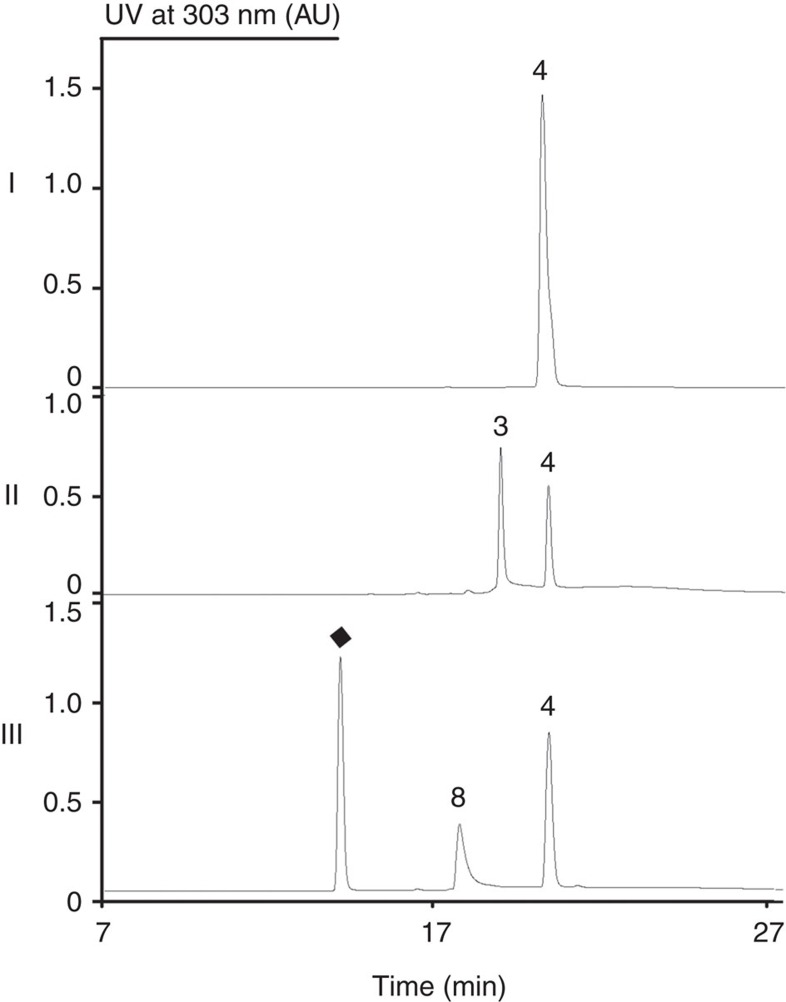
HPLC analysis of *in vitro* chemical conversions |: **4** was incubated with buffer only; II: **4** was incubated with ammonium acetate; and III: **4** was incubated with anthranilic acid (black diamond).

Encouraged by the *in vivo* results, we next sought to obtain evidence for the non-enzymatic reactions by performing *in vitro* reactions between the isolated biosynthetic intermediate **4** and either ammonia or anthranilic acid analogues in phosphate buffer at pH 8.0 to mimic the fermentation media. As expected, incubation of **4** with ammonium acetate yielded a new HPLC peak with a retention time corresponding to that of the intermediate **3** (Fig. 6, trace II), while a peak corresponding to **8** appeared when anthranilic acid was used in place of ammonium acetate (Fig. 6, trace III). Furthermore, incubation of **4** with F, Cl, Br or OH substituted anthranilic acids yielded new rubrolone aglycones based on HRMS data (Supplementary Figs 12–15). These results clearly demonstrate the non-enzymatic divergent elaboration of **4** with either ammonia or anthranilic acid analogues to afford diverse rubrolone aglycones.

## Discussion

Rubrolones are tropolonoid natural products with an unusual carbon skeleton of polyketide origin containing a unique 2,3,4,6-tetra-substituted pyridine ring or pyridine inner salt moiety. In this work, we have identified a 22 ORF rubrolone biosynthetic gene cluster and performed a series of experiments that are consistent with the following features of rubrolone biosynthesis (Fig. 3): (i) a KSIII generates a butyryl-ACP starter unit, and a type II PKS catalyses seven rounds of chain extension; (ii) oxidative enzymes RubB and RubC together catalyse the complex oxidative rearrangements leading to tropolone ring formation; (iii) glucose-1-phosphate is transformed to dTDP-2-keto-D-fucose, which is then attached to the fully maturated pyridyl-containing rubrolone aglycone by glycosyltransferase RubS7 and (iv) **4** is a common intermediate in rubrolone biosynthesis, from which structural divergence to rubolones A (**1**) and B (**2**) occurs by non-enzymatic condensation with respective amine donors ammonia and anthranilic acid.

Several experimental results demonstrated that free ammonia and anthranilic acid can react with **4** to generate the pyridyl moieties of rubrolone A (**1**) and rubrolone B (**2**), respectively. First, only **2** was enriched with ^15^N after feeding ^15^N-labelled anthranilic acid to the wild type strain, demonstrating that direct conversion of **2** to **1** via N–C bond cleavage did not occur (Fig. 5). Second, exogenous anthranilic acid could completely restore production of **2** to the anthranilic acid-deficient heterologous expression strain *S. albus* 9B10 (Fig. 2, trace IX). Third, the rubrolone B aglycone **8** (Fig. 4; Supplementary Fig. 3) was directly observed when anthranilic acid was supplemented in the fermentation media of the sugar biosynthesis-deficient strain *S. albus* 9B10-Δ*S1*. Overall, this data clearly implicates a biosynthetic pathway that diverges from **4** in the presence of ammonia or anthranilic acid to respectively afford either rubrolone A (**1**) or rubrolone B (**2**).

Although our *in vivo* data support divergent amination of **4**, the absence of suitable aminotransferase enzymes in the *rub* gene cluster suggested a non-enzymatic mechanism of pyridine formation. This hypothesis was proven by observing *in vitro* transformations of **4** to either **8** or **3** in the presence of anthranilic acid or ammonium acetate, respectively. Non-enzymatic reactions are increasingly being found in natural product biosynthetic pathways and have been exploited to expand chemical diversity of several classes of natural products. In this work, we demonstrated the facile non-enzymatic incorporation of several substituted anthranilic acids to explore rubrolone chemical space. Similar non-enzymatic condensations between carbonyl and amine functional groups are well known in natural products biosynthesis and have afforded discoipyrroles^38^, ammosamides^39^, elansolids^40^ and jadomycins^41^,^42^. Non-enzymatic Diels–Alder reactions have been widely observed en route to natural products such as paracaseolide A (ref. 43). Additional pericyclic reactions have been observed to generate impressive diversity of fungal polyketides by exploiting the intrinsic reactivity of an isochromene intermediate^44^. In summary, our non-enzymatic incorporation of various amine donors into the rubrolone scaffold highlights the potential for further non-enzymatic elaboration of rubrolones.

Most alkaloid pyridine ring biosyntheses involve proposed cyclization between carbonyl and amine functional groups, with one recent exception being an enzyme-catalysed formal [4+2] cycloaddition between two dehydroalanines and subsequent aromatization en route to the pyridyl moiety of thiocillins^45^. We propose a non-enzymatic rubrolone pyridine construction that does not involve an amino acid precursor, but instead features the capture of free amines from solution by the reactive 1,5-dione moiety of **4** (Supplementary Fig. 16A). Specifically, we propose that the large distance between the C-15 ketone and C-3 could require hydrolytic ring opening and isomerization to the 1,5-dicarbonyl intermediate **4a**, which rapidly interconverts with **4** as shown by the disappearance of the H-2 and H-4 NMR signals of **4** in D_2_O (Supplementary Fig. 16B). The reactive 1,5-dicarbonyl intermediate **4a** would readily capture free amines to generate the ketimine **4b**. Subsequent dehydration and aromatization would afford the rubrolone pyridyl moiety **4c** (Supplementary Fig. 16A). The condensation of 1,5-diones with amines is a general approach to pyridyl formation that has been widely exploited in chemical synthesis^46^,^47^,^48^, but suffers from required extremes of temperature and pH (refs 47, 48). Nature has solved this problem in rubrolone biosynthesis by generating a highly reactive 1,5-dione intermediate to non-enzymatically capture free amines en route to pyridyl assembly. We propose that such non-enzymatic capture of diverse free amine metabolites may represent a general biosynthetic strategy for furnishing pyridine rings in natural products such as cimicifugadine^49^, beatrines^50^ and cassiarins^51^ (Supplementary Fig. 17), and this study sets the stage for further discovery of an expanded set of diverse pyridine-containing natural products.

## Methods

### General materials and experimental procedures

All reagents, solvents and restriction enzymes were purchased from standard commercial sources and used directly. DNA isolation and manipulation in *Escherichia coli* (*E. coli*) and *Streptomyces* were performed according to standard protocols. PCR amplifications were carried out on Biometra professional thermocycler (070-851, An Analytik Jena Company, Germany) using either Taq DNA polymerase (TaKaRa) or Pfu DNA polymerase (Thermo scientific). Primer synthesis and DNA sequencing were performed at Beijing Zixi Bio Tech Co., Ltd.

Column chromatography (CC) was performed using silica gel 60 RP-18 (EMD Chemicals Inc., Germany), Sephadex LH-20 (GE Healthcare Bio-Sciences Corp., Piscataway, NJ, USA) and D101 macroporous absorbent resin (Tianjin Haiguang Chemical Co. Ltd.). Semipreparative HPLC was conducted on a HITACHI Chromaster system equipped with a DAD detector, a YMC-Triart C_18_ column (250 mm × 10 mm i.d., 5 μm), and a flow rate of 3.0 ml min^−1^ at a column temperature of 25 °C. HPLC analysis was carried out on HITACHI Chromaster system equipped with a DAD detector, a YMC-Triart C_18_ column (250 mm × 4.6 mm i.d., 5 μm, Japan). NMR spectra were recorded in dimethylsulphoxide (DMSO)-*d*_6_ or CD_3_OD using a Bruker AVANCE III-600 spectrometer (Bruker Corp., Switzerland), and tetramethylsilane was used as internal standard. HRESIMS data were obtained using an Agilent G6230 Q-TOF mass instrument (Agilent Corp., USA).

### Strain culture conditions and plasmids

The bacterial strains and plasmids used in this study are summarized in Supplementary Table 5. The primer sequences are listed in Supplementary Table 6. *E. coli* strains or *E. coli* strains carrying plasmids were grown in Luria-Bertani medium with appropriate antibiotic selection. *Streptomyces* stains were routinely cultured in mannitol soya flour (MS) medium (Soybean flour 20 g l^−1^, mannitol 20 g l^−1^, agar 20 g l^−1^, pH 7.2) with appropriate antibiotic selection. *E. coli*–*Streptomyces* conjugations were performed on MS solid medium freshly supplemented with 20 mM MgCl_2_, and TSB liquid medium (tryptone 17 g l^−1^, phytone 3 g l^−1^, NaCl 5 g l^−1^, K_2_HPO_4_ 2.5 g l^−1^, glucose 2.5 g l^−1^, pH 7.2) was used as seed medium, and the rubrolone production medium (dextrin 40 g l^−1^, lactose 40 g l^−1^, yeast extract 5 g l^−1^, MOPS sodium salt 20 g l^−1^, ammonium acetate 1 g l^−1^, pH 7.2) was used for fermentation. For selection of recombinant clones, antibiotics were supplemented as follows: kanamycin (50 μg ml^−1^), apramycin (50 μg ml^−1^), erythromycin (200 μg ml^−1^) and carbenicillin (100 μg ml^−1^).

### Genome sequencing and genomic library construction

The genome sequencing of *S*. sp. KIB-H033 was performed with the Illumina Genome Analyzer (Illumina, San Diego, CA) by BGI (BGI-Shenzhen, China). High-molecular-mass genomic DNA isolated from *S.* sp KIB-H033 was used to construct small (200–500 bp) and large (2–3 kb) random sequencing libraries. The reads were first filtered and assembled into 85 contigs using SOAPdenovo (http://soap.genomics.org.cn/). We then used the paired-end information, step-by-step from the shortest (200 bp) to the longest (2,000 bp) insert size, to join the contigs into 17 scaffolds. Putative protein-coding sequences were predicted using the GLIMMER program, and the annotation was accomplished by BlastP analysis of sequences in the Nr, Nt, and SwissProt databases and by manual curation of the outputs of a variety of similarity searches. According to standard procedures, the genomic DNA was partially digested with *Mbo*I. Then the 30–45 kb DNA fragments were isolated and ligated to cosmid pJTU2554. MaxPlax Lambda packaging extracts were used to packaging. About 2,000 *E. coli* clones were picked, and stored in 20 96-well microplates at −80 °C.

### Heterologous expression and mutant construction

The positive cosmids were introduced to the selected model *Streptomyces* host by *E. coli*–*Streptomyces* conjugation according to the standard protocol^21^. Cosmid 9B10 which contains the whole *rub* gene cluster was used for the construction of gene deletion mutant. Since the cosmid pJTU2554 has the apramycin resistance gene *aac(3)IV* and *oriT*, the target gene was replaced with erythromycin resistance cassette *ermE* which was amplified from plasmid pJTU6722 (provided by Prof Meifeng Tao (Shanghai Jiao Tong University, China)) using λ RED-mediated PCR targeting mutagenesis with corresponding primers (Supplementary Table 6). To isolate the markerless gene deletion mutant, the gene inactivation cosmids were introduced into *E. coli* DH5α/BT340 via electroporation; incubation overnight at 42 °C to induce expression of FLP recombinase resulted in the loss of *ermE* cassette and the generation of an 81-bp in-frame scar, affording final markerless gene inactivation vectors. All the gene inactivation vectors used in this study were constructed by the same strategy (Supplementary Figs 18–28).

The gene inactivation vectors were introduced by transformation into *E. coli* ET12567/pUZ8002 and the vectors were then transferred to *S. albus* J1074 by intergeneric conjugation according to the standard methods^21^. The spores of *S. albus* J1074 were produced in the MS medium. *E. coli*–*Streptomyces* conjugations were performed on MS solid medium freshly supplemented with 20 mM MgCl_2_. The mutants were selected with 50 μg ml^−1^ apramycin after 4 days cultivation and subsequently identified through PCR with corresponding primers (Supplementary Table 6). Finally, 11 markerless gene inactivation mutants were constructed in this study.

### Chemical purification of compounds 1–8 and 10–11

For HPLC analysis of the fermentation extracts of engineered heterologous expression strains, the frozen spore stocks (100 μl) of strains were inoculated into 250 ml baffled-flask containing 50 ml of seed medium and shaken at 250 r.p.m. and 28 °C. After 30 h, an aliquot of seed culture (2.5 ml) was transferred into a 250 ml baffled-flask containing 50 ml of production medium and shaken at 250 r.p.m. and 28 °C for 4 days. The 50 ml fermentation culture broth was separated into mycelia and supernatant by centrifugation at 3,500*g* for 30 min. The resulting supernatant was subsequently mixed with D101 macroporous absorbent resin (5 ml) and stirred for 20 min. After removal of supernatant, the resin was washed with 20 ml H_2_O, and then 10 ml methanol. The 10 ml CH_3_OH was evaporated to dryness, and then the residues were dissolved in 1 ml CH_3_OH. The samples were applied to reverse-phase HPLC analysis eluted with a flow rate of 1 ml min^−1^ over a 28 min gradient as follows: *T*=0, 10% B; *T*=20, 100% B; *T*=24, 100% B; *T*=25, 10% B; *T*=28, 10% B (A, H_2_O; B, CH_3_OH), and the column temperature is 25 °C.

A 2 -l fermentation culture broth of *S. albus* 9B10 was separated into mycelia and supernatant by centrifugation at 3,500*g* for 30 min. The resulting supernatant was subsequently applied to a D101 macroporous absorbent resin column (0.5 l), the column was washed with H_2_O and then successively eluted with 100% methanol. The CH_3_OH fraction was evaporated to dryness, and the residue was dissolved in 50% CH_3_OH/H_2_O and subjected to Sephadex LH-20 CC eluting with 50% CH_3_OH/H_2_O to yield 18 fractions. All the fractions were analysed by HPLC and combined. Selected fractions contained the rubrolone analogues were further purified by semipreparative C_18_ HPLC to yield compounds **1** (26.8 mg) and **2** (∼0.2 mg).

A 2-l fermentation culture broth of *S. albus* 9B10-Δ*S1* was separated into mycelia and supernatant by centrifugation at 3,500*g* for 30 min. The resulting supernatant was subsequently applied to a D101 macroporous absorbent resin column (0.5 l), the column was washed with H_2_O and then successively eluted with 100% methanol. The CH_3_OH fraction was evaporated to dryness, and the residue was dissolved in CH_3_OH and subjected to Sephadex LH-20 CC eluting with CH_3_OH to yield 21 fractions. The fractions contained the compounds **3** and **4** were combined by HPLC analysis. Finally, the compounds **3** (15.6 mg) and **4** (33.7 mg) were purified by semipreparative C_18_ HPLC.

Compound **3**: Purple red powder; ^1^H NMR data was summarized in Supplementary Table 2 (Supplementary Fig. 29); HRESIMS *m/z* 298.1077 [M+H]^+^ (calculated for C_17_H_15_NO_4_, 298.1074), see Supplementary Fig. 30.

Compound **4**: Purple red powder; ^1^H, ^13^C, COSY and HMBC NMR data was summarized in Supplementary Table 2 (Supplementary Figs 31–35); HRESIMS *m/z* 297.0780 [M−H]^−^ (calculated for C_17_H_14_O_5_, 297.0769), see Supplementary Fig. 36.

A 5-l fermentation culture broth of *S. albus* 9B10-Δ*B* was separated into mycelia and supernatant by centrifugation at 3,500*g* for 30 min. The resulting supernatant was extracted with ethyl acetate (3 l) for three times, then evaporation of EtOAc generated the residue. This residue was dissolved in CH_3_OH and mixed with an appropriate amount of polyamide, then the sample was applied to a reverse-phase C_18_ column and subsequently eluted with gradient CH_3_OH–H_2_O (from 10:90 to 100:0) to afford 27 fractions, each of which was analysed by HPLC. The fractions containing the **5**–**7** were combined and evaporated, then subjected to Sephadex LH-20 CC eluting with CH_3_OH to yield 25 fractions, and combined by HPLC analysis. The compounds **5** (6.9 mg), **6** (11.3 mg), and **7** (4.2 mg) were finally purified by semipreparative C_18_ HPLC from selected fractions.

Compound **5**: Yellow powder; ^1^H and ^13^C NMR data was summarized in Supplementary Table 2 (Supplementary Figs 37 and 38); HRESIMS *m/z* 297.0770 [M−H]^−^ (calculated for C_17_H_14_O_5_, 297.0769), see Supplementary Fig. 39.

Compound **6**: Yellow powder; ^1^H, ^13^C, COSY and HMBC NMR data was summarized in Supplementary Table 3 (Supplementary Figs 40–44); HRESIMS *m/z* 341.0669 [M−H]^−^ (calculated for C_18_H_14_O_7_, 341.0667), see Supplementary Fig. 45.

Compound **7**: White powder; ^1^H, ^13^C, COSY, and HMBC NMR data was summarized in Supplementary Table 3 (Supplementary Figs 46–50); HRESIMS *m/z* 319.1189 [M−H]^−^ (calculated for C_17_H_20_O_6_, 319.1187), see Supplementary Fig. 51.

The *S. albus* 9B10-Δ*S1* was grown in seed medium for 30 h, then about 2.5 ml seed culture (5% inoculums) was inoculated into 50 ml production medium at 30 °C. The anthranilic acid was added to the fermentation media at 24 h (10 mg in 100 μl DMSO) after inoculation, and then fermented for additional 3 days The extract was analysed by HPLC and found the production of compound **8**. For isolation of compound **8**, a 2-l fermentation culture broth of *S. albus* 9B10-Δ*S1* feeding with anthranilic acid (200 mg l^−1^) was separated into mycelia and supernatant by centrifugation at 3,500*g* for 30 min. The resulting supernatant was subsequently applied to a D101 macroporous absorbent resin column (0.5 l), the column was washed with H_2_O and then successively eluted with 100% CH_3_OH. The CH_3_OH fraction was evaporated to dryness, and the residue was dissolved in CH_3_OH and subjected to Sephadex LH-20 CC eluting with CH_3_OH to yield fractions, and combined by HPLC analysis. The fractions containing **8** was finally subjected to semipreparative C_18_ HPLC to yield the pure compound **8** (7.9 mg).

Compound **8**: Purple red powder; ^1^H, ^13^C, COSY, and HMBC NMR data was summarized in Supplementary Table 3 (Supplementary Figs 52–56); HRESIMS *m/z* 416.1140 [M−H]^−^ (calculated for C_24_H_19_NO_6_, 416.1140), see Supplementary Fig. 57.

The *S. albus* 9B10 was grown in seed medium for 30 h, then about 2.5 ml seed culture (5% inoculums) was inoculated into 50 ml production medium at 30 °C. After 24 h incubation, 2-amino-5-fluoro-benzoic acid (10 mg), 2-amino-5-chloro-benzoic acid (10 mg), 2-amino-5-bromo-benzoic acid (10 mg), 2-amino-5-hydroxy-benzoic acid (10 mg), 2-amino-3-chloro-benzoic acid (10 mg) and 2-amino-4-chloro-benzoic acid (10 mg) dissolved in 100 μl DMSO were added into culture, respectively, and fermented for additional 3 days. The extracts were analysed by HPLC and the HPLC peaks corresponding to rubrolone analogues **10**–**15** were collected for HRESIMS analysis. For isolation of compounds **10** and **11**, the 2-l fermentation culture broth of *S. albus* 9B10 feeding with 2-amino-5-fluoro-benzoic acid (200 mg l^−1^) and 2-amino-5-chloro-benzoic acid (200 mg l^−1^), respectively, were separated into mycelia and supernatant by centrifugation at 3,500*g* for 30 min. The resulting supernatant was subsequently applied to a D101 macroporous absorbent resin column (0.5 l), the column was washed with H_2_O and then successively eluted with 100% methanol. The CH_3_OH fraction was evaporated to dryness, and the residue was dissolved in CH_3_OH and subjected to Sephadex LH-20 CC eluting with CH_3_OH to yield fractions, and combined by HPLC analysis. The compounds **10** (9.2 mg) and **11** (4.7 mg) were finally purified by semipreparative C_18_ HPLC, respectively.

Compound **10**: Purple red powder; ^1^H, ^13^C, ^19^F, COSY and HMBC NMR data was summarized in Supplementary Table 4 (Supplementary Figs 58–63); HRESIMS *m/z* 580.1615 [M+H]^+^ (calculated for C_30_H_26_NO_10_F, 580.1614), see Supplementary Fig. 6.

Compound **11**: Purple red powder; ^1^H, ^13^C, COSY and HMBC NMR data was summarized in Supplementary Table 4 (Supplementary Figs 64–68); HRESIMS *m/z* 596.1319 [M+H]^+^ (calculated for C_30_H_26_NO_10_Cl, 596.1318), see Supplementary Fig. 7.

### Feeding the *S. albus* 9B10-Δ*E9* with compounds 3 and 4

The *S. albus* 9B10-Δ*E9* mutant was grown in seed medium for 30 h, then about 2.5 ml seed culture (5% inoculums) was inoculated into 50 ml production medium at 30 °C. After 24 h incubation, compounds **3** (6 mg) and **4** (6 mg) in 100 μl DMSO were added into culture, respectively, and fermented for additional 3 days. The extracts were analysed by HPLC.

### Feeding the *S. albus* 9B10 with anthranilic acid

The *S. albus* 9B10 was grown in seed medium for 30 h, then about 2.5 ml seed culture (5% inoculums) was inoculated into 50 ml production medium at 30 °C. After 24 h incubation, anthranilic acid (10 mg) in 100 μl DMSO was added into culture and fermented for additional 3 days. The extract was analysed by HPLC.

### Synthesis of ^15^N-anthranilic acid and feeding experiment

The ^15^N-labelled anthranilic acid was synthesized from ^15^N urea and phthalic anhydride by the modified Hofmann reaction^52^. Before isotope-labelled precursor feeding, the production of rubrolones was monitored and found that rubrolones was produced at around 24 h after the start of fermentation. So, the ^15^N-labelled anthranilic acid at a final concentration of (200 mg l^−1^) was fed to the fermentation media of the wild type strain *S*. sp. KIB-H033 at 24 and 48 h after inoculation. From 1-l fermentation culture broth, rubrolones A (10.8 mg) and B (12.6 mg) were isolated following the purification procedure previously published^5^. Both compounds were analysed by HRESIMS. Finally, the same amounts (8.0 mg) of **1** and **2** in 0.45 ml DMSO-*d*_6_ solvent were subjected to ^15^N NMR measurements to determine the ^15^N incorporation (Supplementary Figs 5 and 69).

### *In vitro* reactions

All reactions were performed in sodium phosphate buffer (pH 8.0, 45 mmol). A mixture of compound **4** (1.5 mg, 5.03 μmol) and ammonium acetate (1.95 mg, 25.14 μmol) or anthranilic acid (3.45 mg, 25.14 μmol) was suspended in 1.0 ml of phosphate buffer and the mixture was stirring at 30 °C for 4 days. After reaction, 10 μl mixture was subject to HPLC analysis eluted with a flow rate of 1 ml min^−1^ over a 28 min gradient as follows: *T*=0, 10% B; *T*=20, 100% B; *T*=24, 100% B; *T*=25, 10% B; *T*=28, 10% B (A, H_2_O; B, CH_3_OH), and the column temperature was 25 °C. The same condition was used for generation of new rubrolone aglycones **16**–**19** using the 5-F, -Cl, -Br or -OH substituted anthranilic acids as the amine donors. The HPLC peaks corresponding to **16**–**19** were collected for HRESIMS analysis.

### Measurement of ^1^H NMR spectra of 4 in D_2_O

Compound **4** (5 mg) in NMR tube was mixed with 450 μl D_2_O. The ^1^H NMR spectrum of **4** was measured every 2 h until the disappearance of H-2 and H-4 NMR signals in **4**, see Supplementary Figs 70–72.

### Data availability

Sequence data that support the findings of this study has been deposited in GenBank with accession codes KX218108. The authors declare that all other relevant data supporting the findings of this study are available within the article and its Supplementary Information Files and from the corresponding author upon reasonable request.

## Additional information

**How to cite this article:** Yan, Y. *et al*. Non-enzymatic pyridine ring formation in the biosynthesis of the rubrolone tropolone alkaloids. *Nat. Commun.* **7,** 13083 doi: 10.1038/ncomms13083 (2016).

## Supplementary Material

Supplementary Information Supplementary Figures 1-72, Supplementary Tables 1-6, Supplementary References.

## Figures and Tables

**Figure 1:**
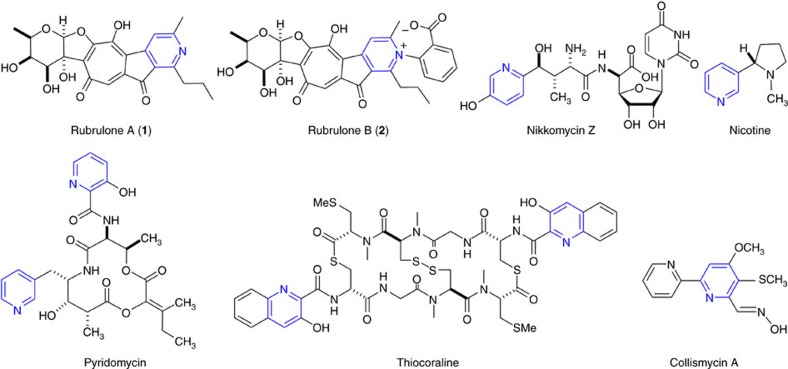
Chemical structures of rubrolones A (1) and B (2) and selected pyridine alkaloids. Pyridine rings are highlighted in blue.
