# Supplementary material for: Non-enzymatic pyridine ring formation in the biosynthesis of the rubrolone tropolone alkaloids
Source: Nat Commun. 2016 Oct 7;7:13083. doi: 10.1038/ncomms13083 (PMC5059770; doi:10.1038/ncomms13083)
Supplement: Supplementary Information — Supplementary Figures 1-72, Supplementary Tables 1-6, Supplementary References. [file ncomms13083-s1.pdf]

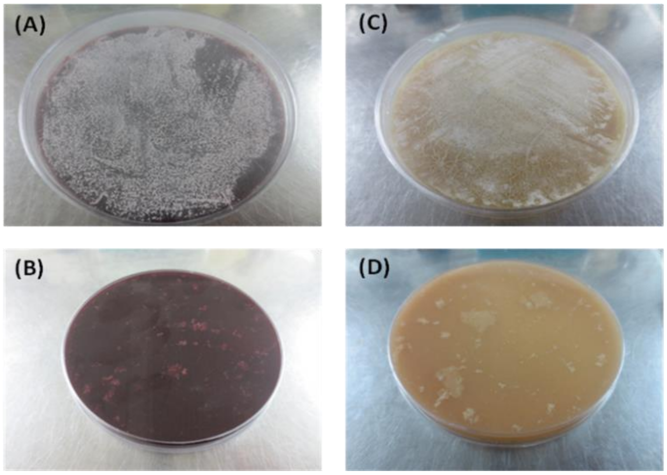

Supplementary Figure 1. The phenotype comparison of the engineered heterologous expression strains. (A) Front side of agar plate with strain *S. albus* 9B10; (B) Back side of agar plate with strain *S. albus* 9B10; (C) Front side of agar plate with strain *S. albus* pJTU2554; (D) Back side of agar plate with strain *S. albus* pJTU2554.

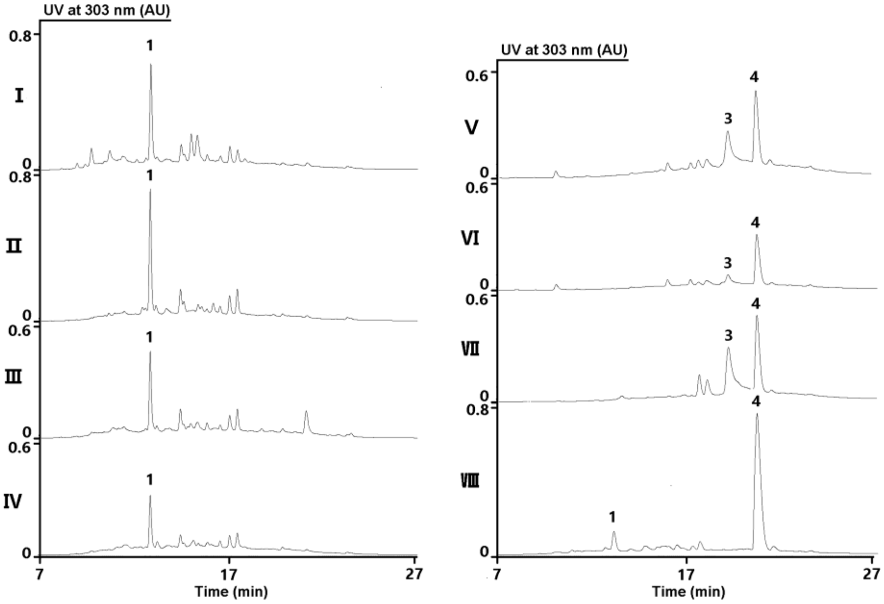

Supplementary Figure 2. HPLC profiles of the fermentation extracts of engineered heterologous expression strains. I: *S. albus* 9B10-1; II: *S. albus* 9B10-2; III: *S. albus* 9B10-Δ*E7*; IV: *S. albus* 9B10-Δ*A*; V: *S. albus* 9B10-Δ*S2*; VI: *S. albus* 9B10-Δ*S3*; VII: *S. albus* 9B10-Δ*S7*; VIII: *S. albus* 9B10 with reduced nitrogen source (removing the ammonium acetate and decreasing the yeast extract to 1g/L in the fermentation medium).

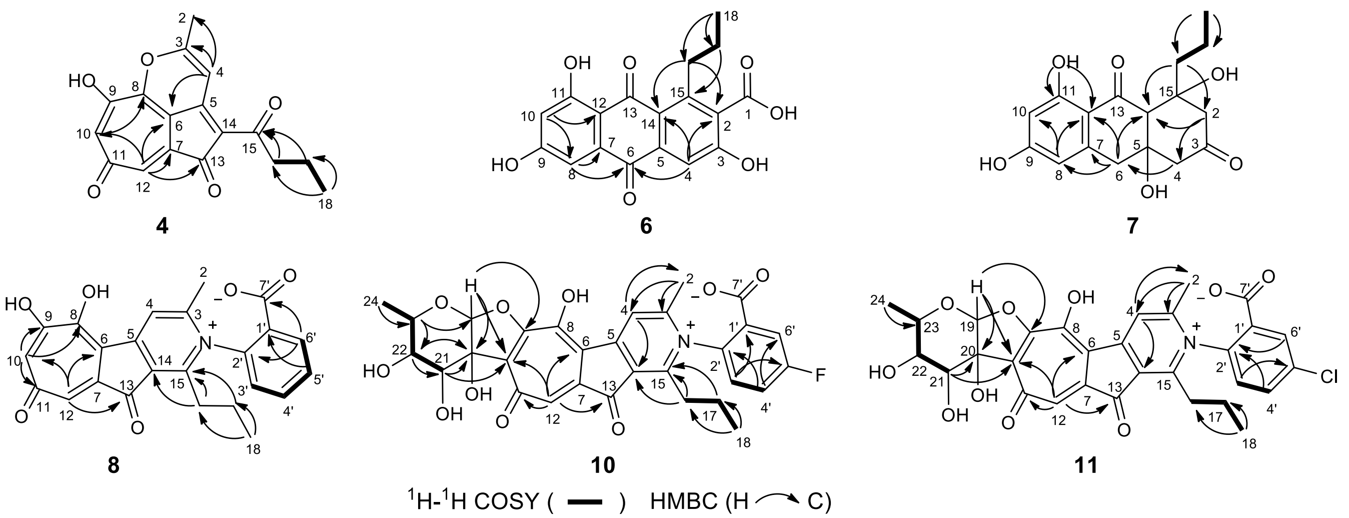

Supplementary Figure 3. Selected COSY and HMBC correlations for compounds **4**, **6-8**, and **10-11**.

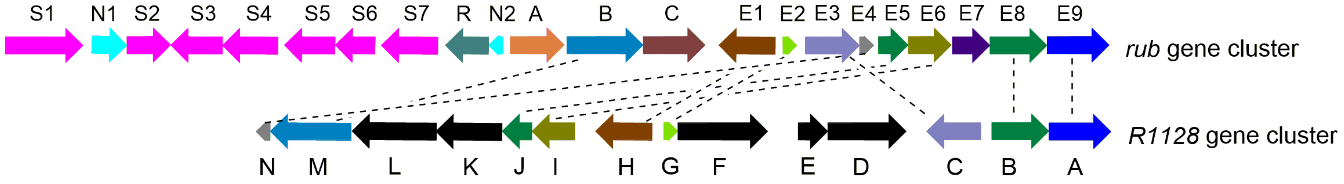

Supplementary Figure 4. Maps of the *rub* and *R1128* biosynthetic gene cluster.

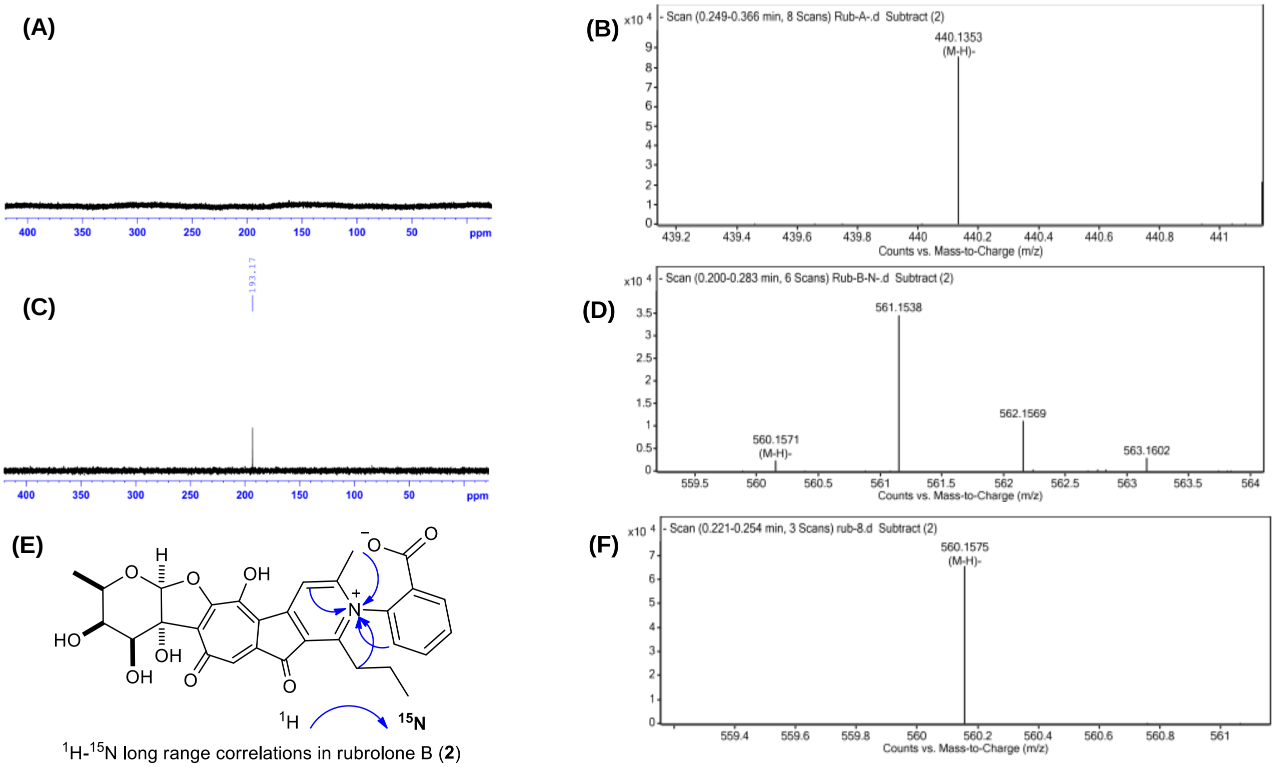

Supplementary Figure 5. MS and NMR data (measured in DMSO-d_6) of **1** and **2** with or without ^{15}N-labeled anthranilic acid feeding. (A) ^{15}N NMR spectrum of **1** obtained after feeding with ^{15}N-labeled anthranilic acid; (B) HRESIMS analysis of **1** obtained after feeding with ^{15}N-labeled anthranilic acid; (C) ^{15}N NMR spectrum of **2** obtained after feeding with ^{15}N-labeled anthranilic acid; (D) HRESIMS analysis of **2** obtained after feeding with ^{15}N-labeled anthranilic acid; (E) ^{1}H-^{15}N long range correlations of **2** obtained after feeding with ^{15}N-labeled anthranilic acid; (F) HRESIMS analysis of **2** without feeding of ^{15}N-labeled anthranilic acid.

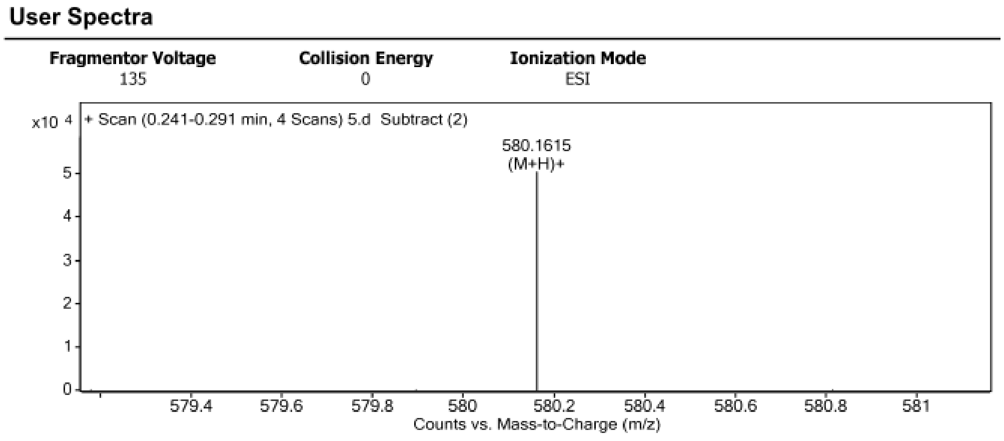

Supplementary Figure 6. HRESIMS analysis of compound **10**.

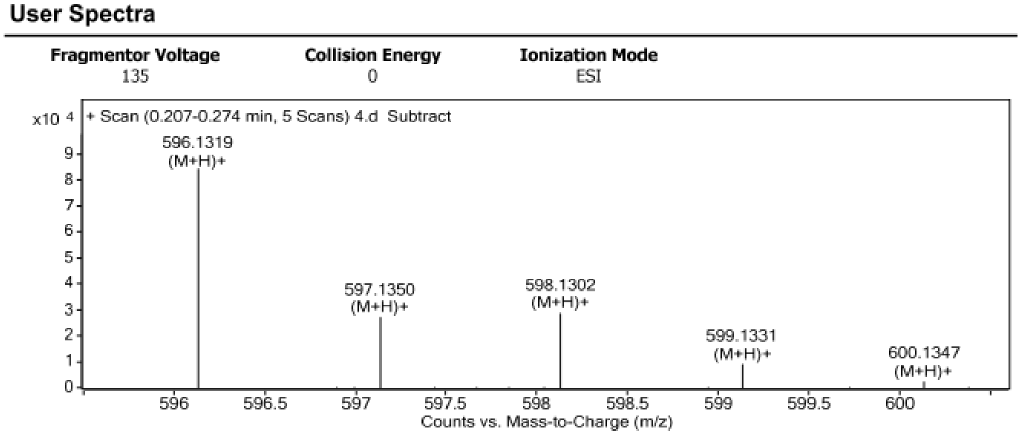

Supplementary Figure 7. HRESIMS analysis of compound **11**.

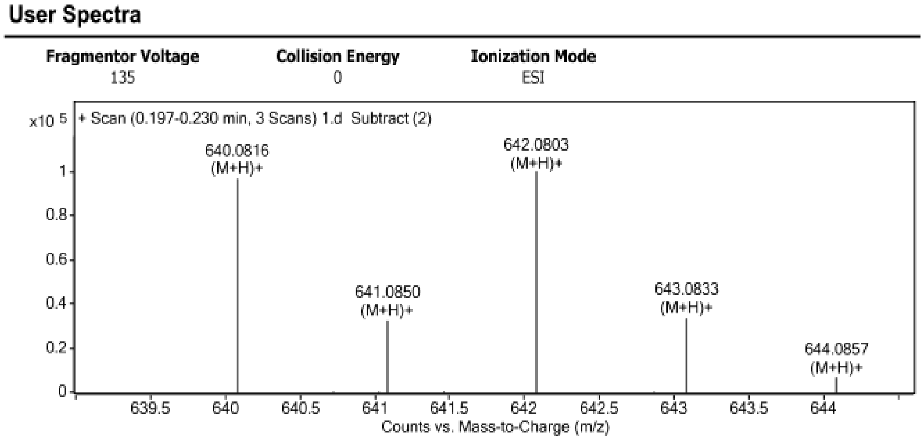

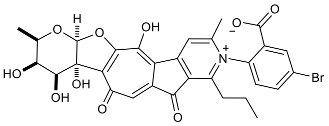

12

Supplementary Figure 8. HRESIMS analysis of compound **12**.

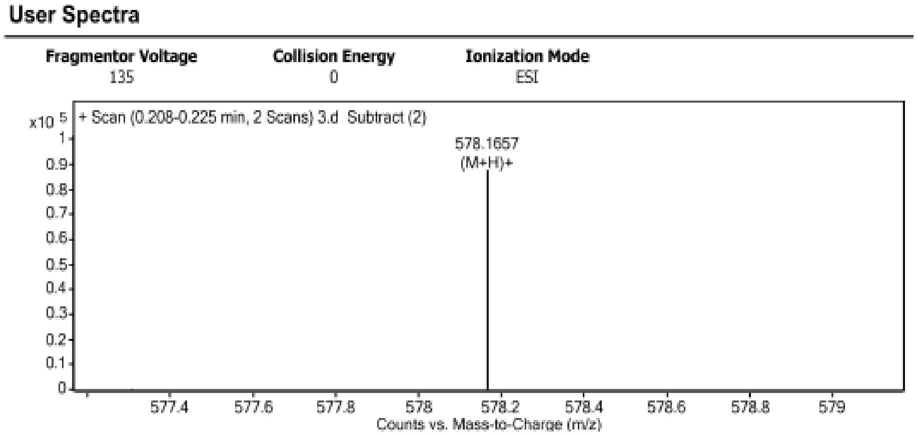

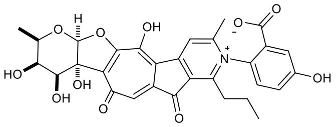

13

Supplementary Figure 9. HRESIMS analysis of compound **13**.

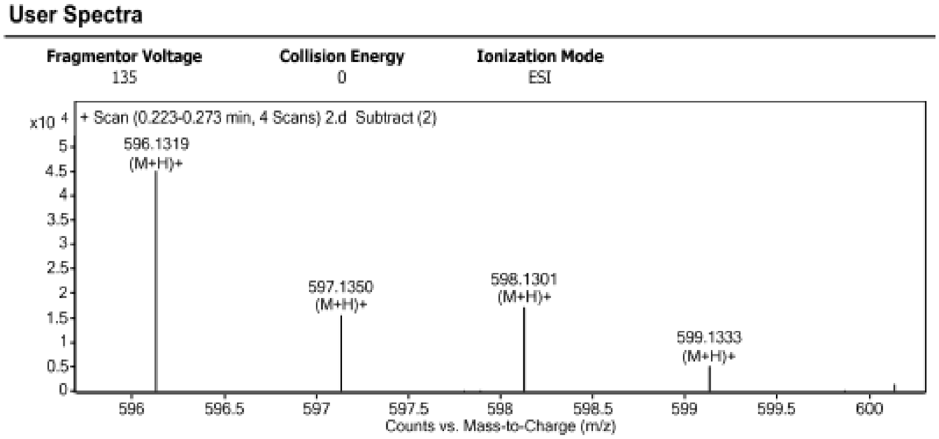

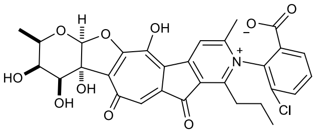

14

Supplementary Figure 10. HRESIMS analysis of compound **14**.

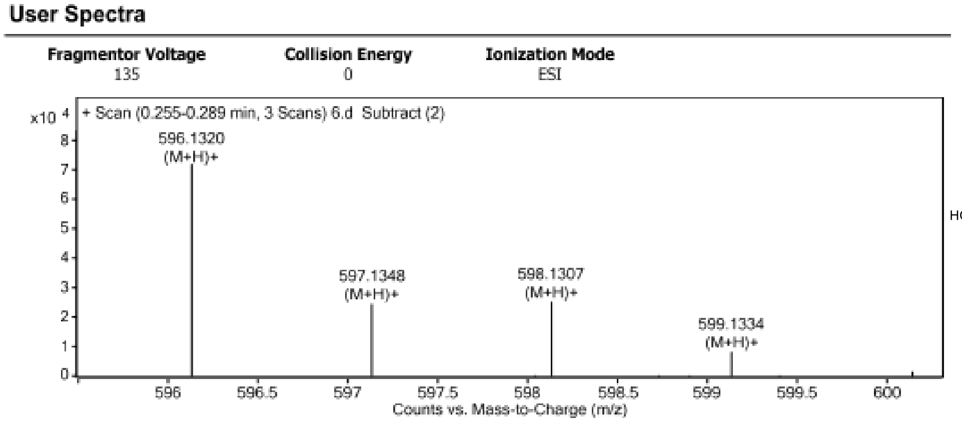

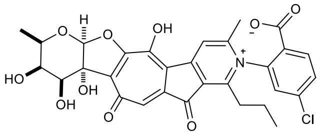

15

Supplementary Figure 11. HRESIMS analysis of compound **15**.

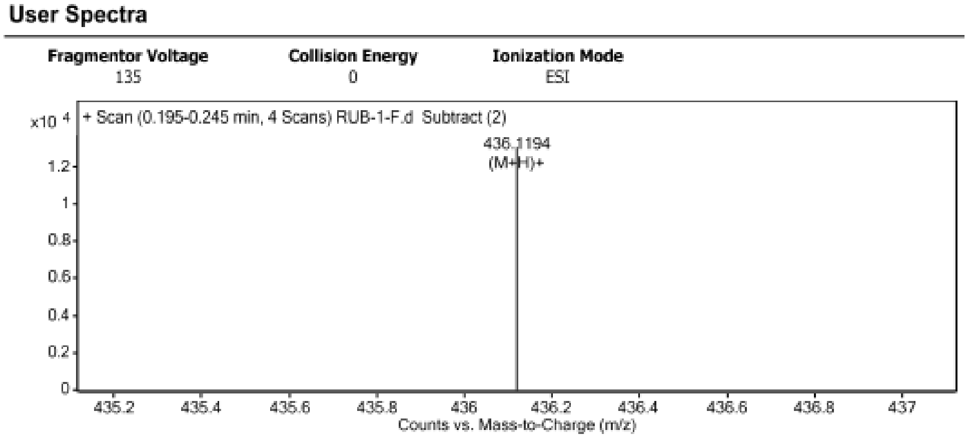

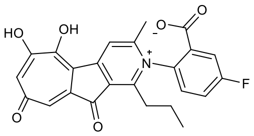

16

Supplementary Figure 12. HRESIMS analysis of compound **16**.

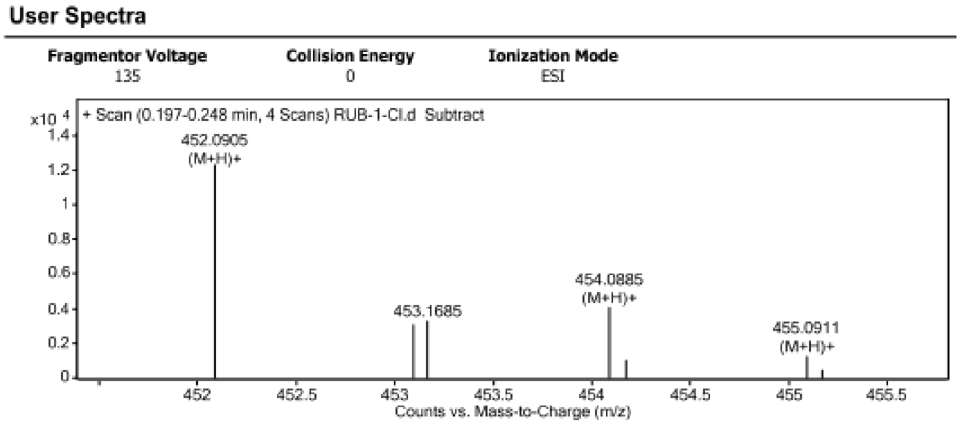

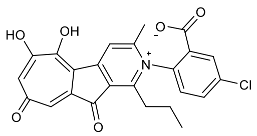

17

Supplementary Figure 13. HRESIMS analysis of compound **17**.

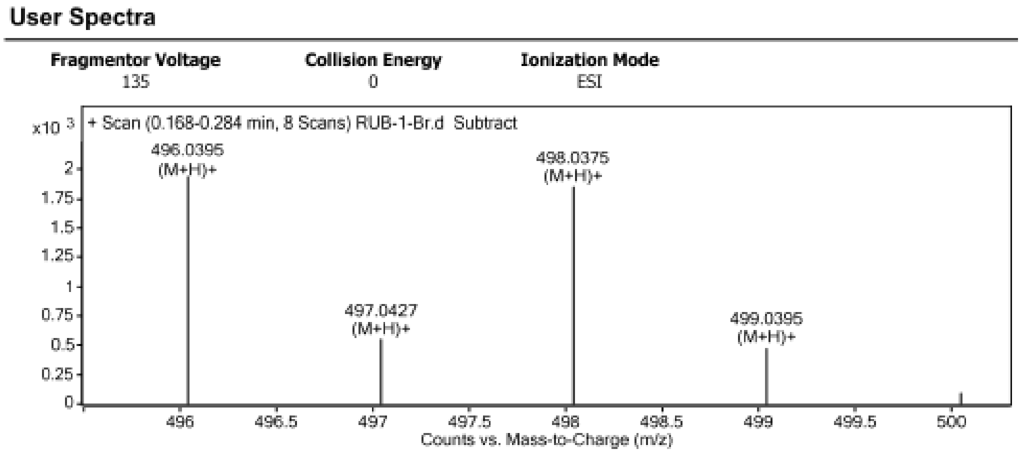

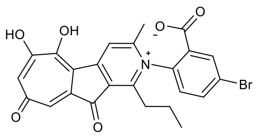

18

Supplementary Figure 14. HRESIMS analysis of compound **18**.

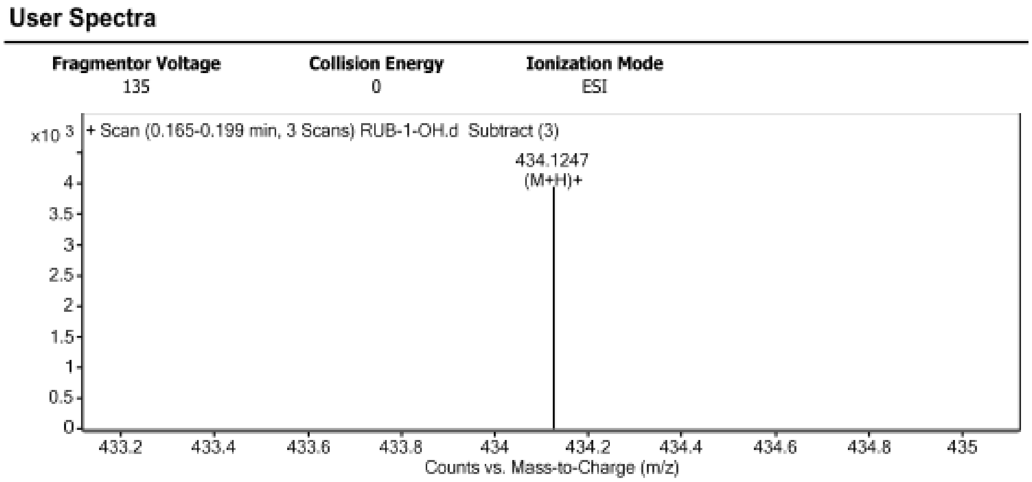

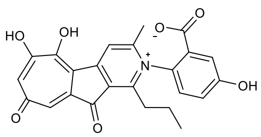

19

Supplementary Figure 15. HRESIMS analysis of compound **19**.

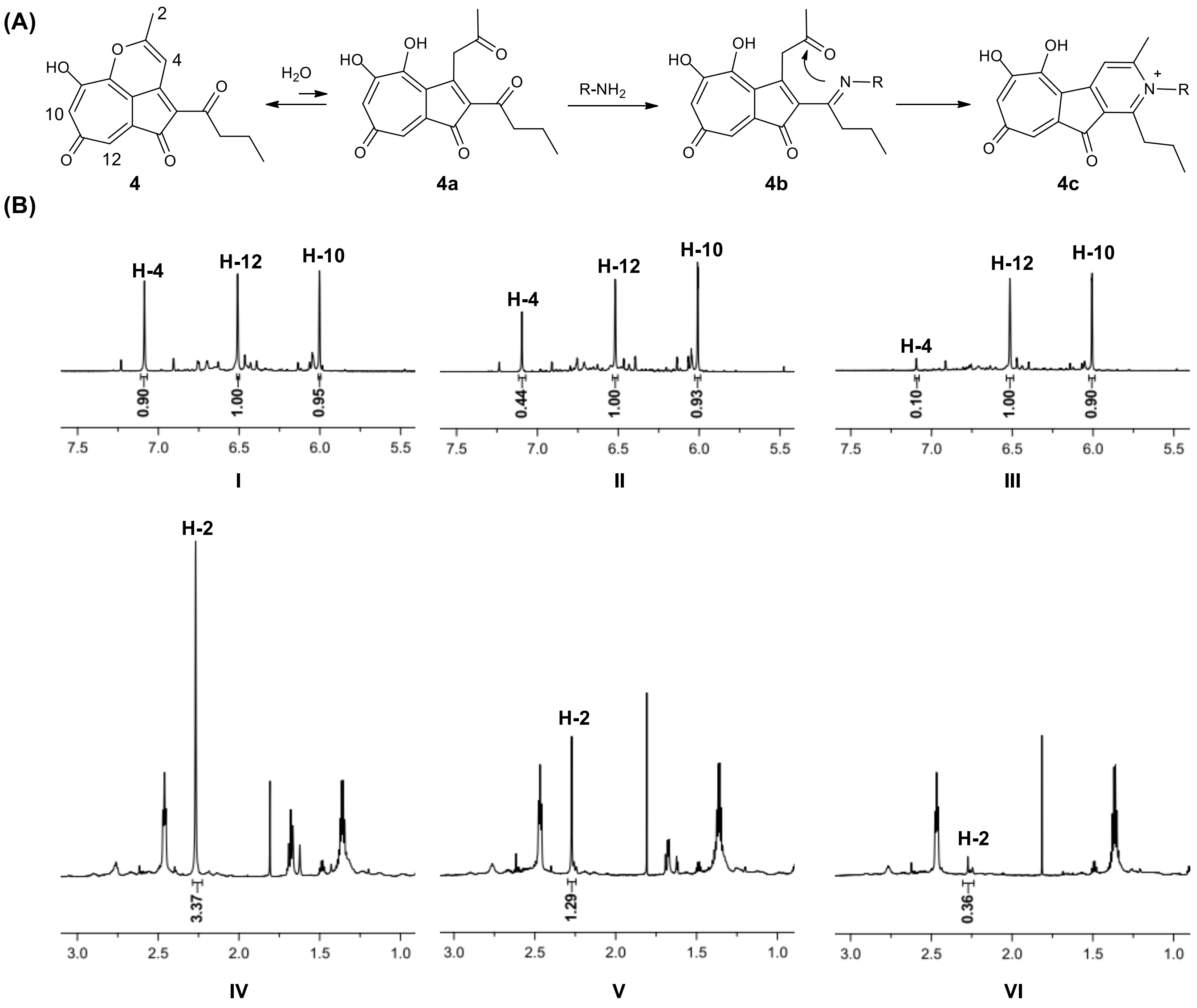

Supplementary Figure 16. (A) Proposed mechanism for the pyridyl moiety formation from intermediate **4**; (B) the enlarged ^{1}H NMR spectra of **4** in D_2O after 5 minutes (spectra I and IV, left), 2 hours (spectra II and V, middle) and 4 hours (spectra III and VI, right) incubation.

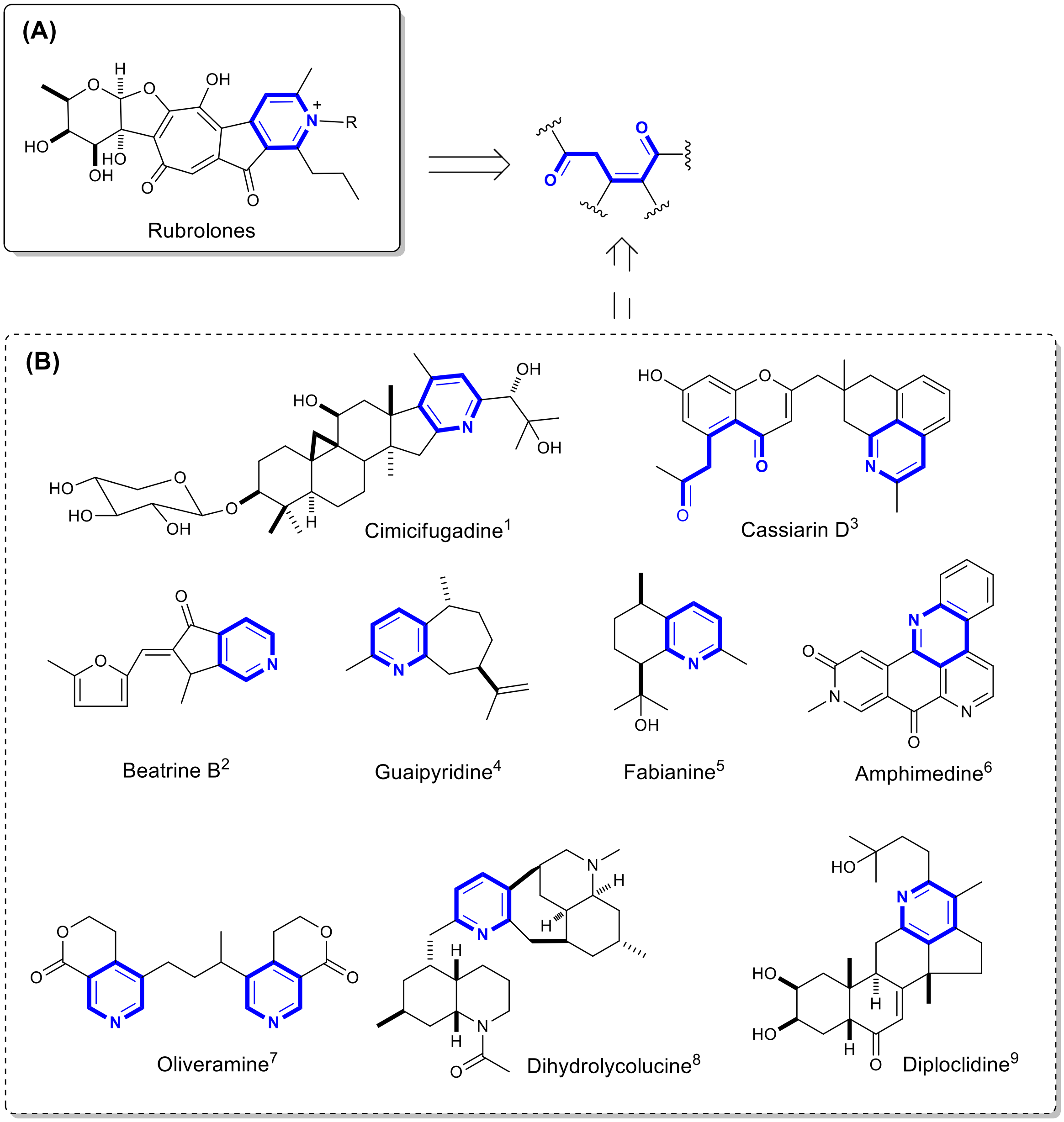

Supplementary Figure 17. The proposed central role of the reactive 1,5-dione moiety (blue) in accessing a diverse pyridine chemical universe including (A) rubolones and (B) selected natural alkaloids for which are potentially involved in the non-enzymatical pyridine formation using the intermediacy of the 1,5-dione moiety.

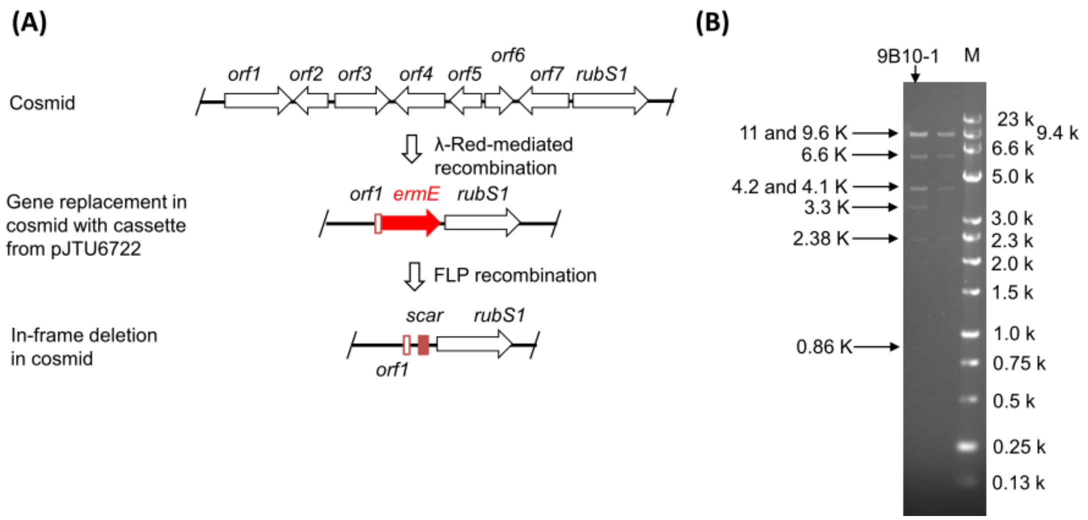

Supplementary Figure 18. Gene replacement of *orf1-7* using the PCR-targeting method. (A) Scheme for the construction of *orf1-7* replacement mutant. (B) Confirmation of plasmid p9B10-1 by restriction endonuclease BamHI. Lane M: DNA molecular ladder. Lane 9B10-1: Restriction fragments from the p9B10-1 (11259 bp, 9647 bp, 6601 bp, 4209 bp, 4123 bp, 3299 bp, 2376 bp, 858 bp, 20 bp).

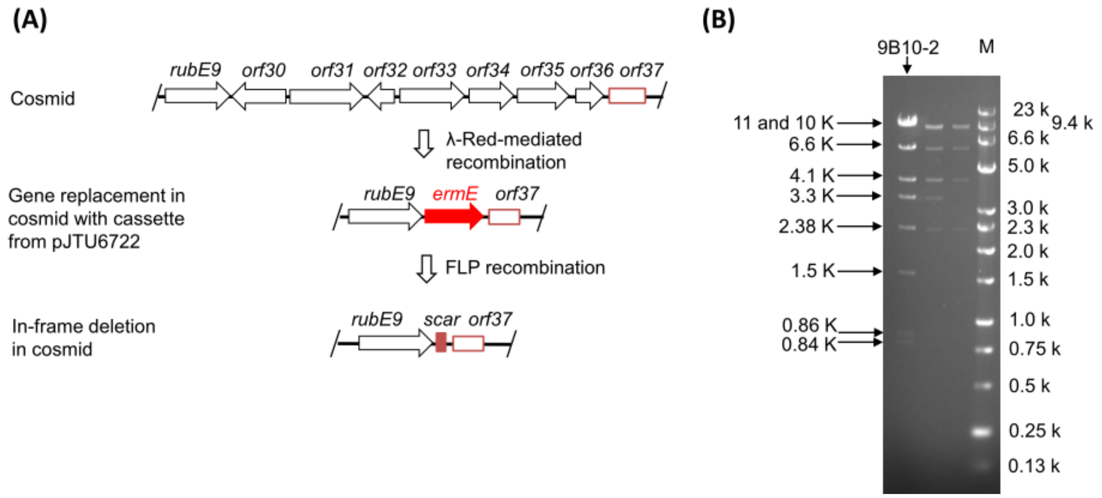

Supplementary Figure 19. Gene replacement of *orf30-37* using the PCR-targeting method. (A) Scheme for the construction of *orf30-37* replacement mutant. (B) Confirmation of plasmid p9B10-2 by restriction endonuclease BamHI. Lane M: DNA molecular ladder. Lane 9B10-2: Restriction fragments from the p9B10-2 (11259 bp, 10062 bp, 6601 bp, 4123 bp, 3299 bp, 2376 bp, 1473 bp, 858 bp, 844 bp, 37 bp).

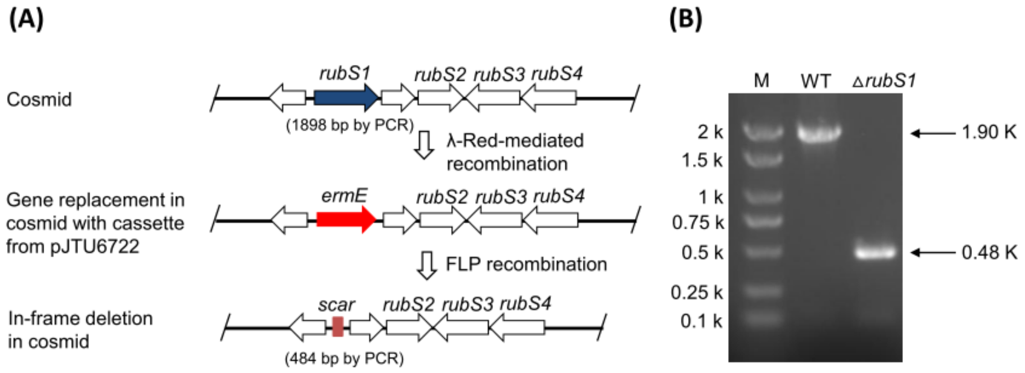

Supplementary Figure 20. Gene replacement of *rubS1* using the PCR-targeting method. (A) Scheme for the construction of *rubS1* replacement mutant. (B) Confirmation of mutant by PCR using the primers listed in supplementary Table 6. Lane M: DNA molecular ladder. Lane WT, PCR product from the *S. albus* 9B10. Lane Δ*rubS1*, PCR product from *S. albus* 9B10-Δ*S1* mutant.

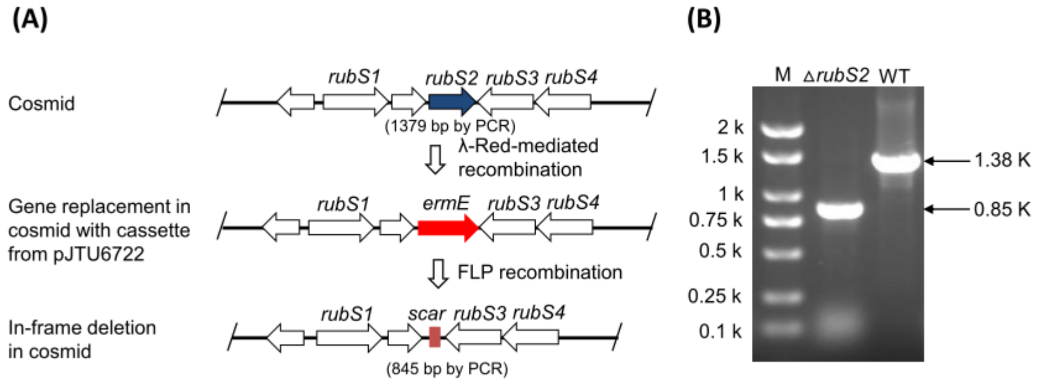

Supplementary Figure 21. Gene replacement of *rubS2* using the PCR-targeting method. (A) Scheme for the construction of *rubS2* replacement mutant. (B) Confirmation of mutant by PCR using the primers listed in supplementary Table 6. Lane M: DNA molecular ladder. Lane WT, PCR product from the *S. albus* 9B10. Lane Δ*rubS2*, PCR product from *S. albus* 9B10-Δ*S2* mutant.

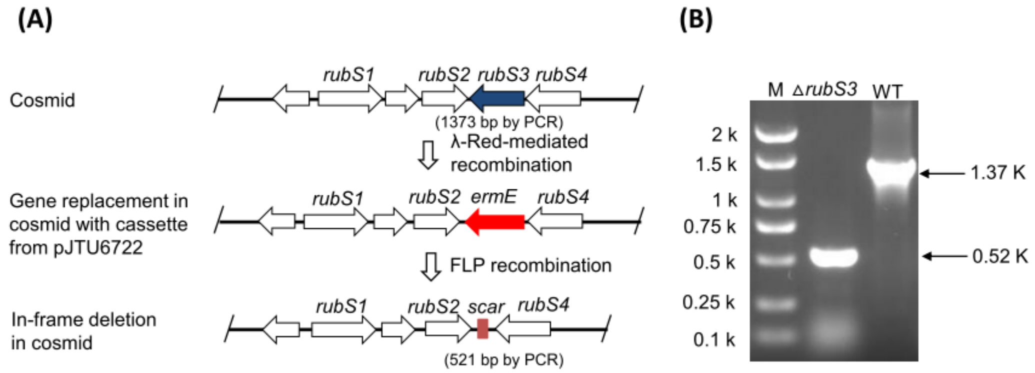

Supplementary Figure 22. Gene replacement of *rubS3* using the PCR-targeting method. (A) Scheme for the construction of *rubS3* replacement mutant. (B) Confirmation of mutant by PCR using the primers listed in supplementary Table 6. Lane M: DNA molecular ladder. Lane WT, PCR product from the *S. albus* 9B10. Lane Δ*rubS3*, PCR product from *S. albus* 9B10-Δ*S3* mutant.

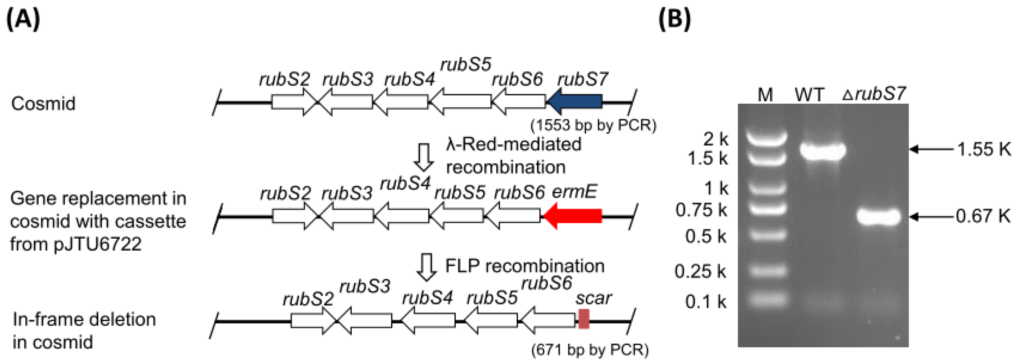

Supplementary Figure 23. Gene replacement of *rubS7* using the PCR-targeting method. (A) Scheme for the construction of *rubS7* replacement mutant. (B) Confirmation of mutant by PCR using the primers listed in supplementary Table 6. Lane M: DNA molecular ladder. Lane WT, PCR product from the *S. albus* 9B10. Lane Δ*rubS7*, PCR product from *S. albus* 9B10-Δ*S7* mutant.

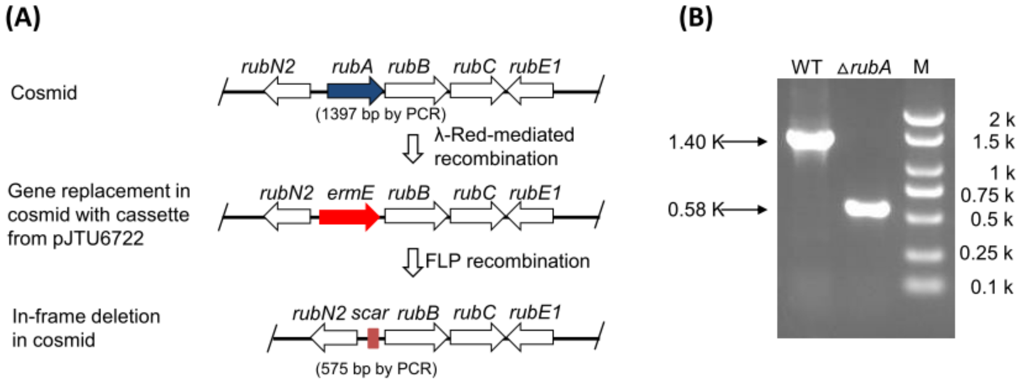

Supplementary Figure 24. Gene replacement of *rubA* using the PCR-targeting method. (A) Scheme for the construction of *rubA* replacement mutant. (B) Confirmation of mutant by PCR using the primers listed in supplementary Table 6. Lane M: DNA molecular ladder. Lane WT, PCR product from the *S. albus* 9B10. Lane Δ*rubA*, PCR product from *S. albus 9B10*-Δ*A* mutant.

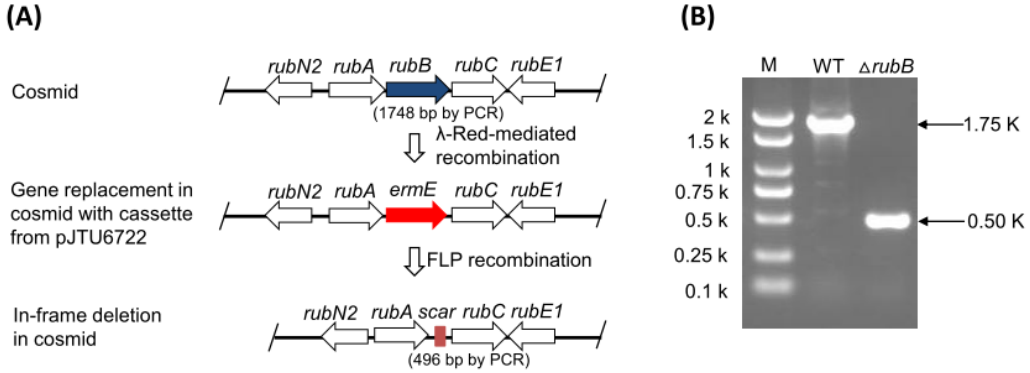

Supplementary Figure 25. Gene replacement of *rubB* using the PCR-targeting method. (A) Scheme for the construction of *rubB* replacement mutant. (B) Confirmation of mutant by PCR using the primers listed in supplementary Table 6. Lane M: DNA molecular ladder. Lane WT, PCR product from the *S. albus* 9B10. Lane Δ*rubB*, PCR product from *S. albus 9B10*-Δ*B* mutant.

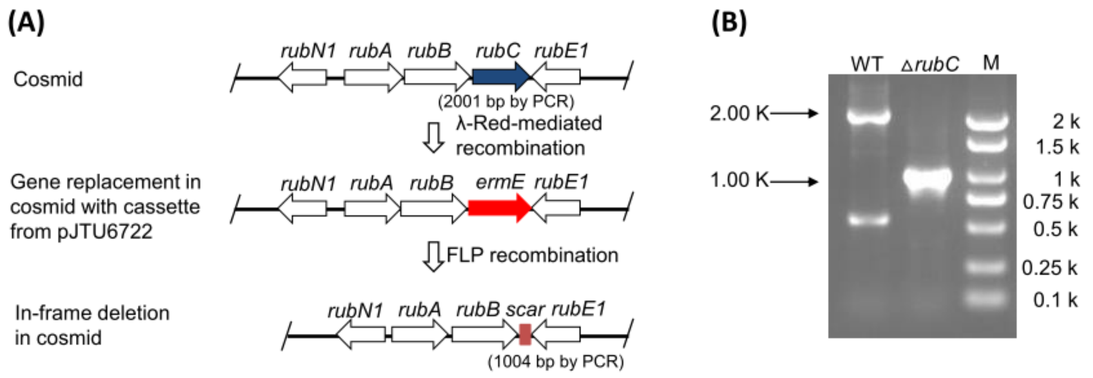

Supplementary Figure 26. Gene replacement of *rubC* using the PCR-targeting method. (A) Scheme for the construction of *rubC* replacement mutant. (B) Confirmation of mutant by PCR using the primers listed in supplementary Table 6. Lane M: DNA molecular ladder. Lane WT, PCR product from the *S. albus* 9B10. Lane Δ*rubC*, PCR product from *S. albus 9B10*-Δ*C* mutant.

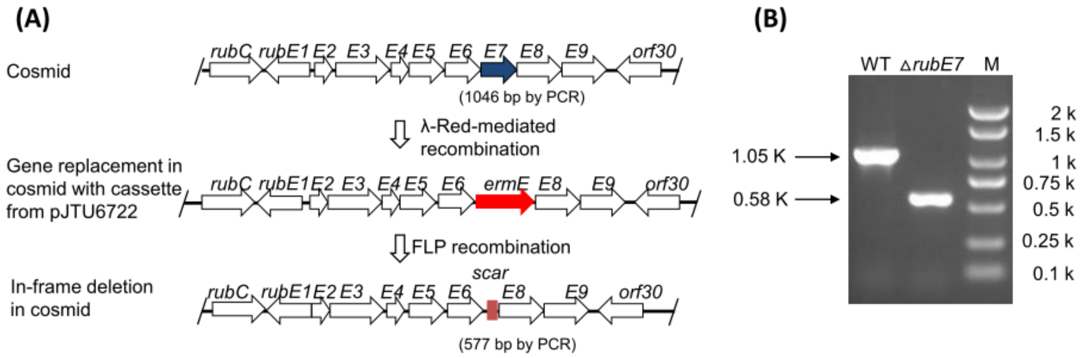

Supplementary Figure 27. Gene replacement of *rubE7* using the PCR-targeting method. (A) Scheme for the construction of *rubE7* replacement mutant. (B) Confirmation of mutant by PCR using the primers listed in supplementary Table 6. Lane M: DNA molecular ladder. Lane WT, PCR product from the *S. albus* 9B10. Lane Δ*rubE7*, PCR product from *S. albus* 9B10-Δ*E7* mutant.

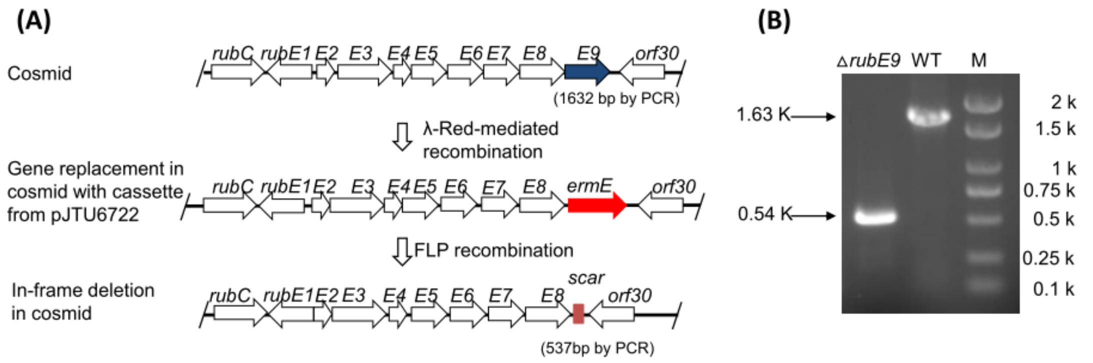

Supplementary Figure 28. Gene replacement of *rubE9* using the PCR-targeting method. (A) Scheme for the construction of *rubE9* replacement mutant. (B) Confirmation of mutant by PCR using the primers listed in supplementary Table 6. Lane M: DNA molecular ladder. Lane WT, PCR product from the *S. albus* 9B10. Lane Δ*rubE9*, PCR product from *S. albus* 9B10-Δ*E9* mutant.

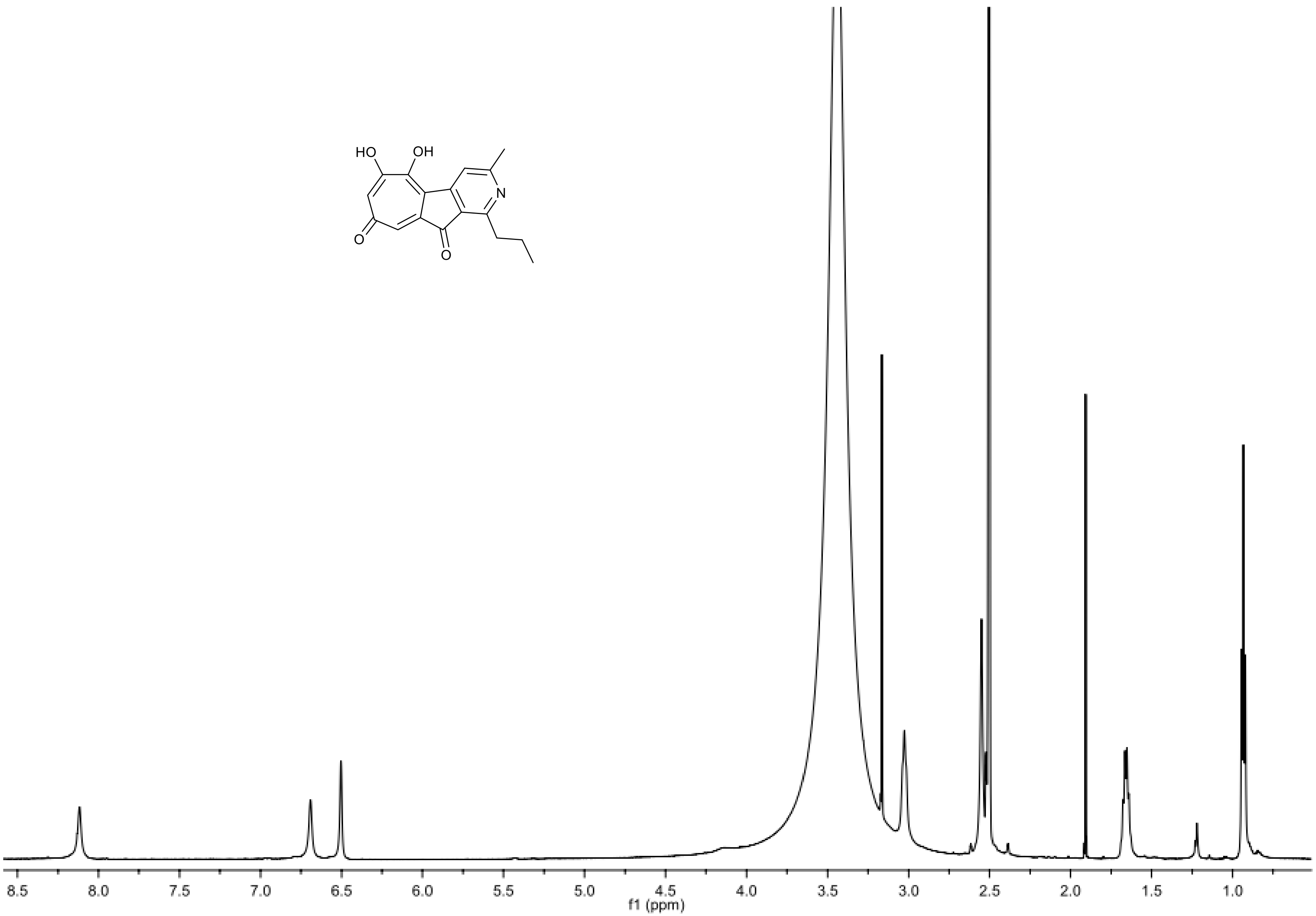

Supplementary Figure 29. ^{1}H NMR spectrum of compound **3** in DMSO-d_6.

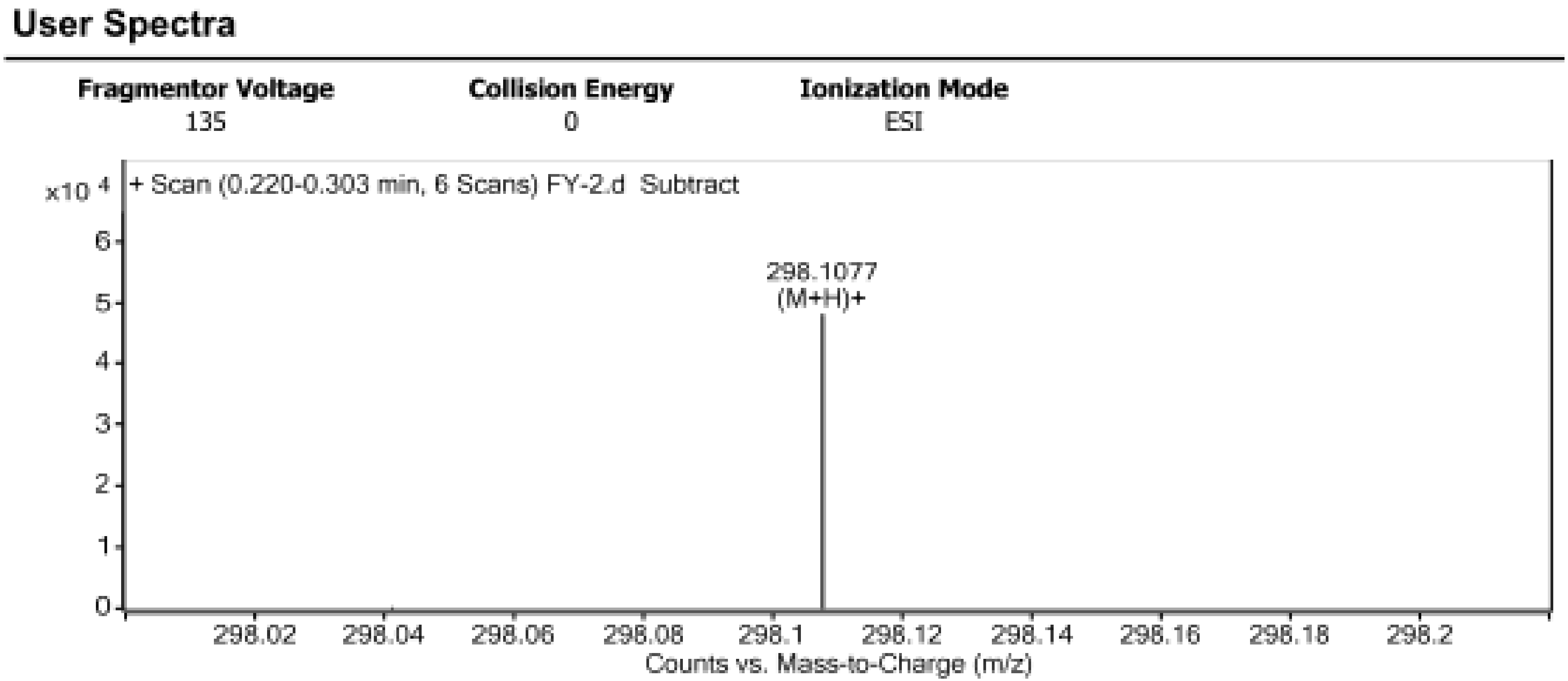

Supplementary Figure 30. HRESIMS analysis of compound **3**.

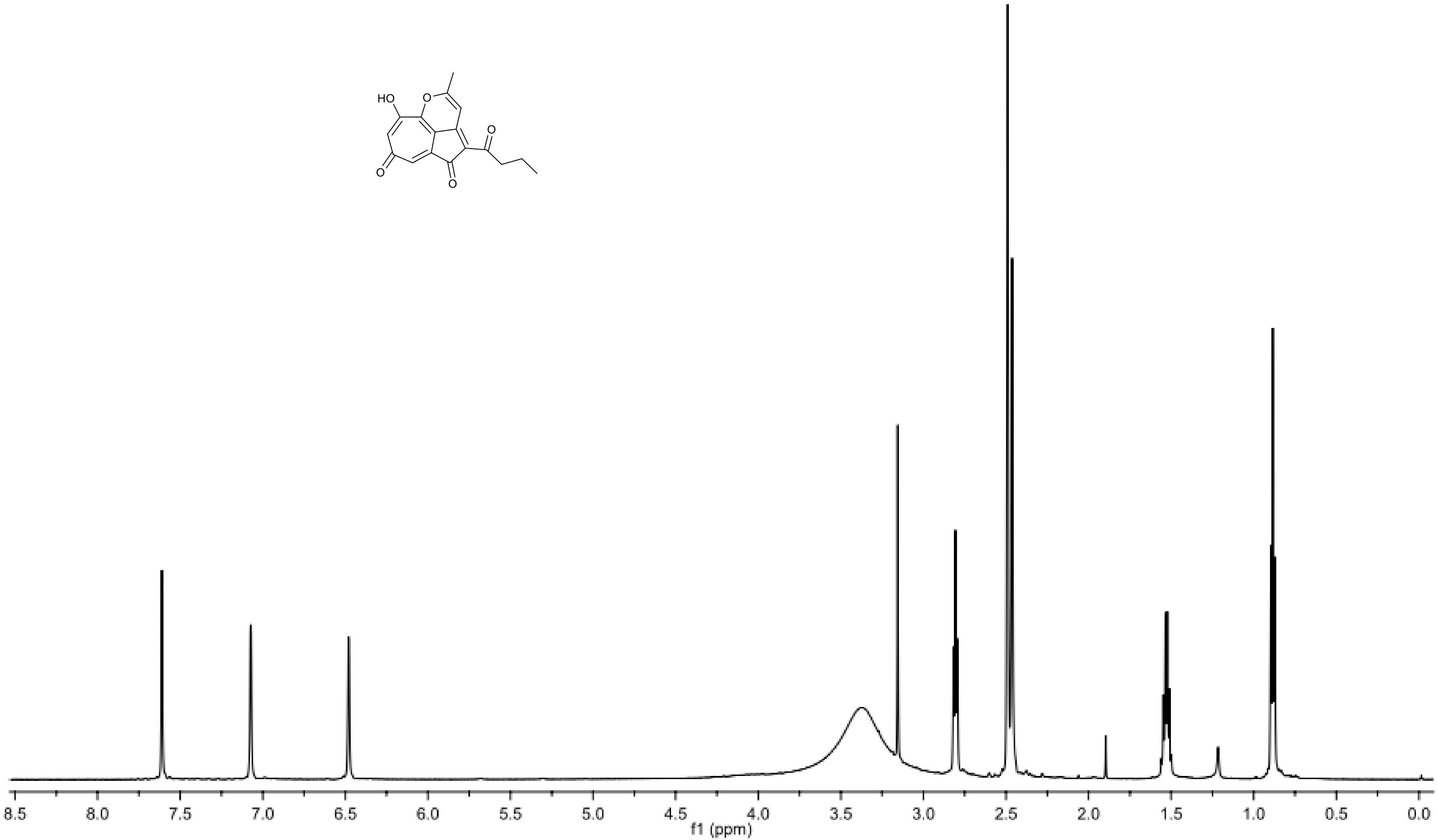

Supplementary Figure 31. ^{1}H NMR spectrum of compound **4** in DMSO-d_6.

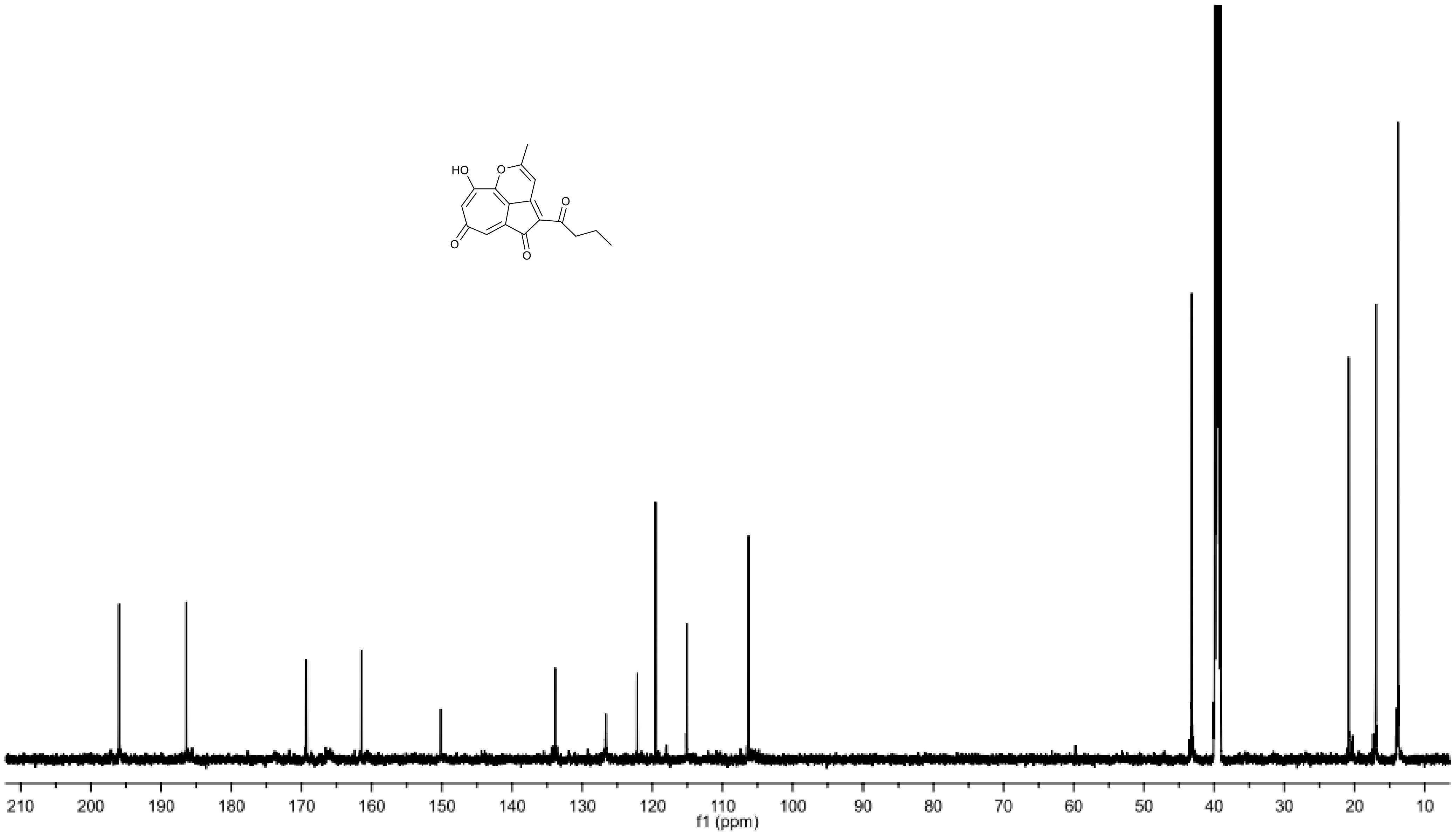

Supplementary Figure 32. ^{13}C NMR spectrum of compound **4** in DMSO-d_6.

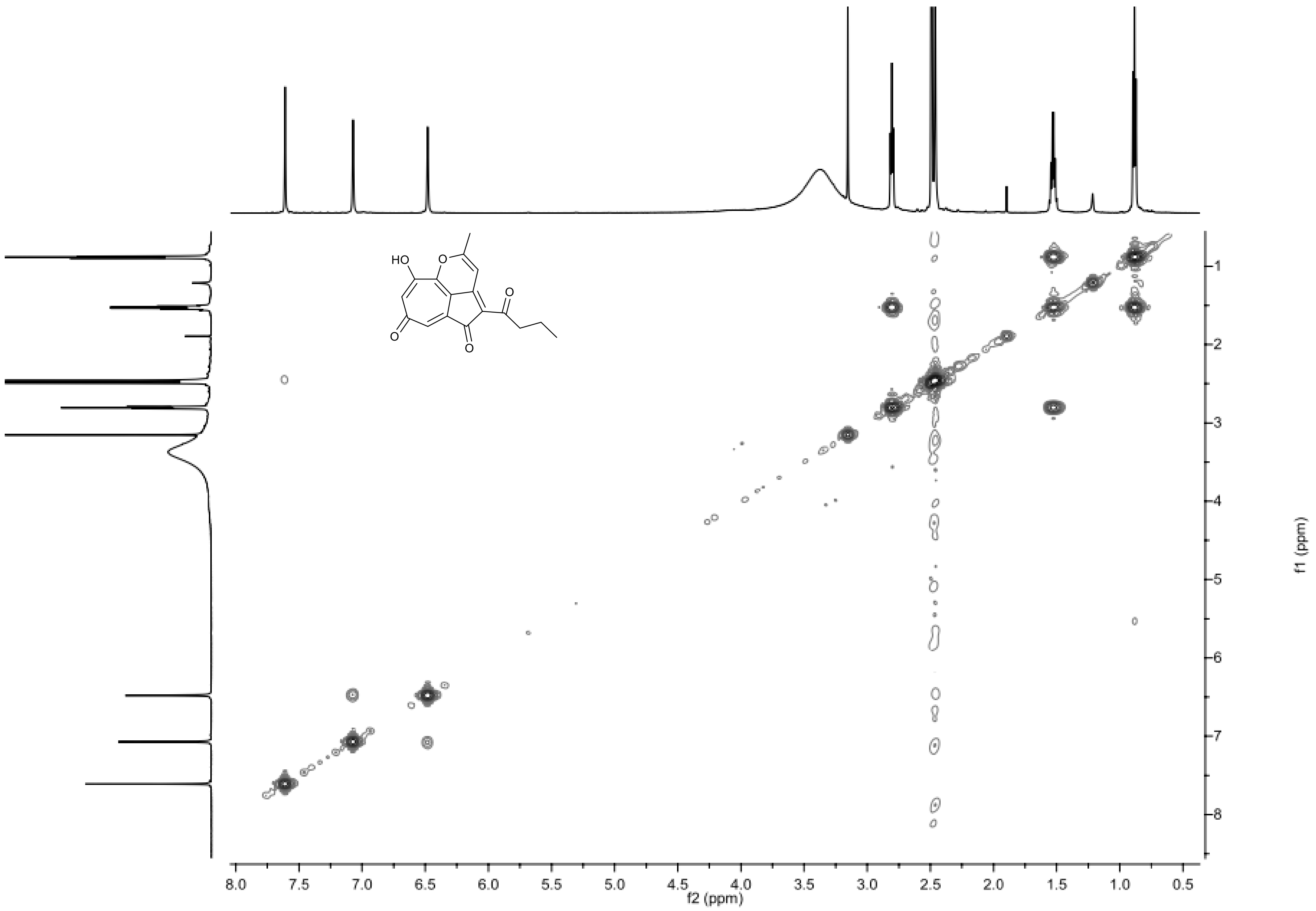

Supplementary Figure 33. ^1H-^1H COSY NMR spectrum of compound **4** in DMSO-d_6.

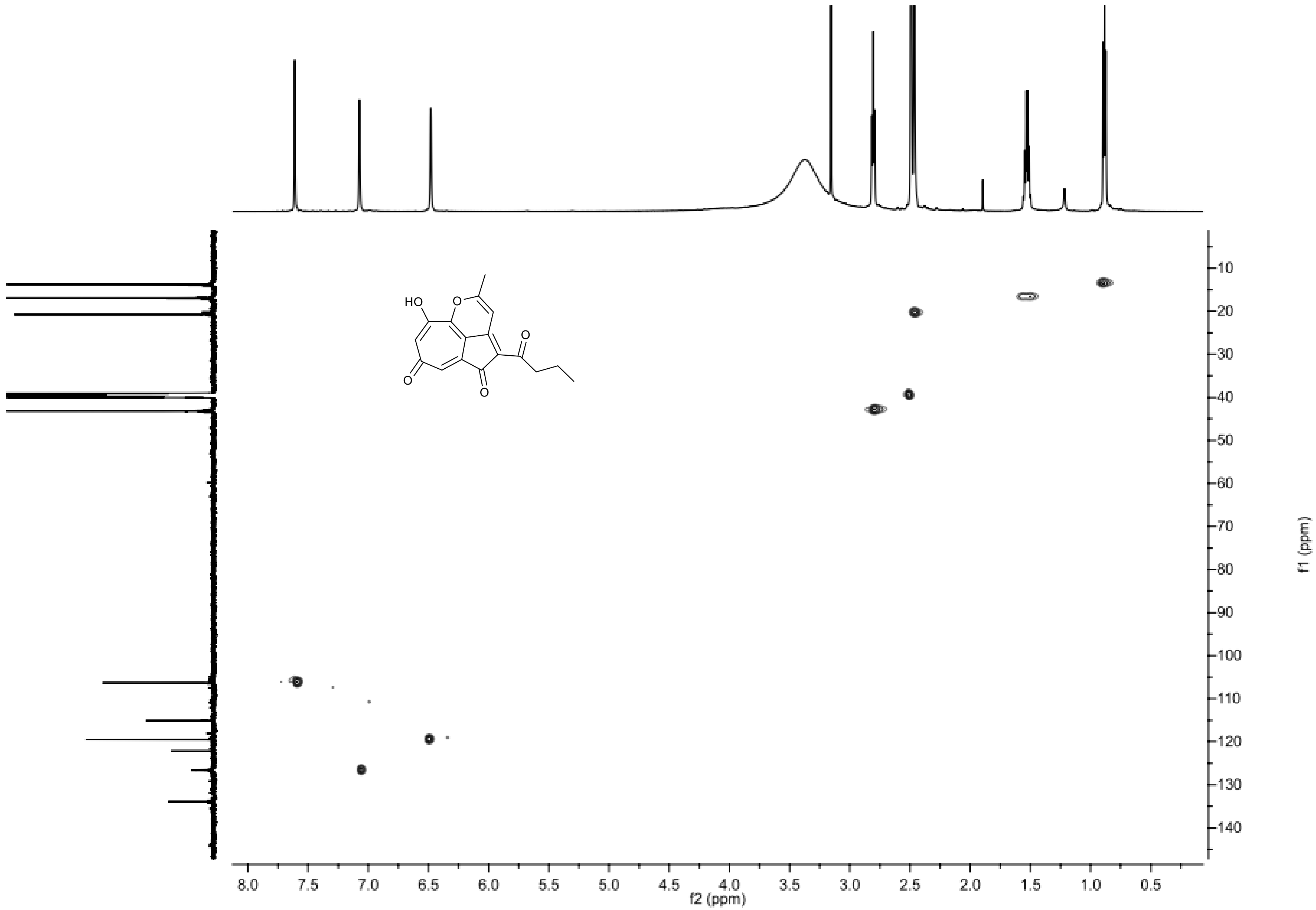

Supplementary Figure 34. HSQC NMR spectrum of compound **4** in DMSO-d_6.

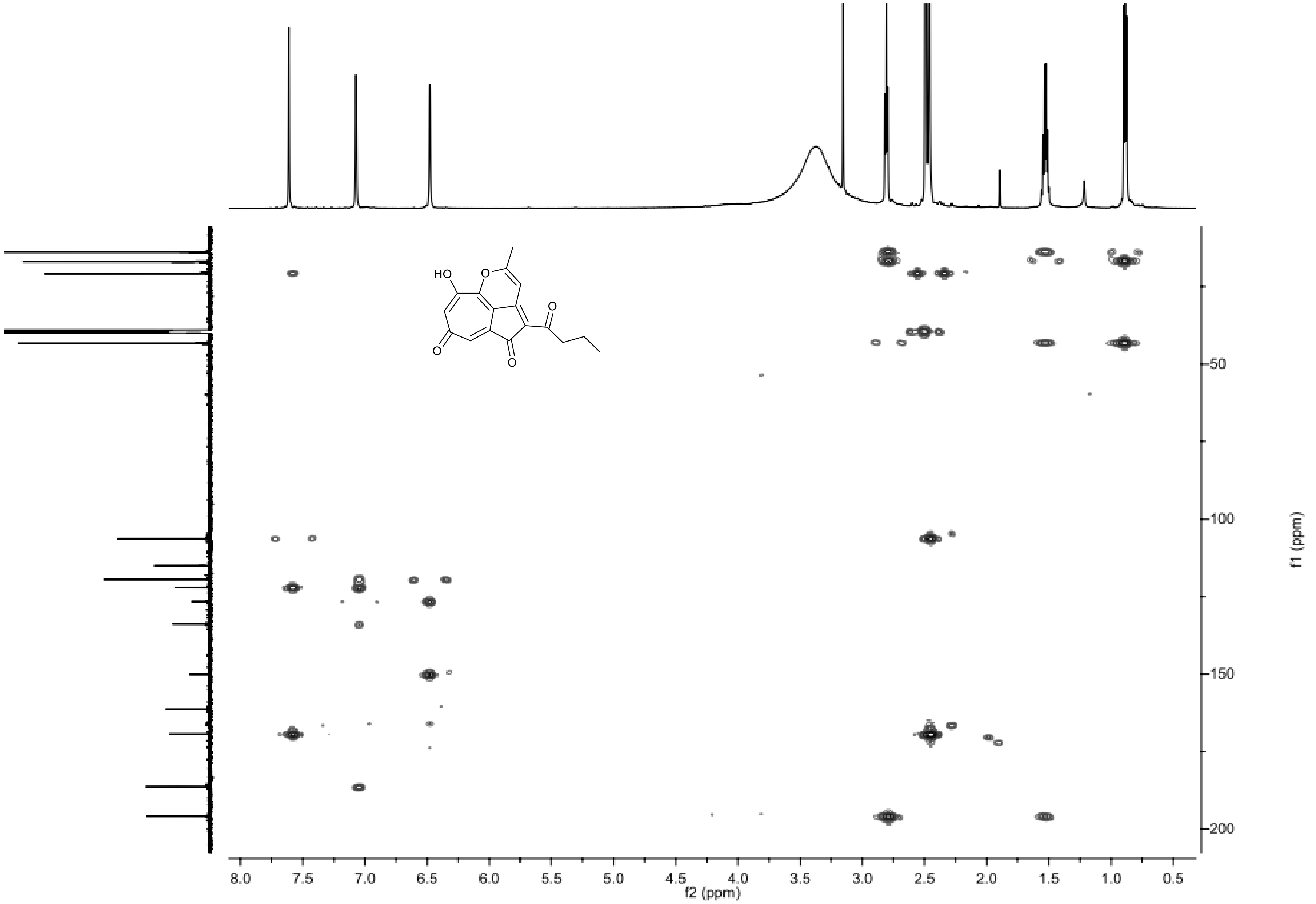

Supplementary Figure 35. HMBC NMR spectrum of compound **4** in DMSO-d_6.

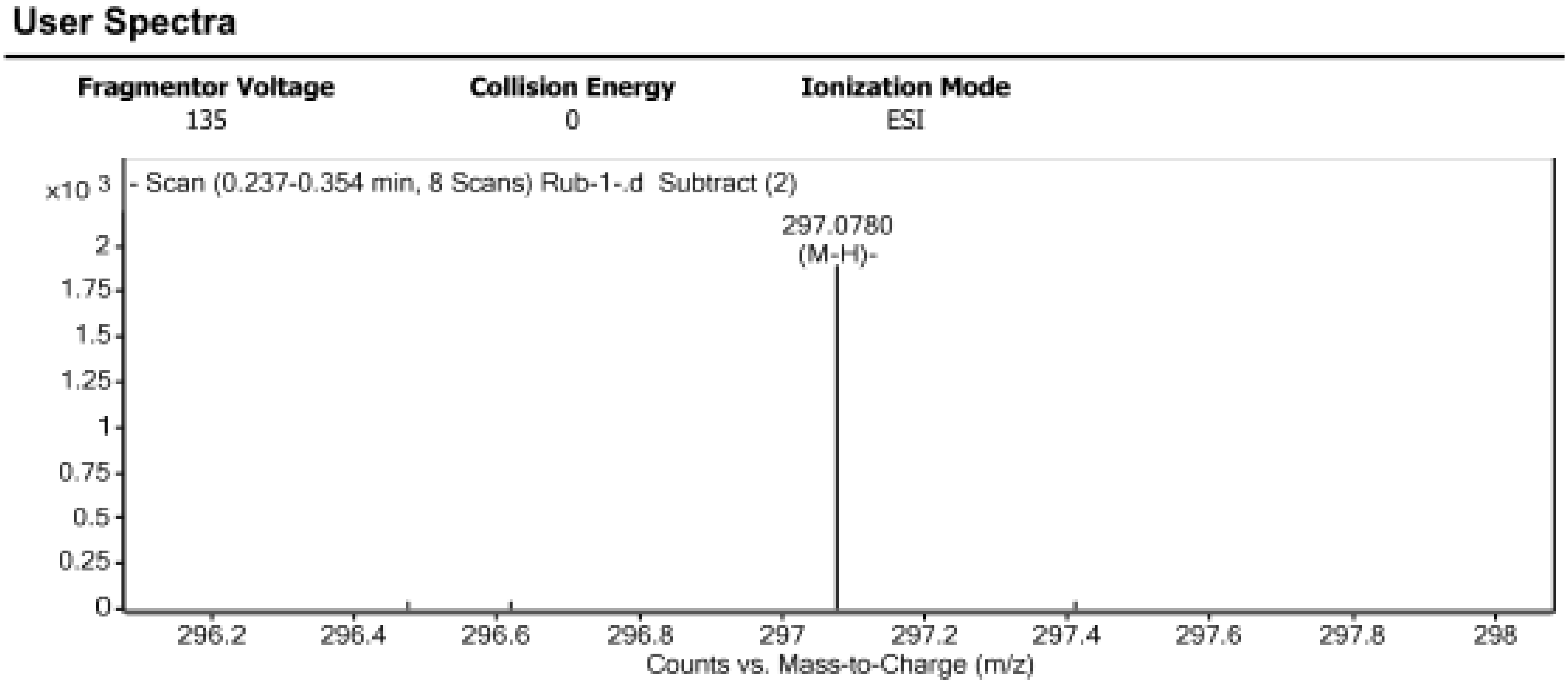

Supplementary Figure 36. HRESIMS analysis of compound **4**.

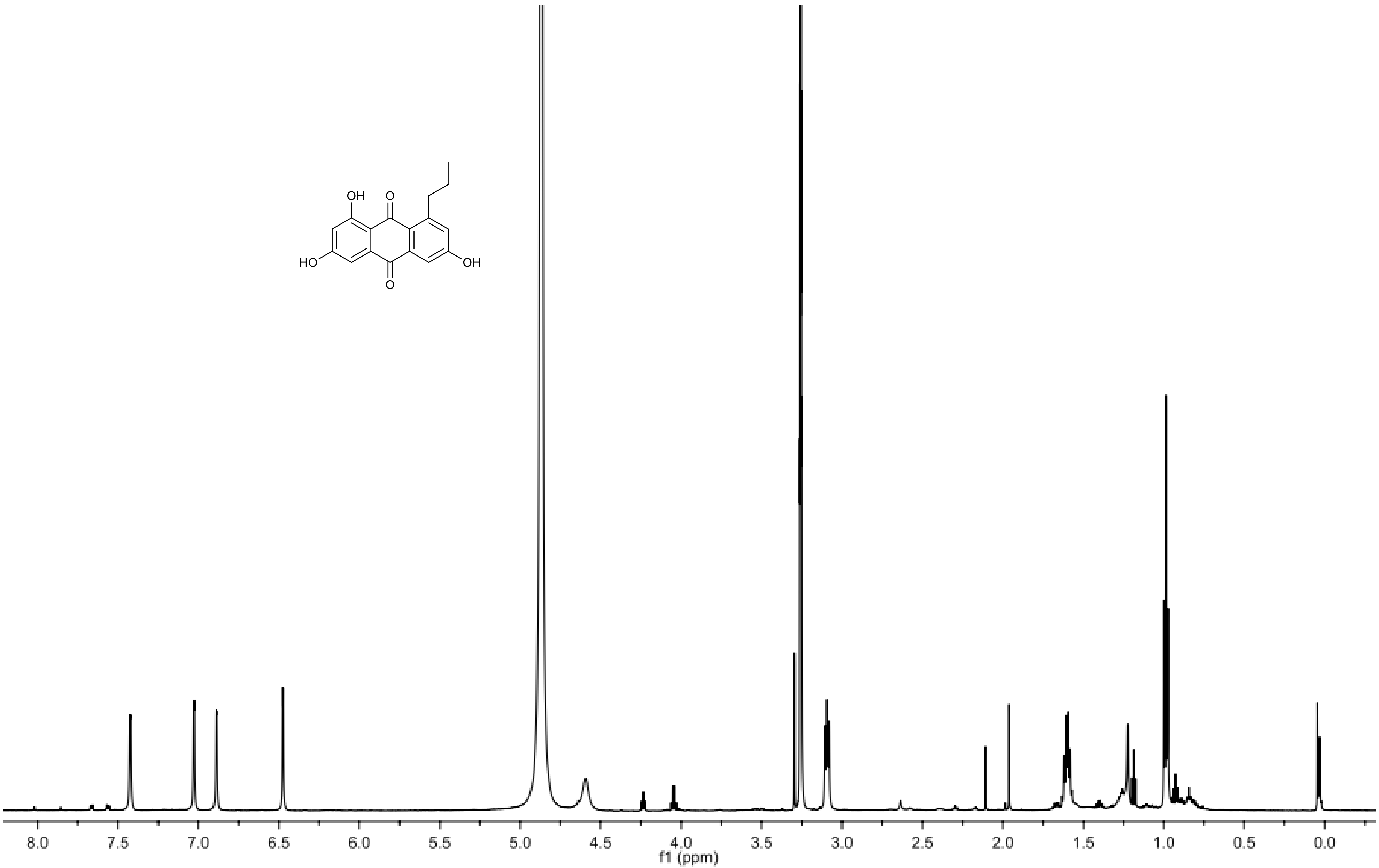

Supplementary Figure 37. ^{1}H NMR spectrum of compound **5** in CD_3OD.

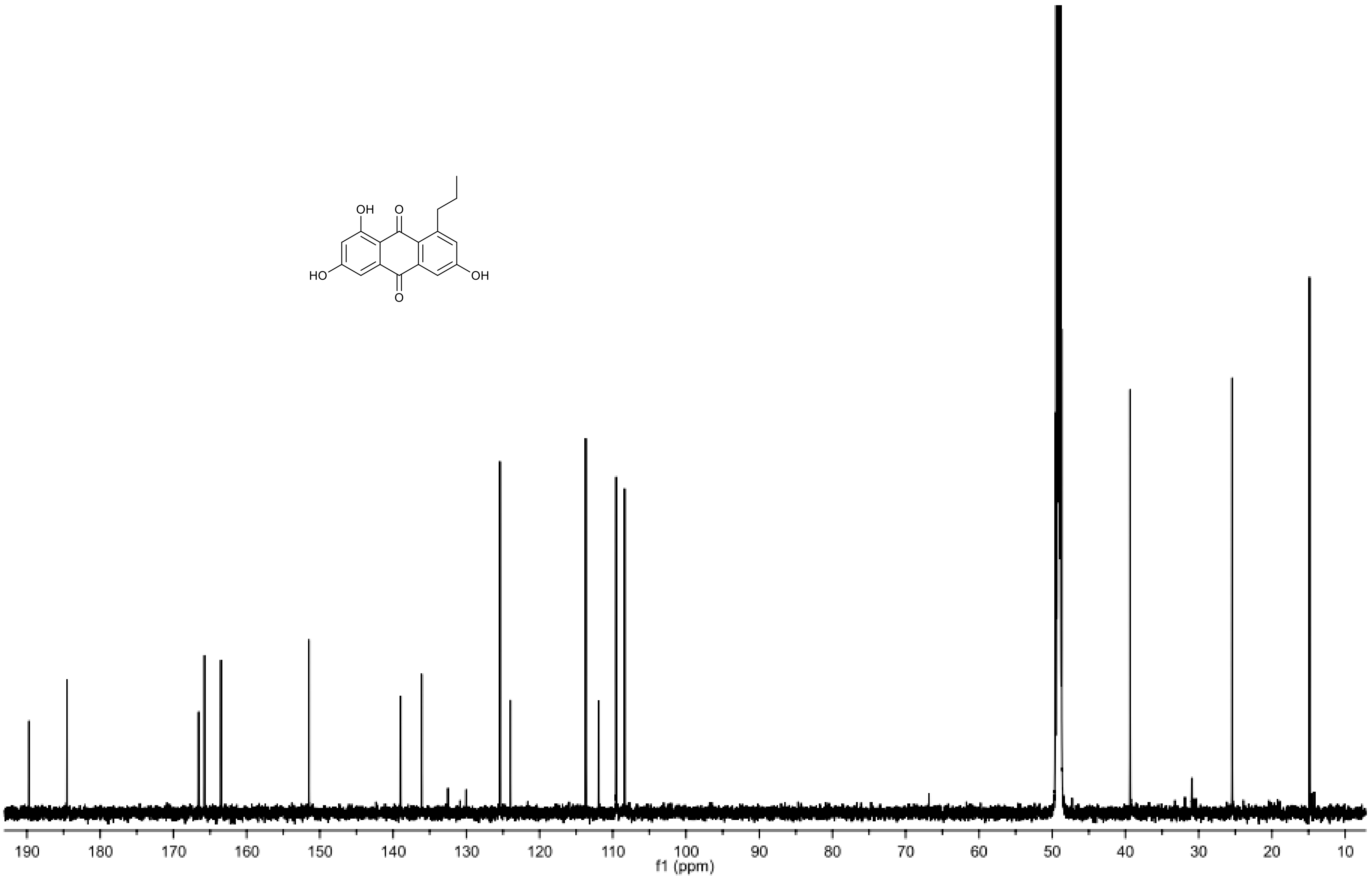

Supplementary Figure 38. ^{13}C NMR spectrum of compound **5** in CD_3OD.

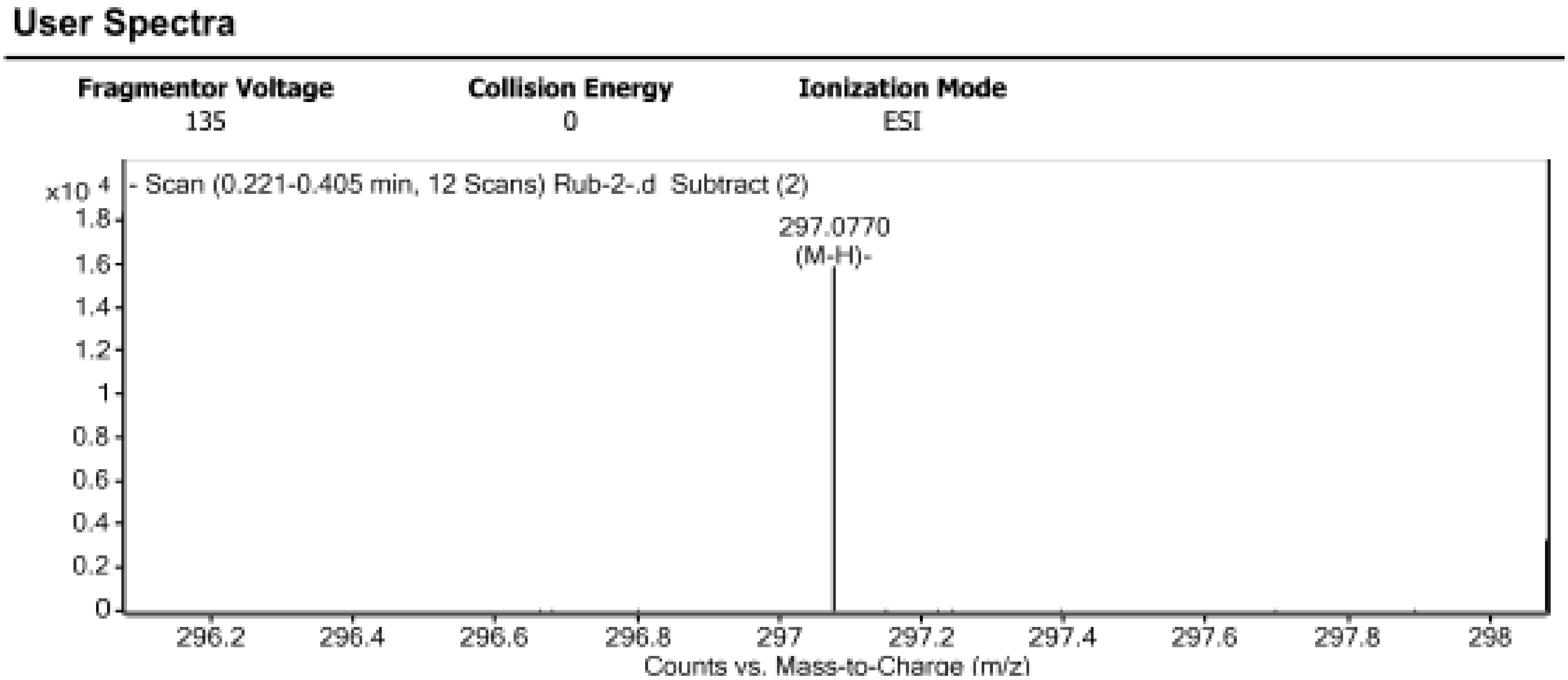

Supplementary Figure 39. HRESIMS analysis of compound **5**.

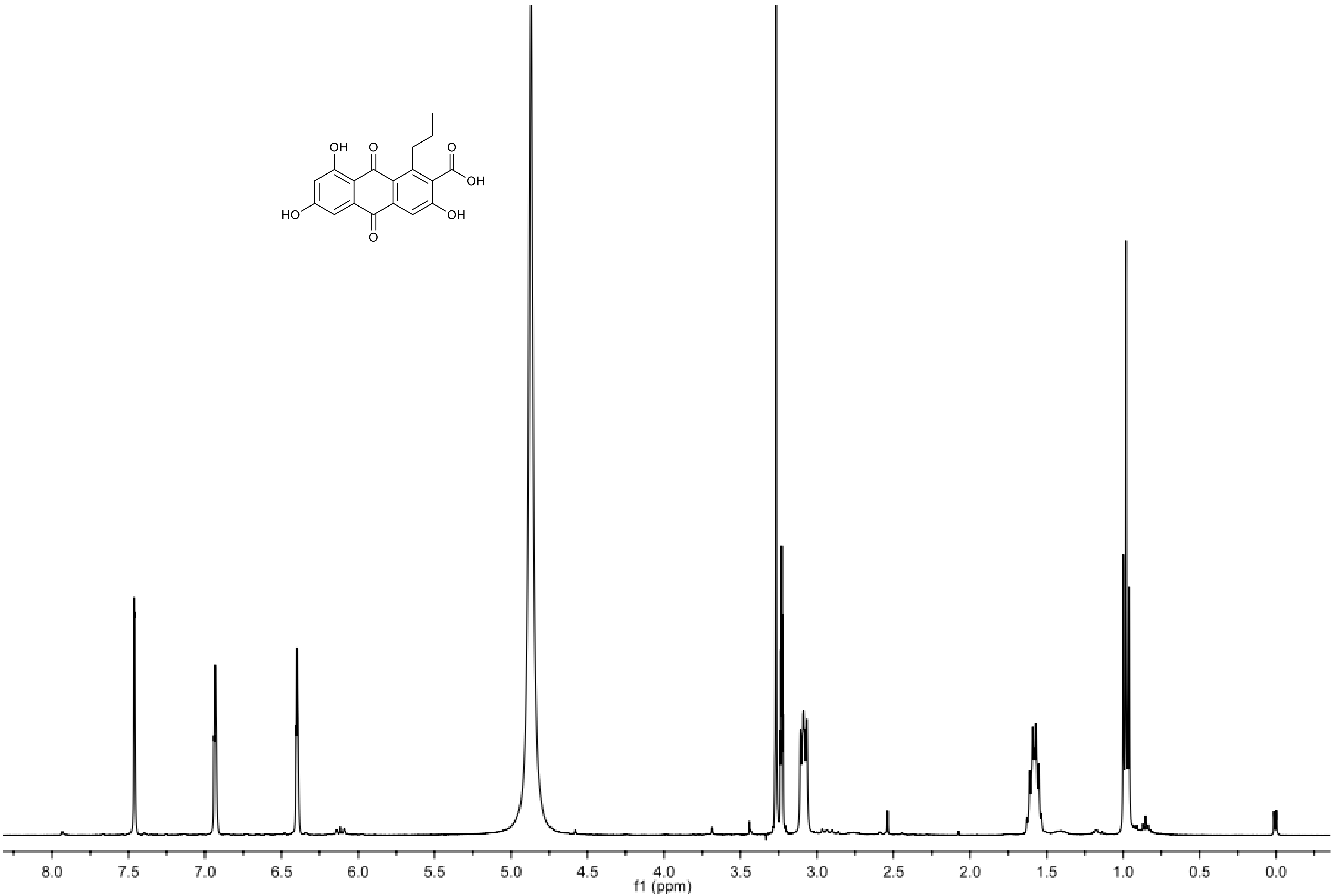

Supplementary Figure 40. ^{1}H NMR spectrum of compound **6** in CD_3OD.

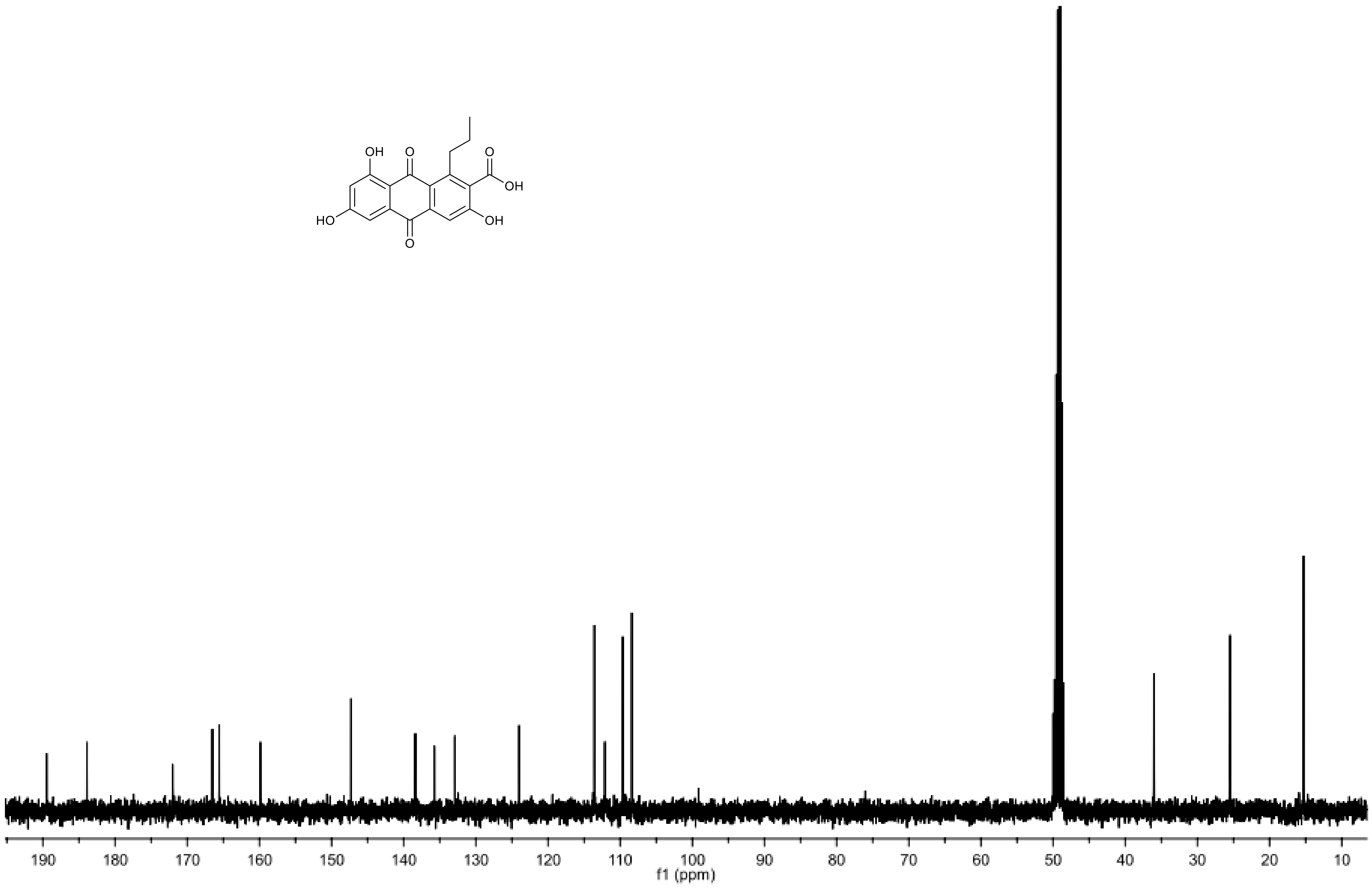

Supplementary Figure 41. ^{13}C NMR spectrum of compound **6** in CD_3OD.

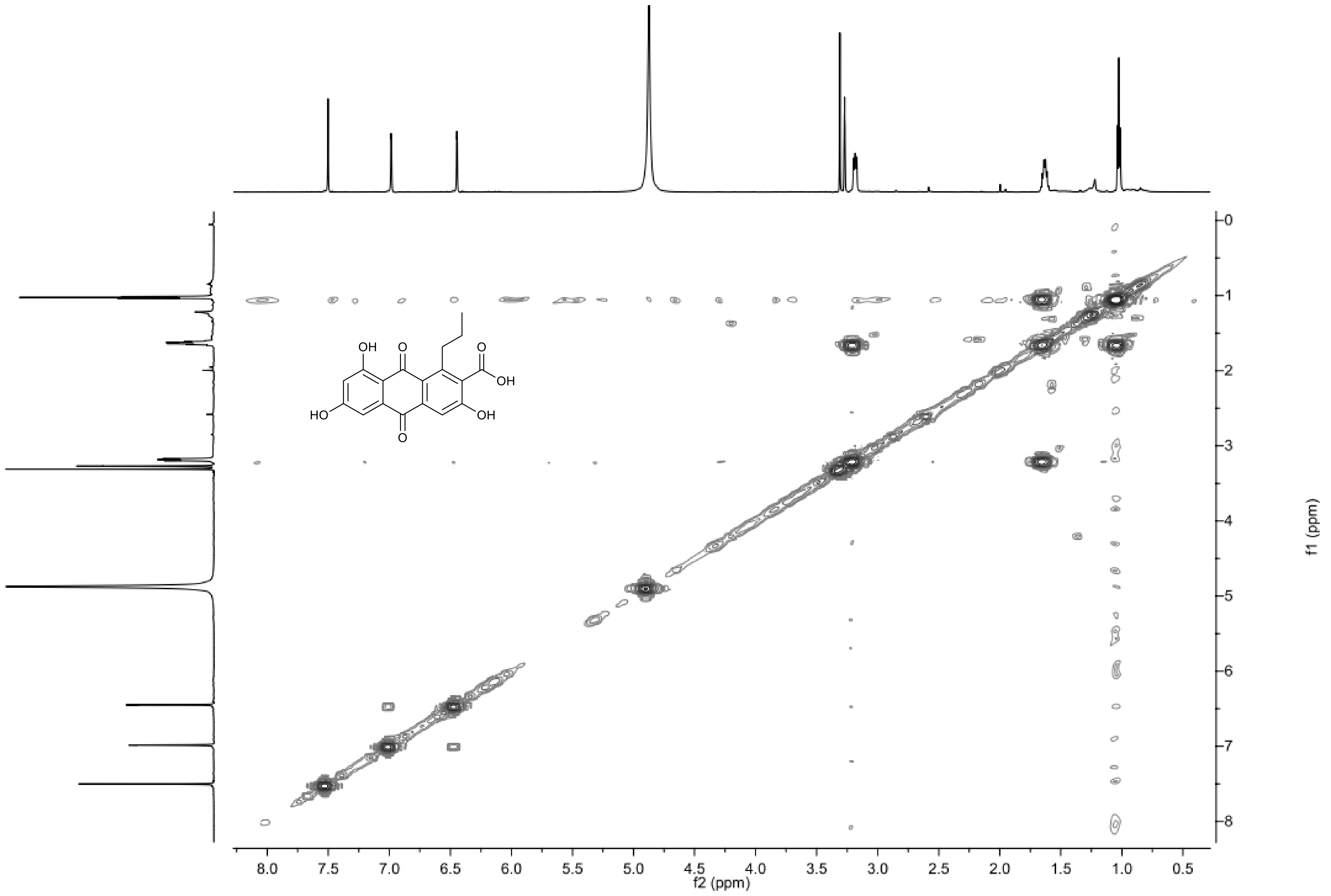

Supplementary Figure 42. ^1H-^1H COSY NMR spectrum of compound **6** in CD_3OD.

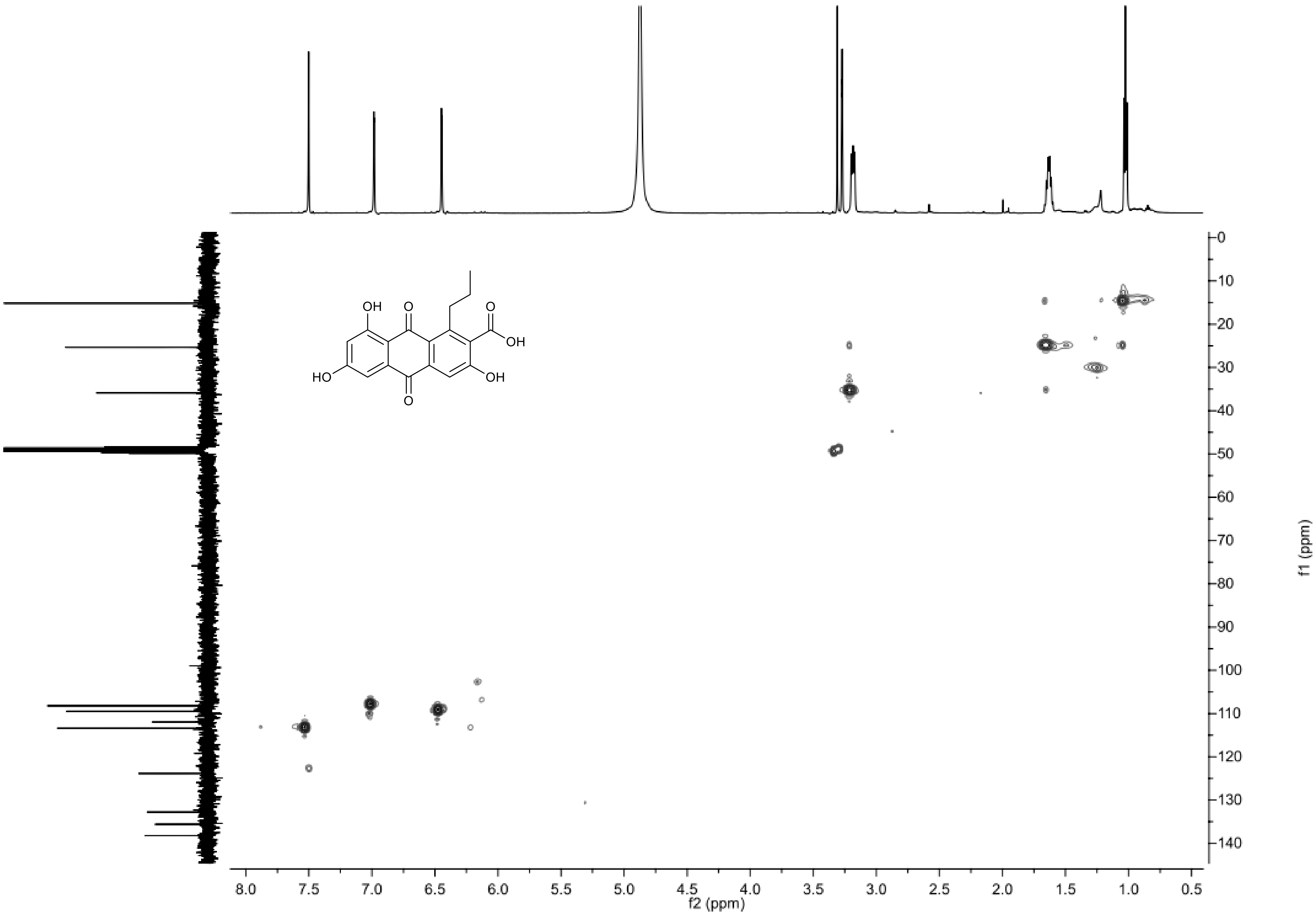

Supplementary Figure 43. HSQC NMR spectrum of compound **6** in CD_3OD.

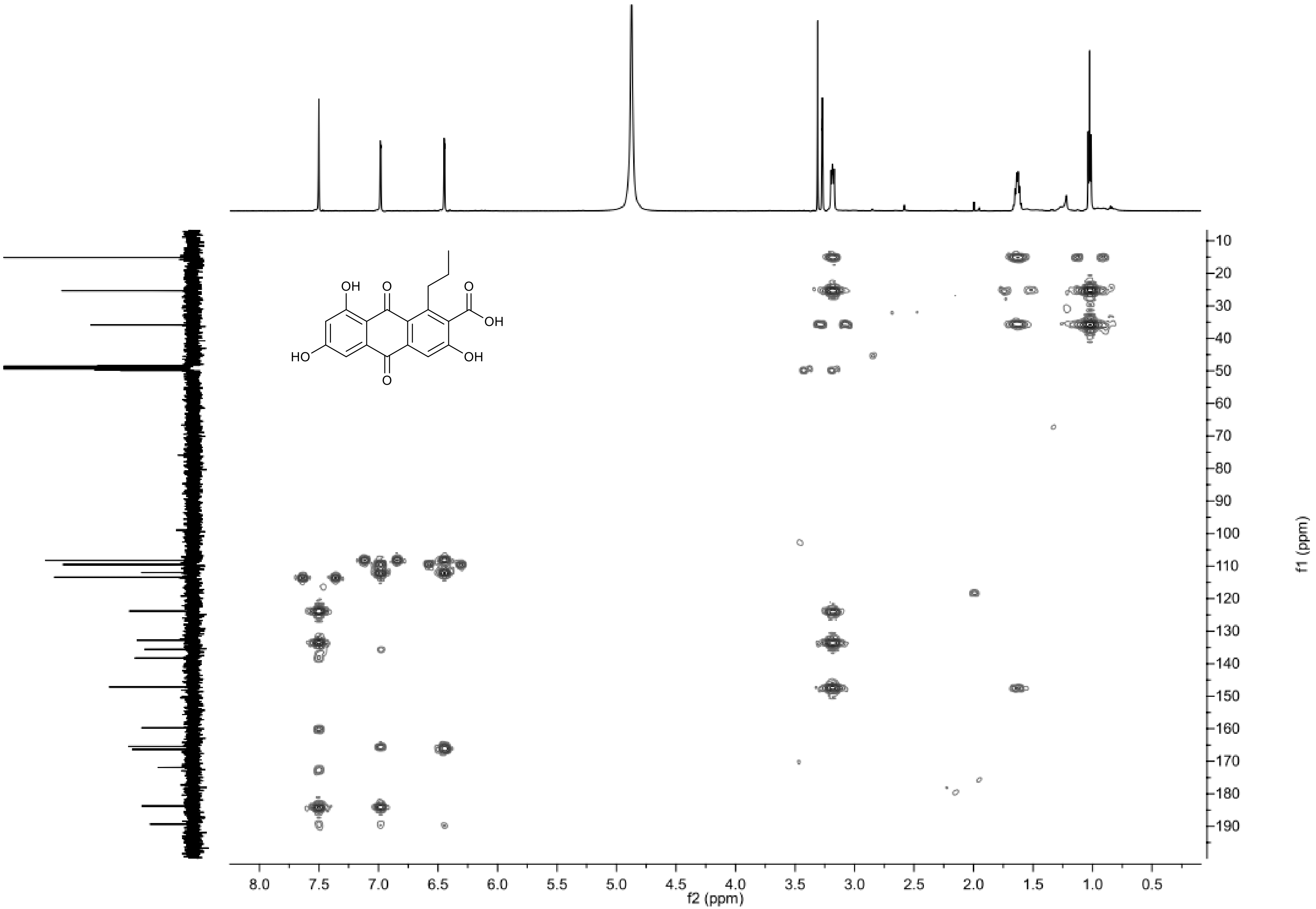

Supplementary Figure 44. HMBC NMR spectrum of compound **6** in CD_3OD.

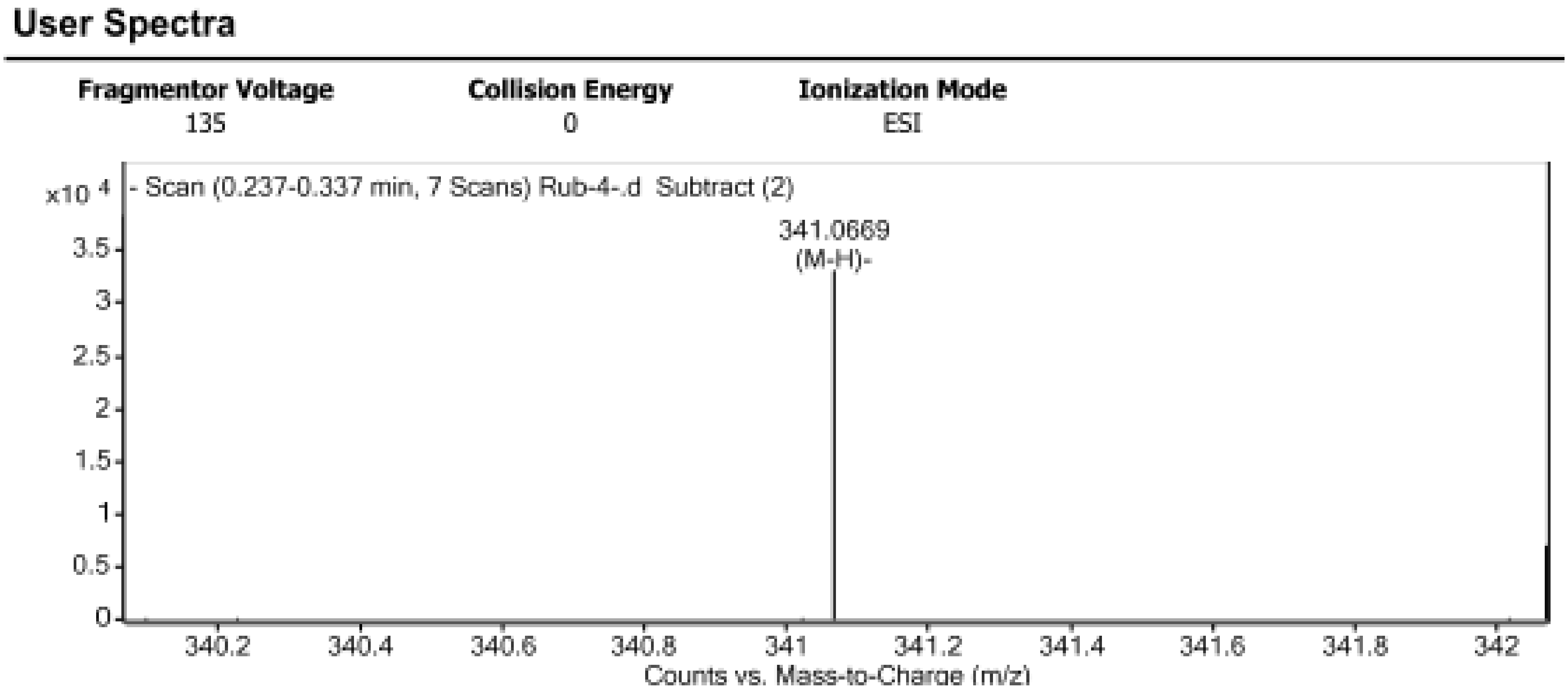

Supplementary Figure 45. HRESIMS analysis of compound **6**.

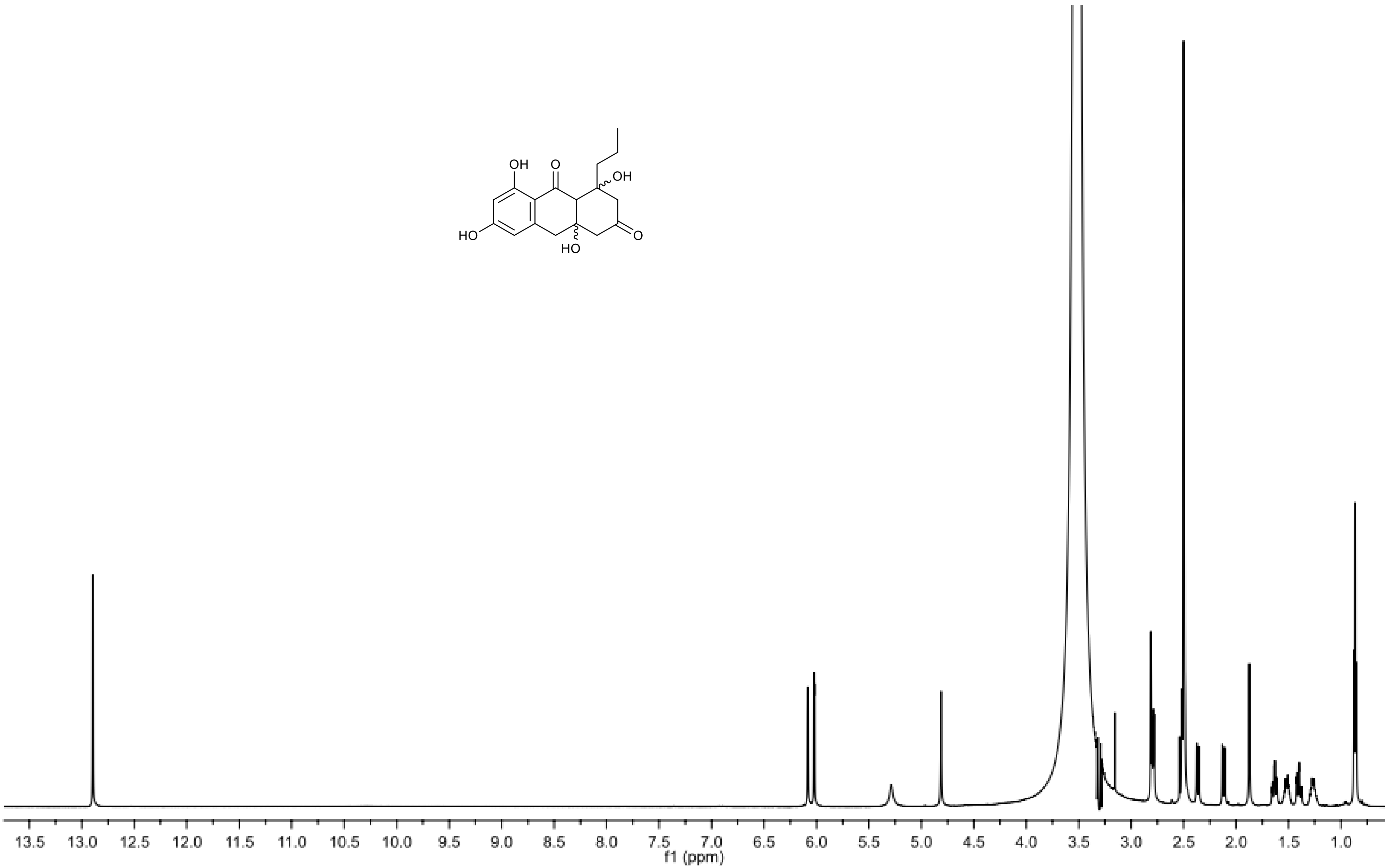

Supplementary Figure 46. ^{1}H NMR spectrum of compound **7** in DMSO-d_6.

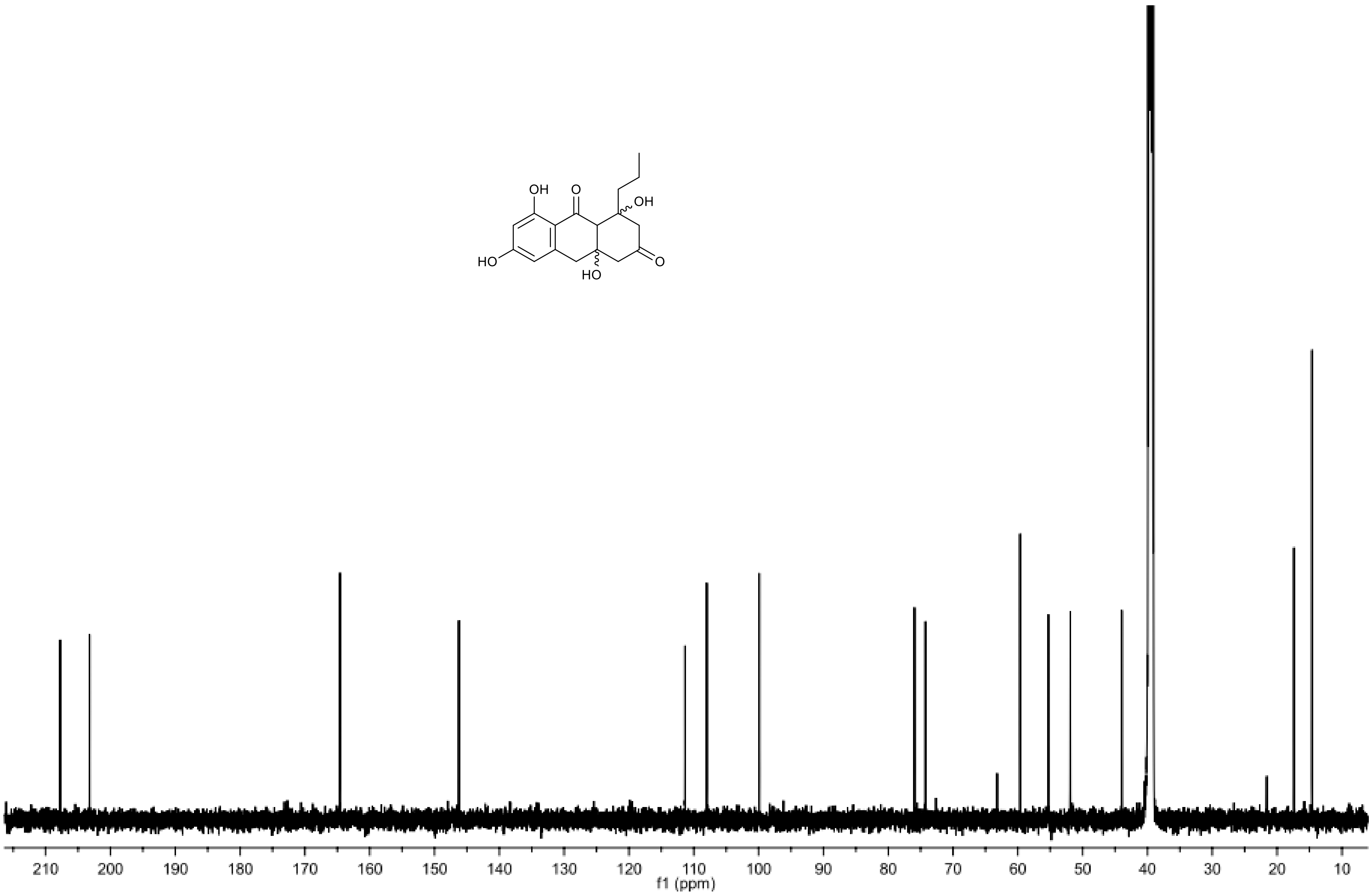

Supplementary Figure 47. ^{13}C NMR spectrum of compound **7** in DMSO-d_6.

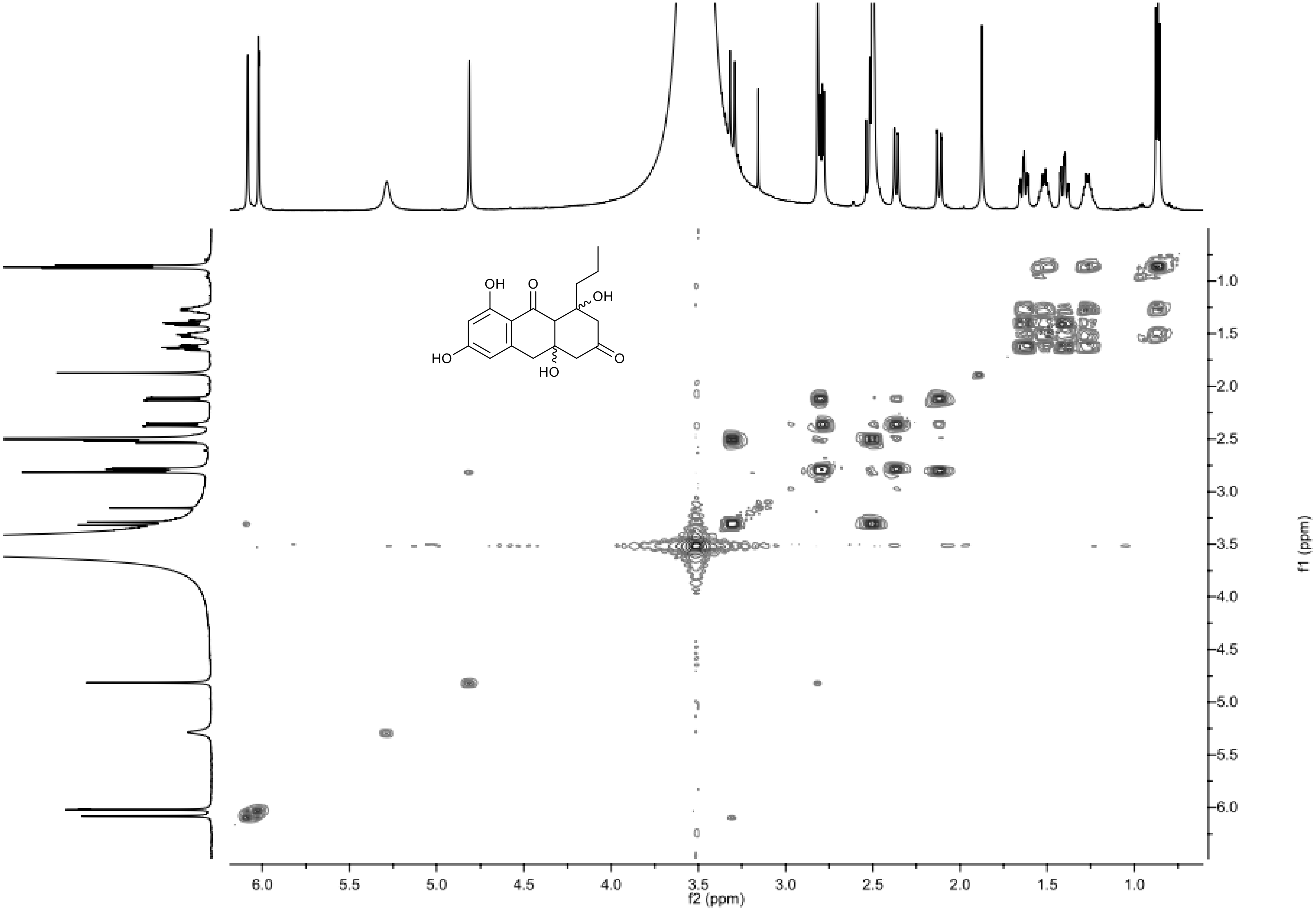

Supplementary Figure 48. ^1H-^1H COSY NMR spectrum of compound **7** in DMSO-d_6.

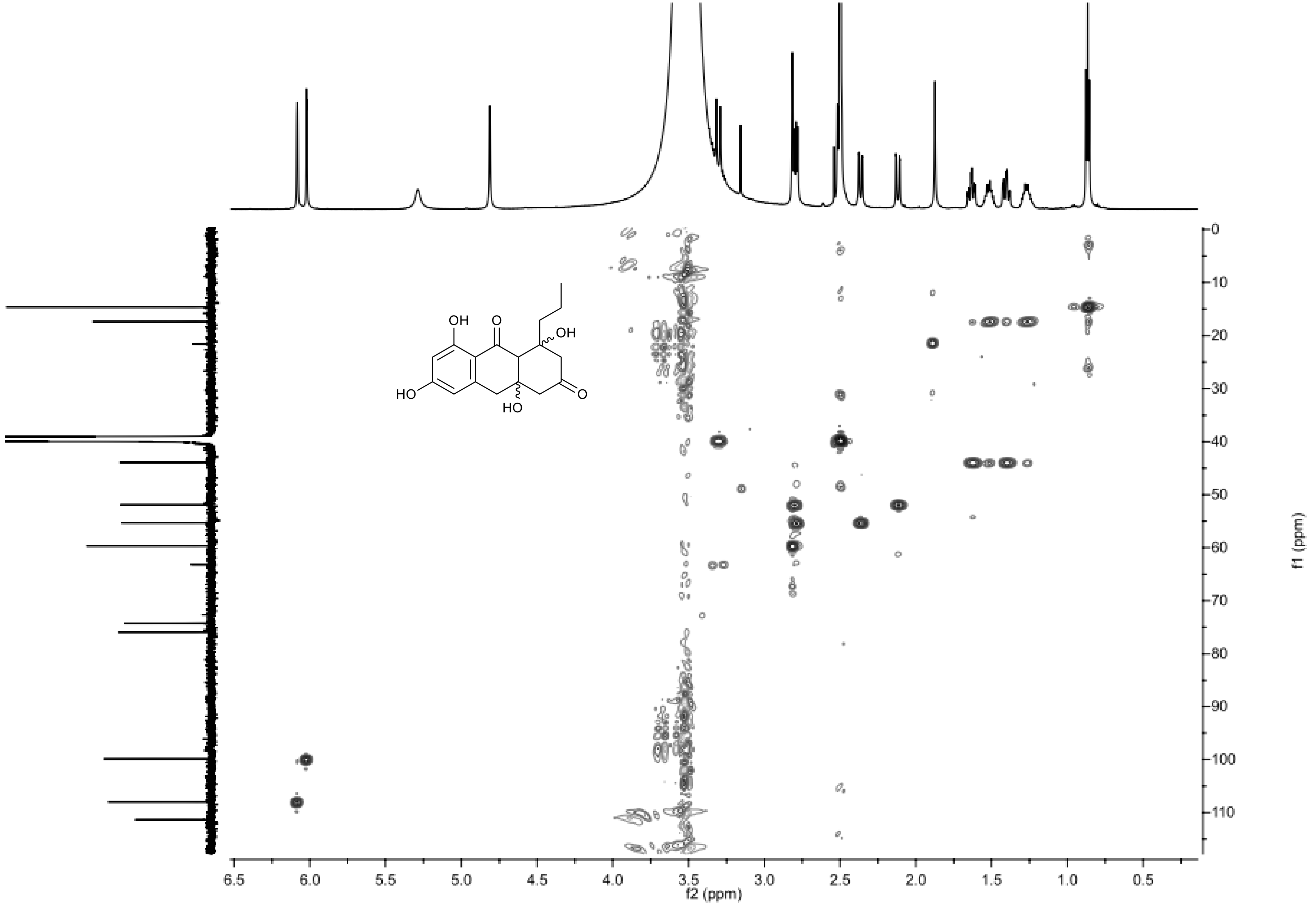

Supplementary Figure 49. HSQC NMR spectrum of compound **7** in DMSO-d_6.

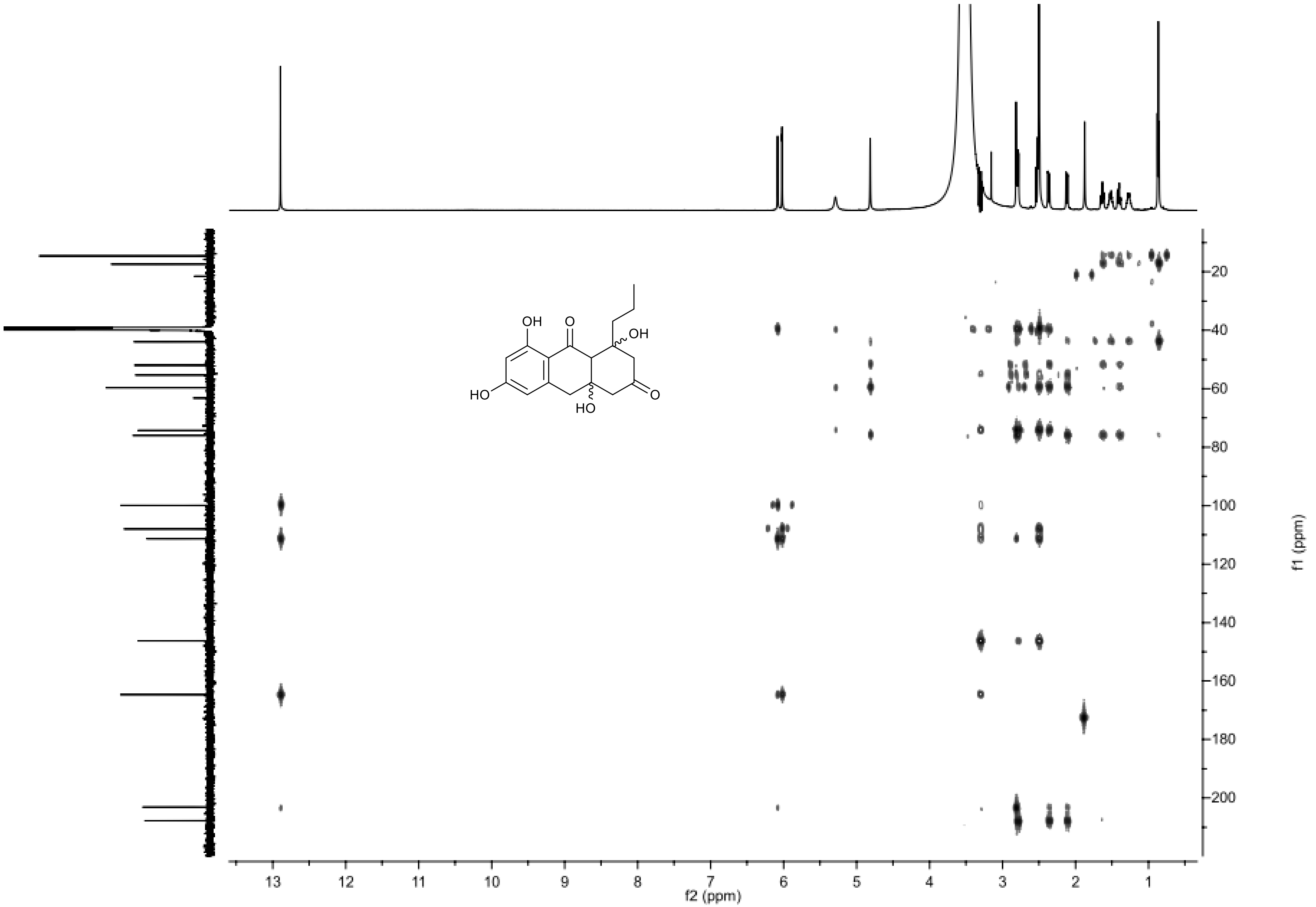

Supplementary Figure 50. HMBC NMR spectrum of compound **7** in DMSO-d_6.

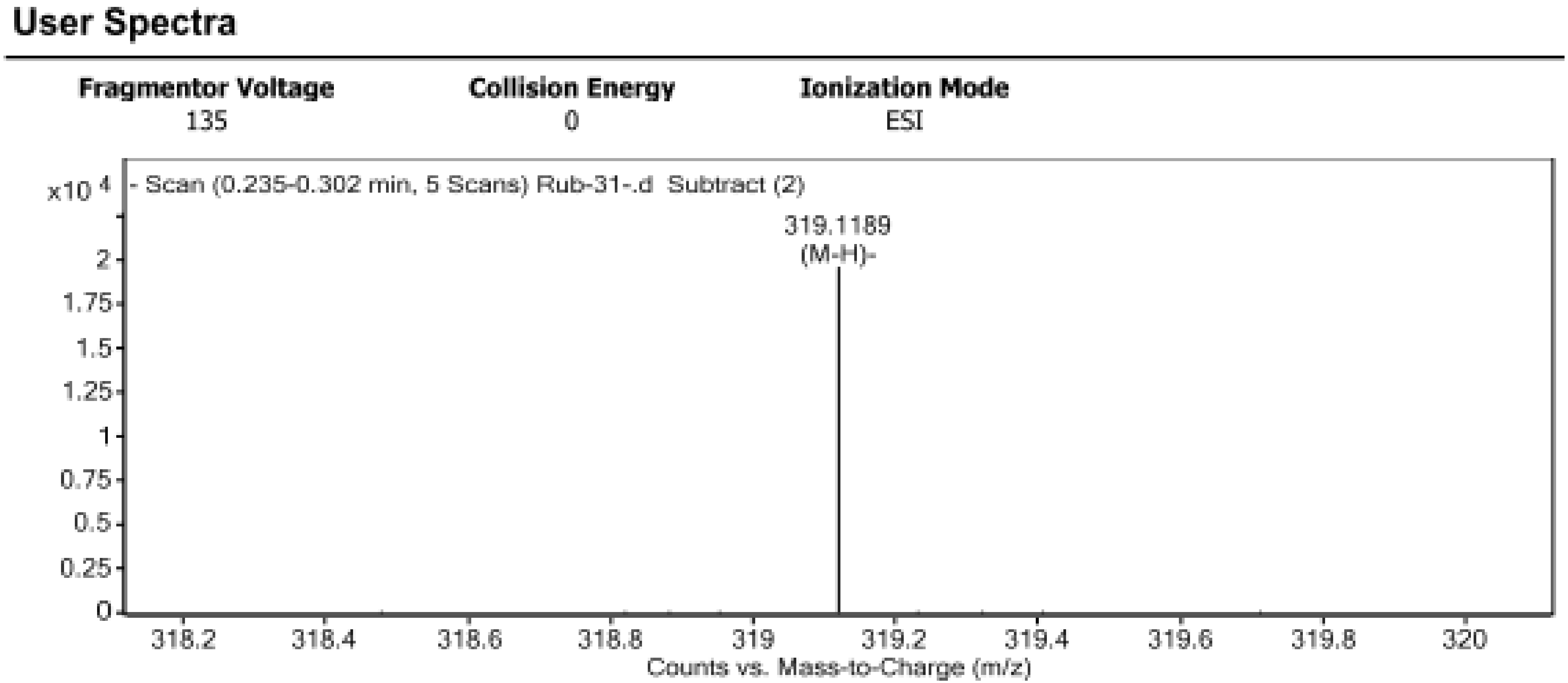

Supplementary Figure 51. HRESIMS analysis of compound **7**.

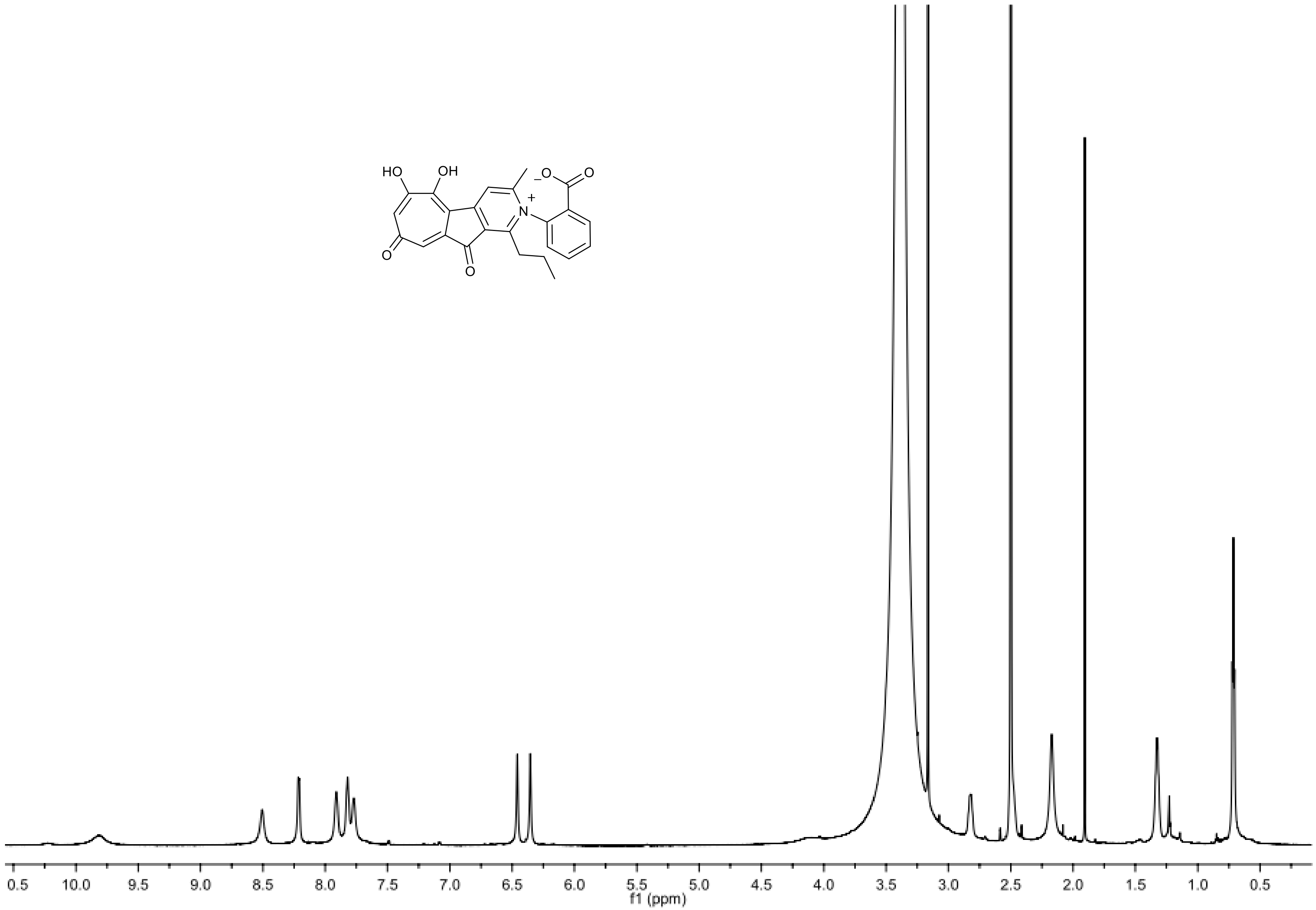

Supplementary Figure 52. ^{1}H NMR spectrum of compound **8** in DMSO-d_6.

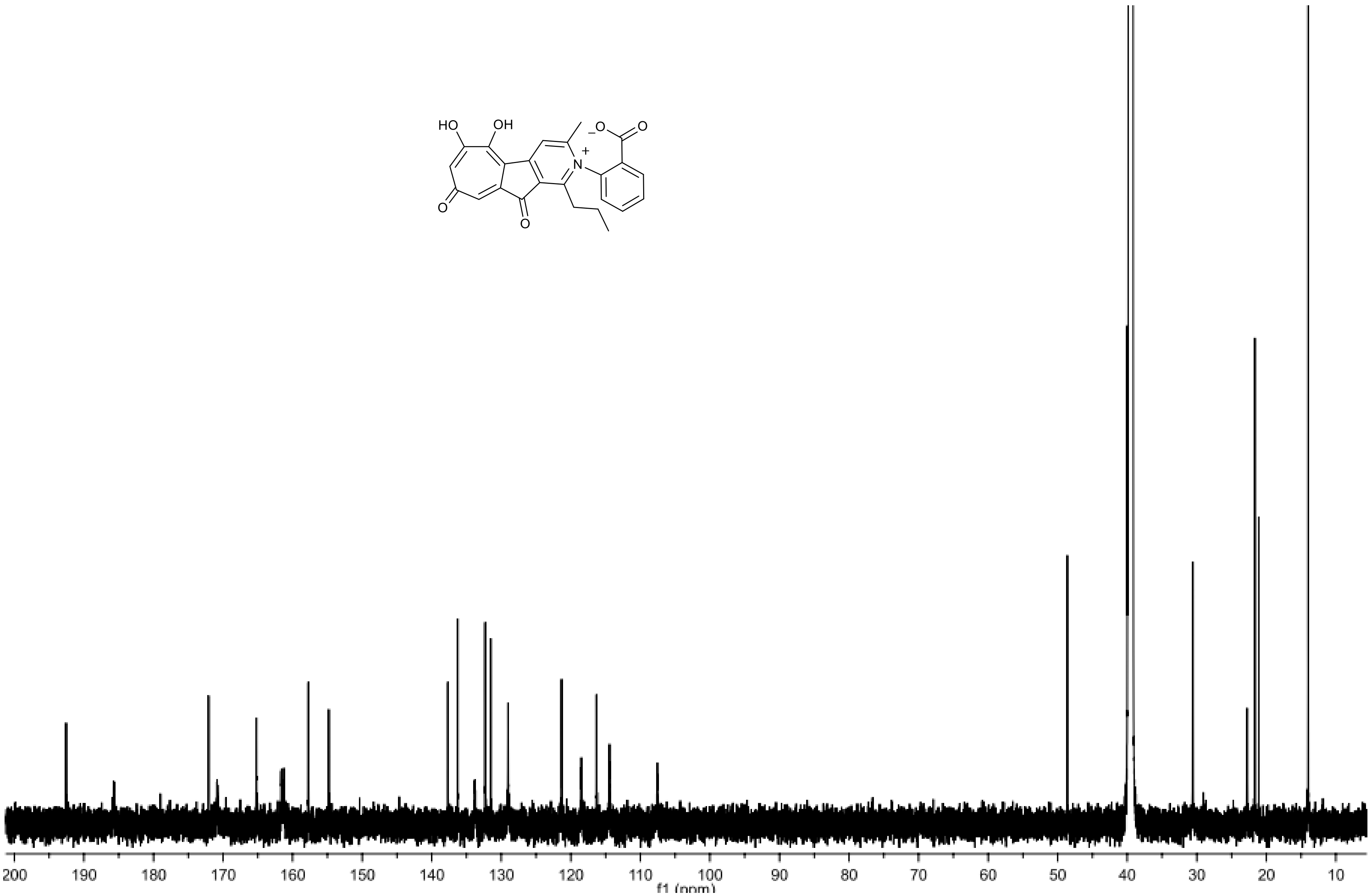

Supplementary Figure 53. ^{13}C NMR spectrum of compound **8** in DMSO-d_6.

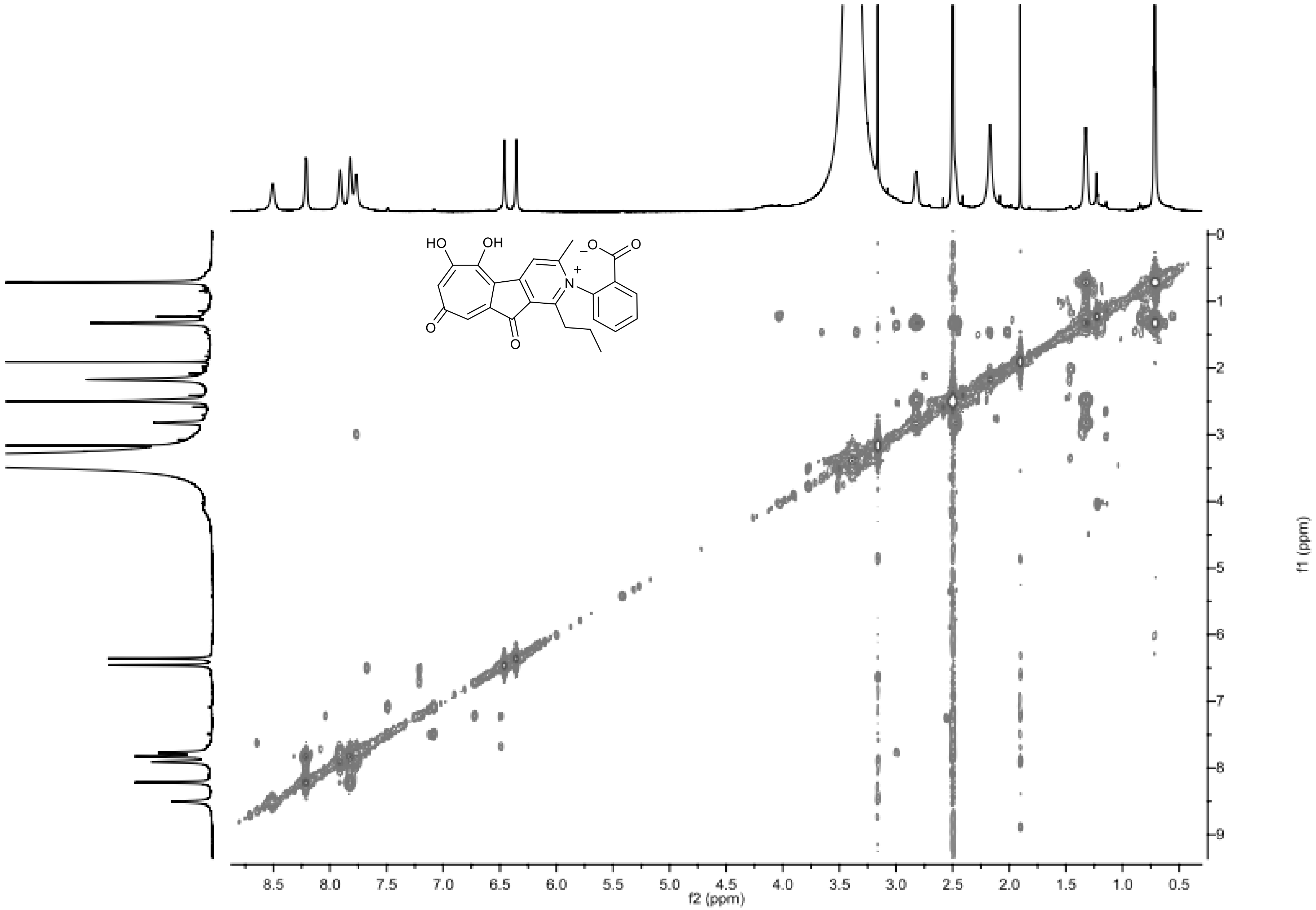

Supplementary Figure 54. ^1H-^1H COSY NMR spectrum of compound **8** in DMSO-d_6.

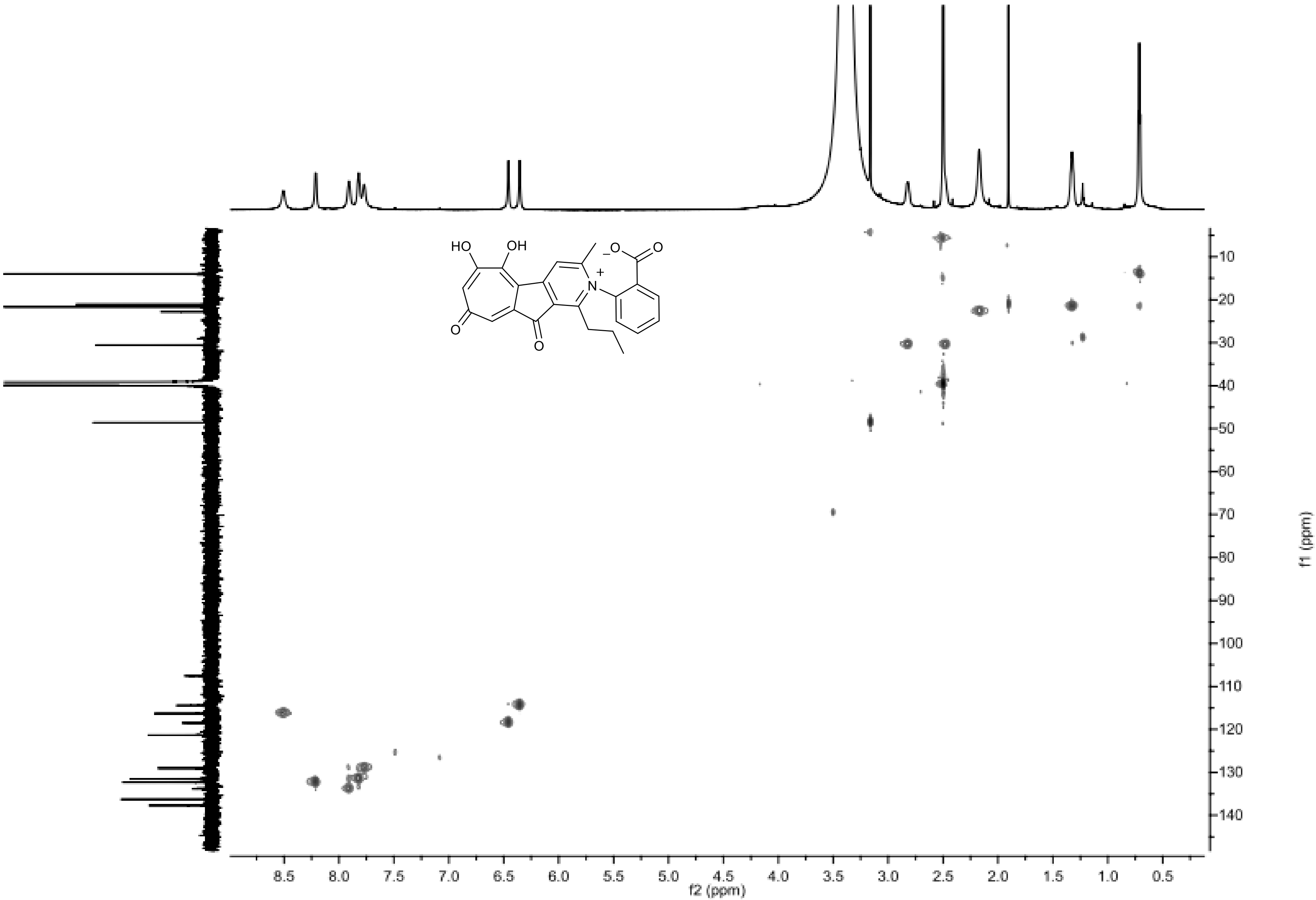

Supplementary Figure 55. HSQC NMR spectrum of compound **8** in DMSO-d_6.

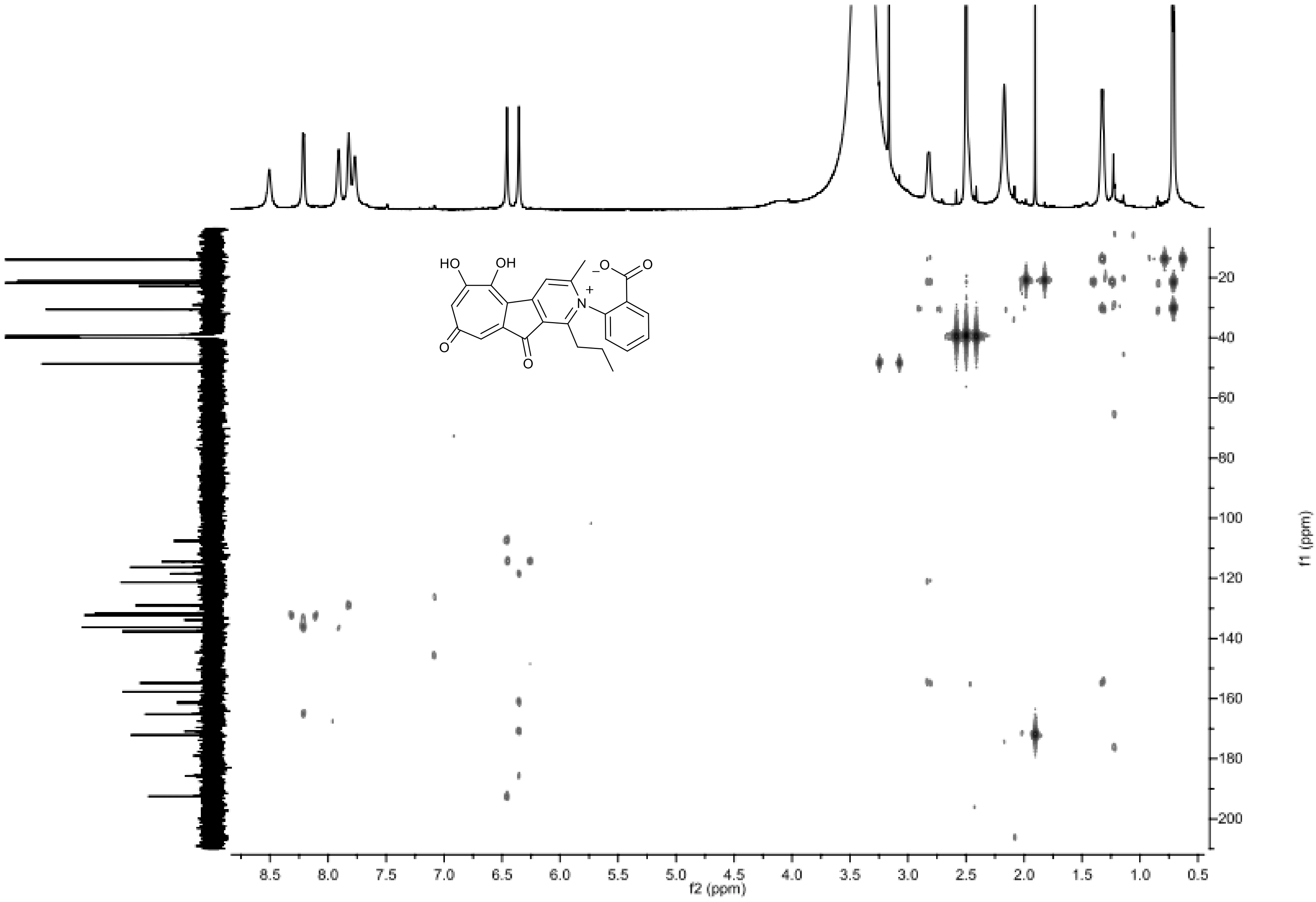

Supplementary Figure 56. HMBC NMR spectrum of compound **8** in DMSO-d_6.

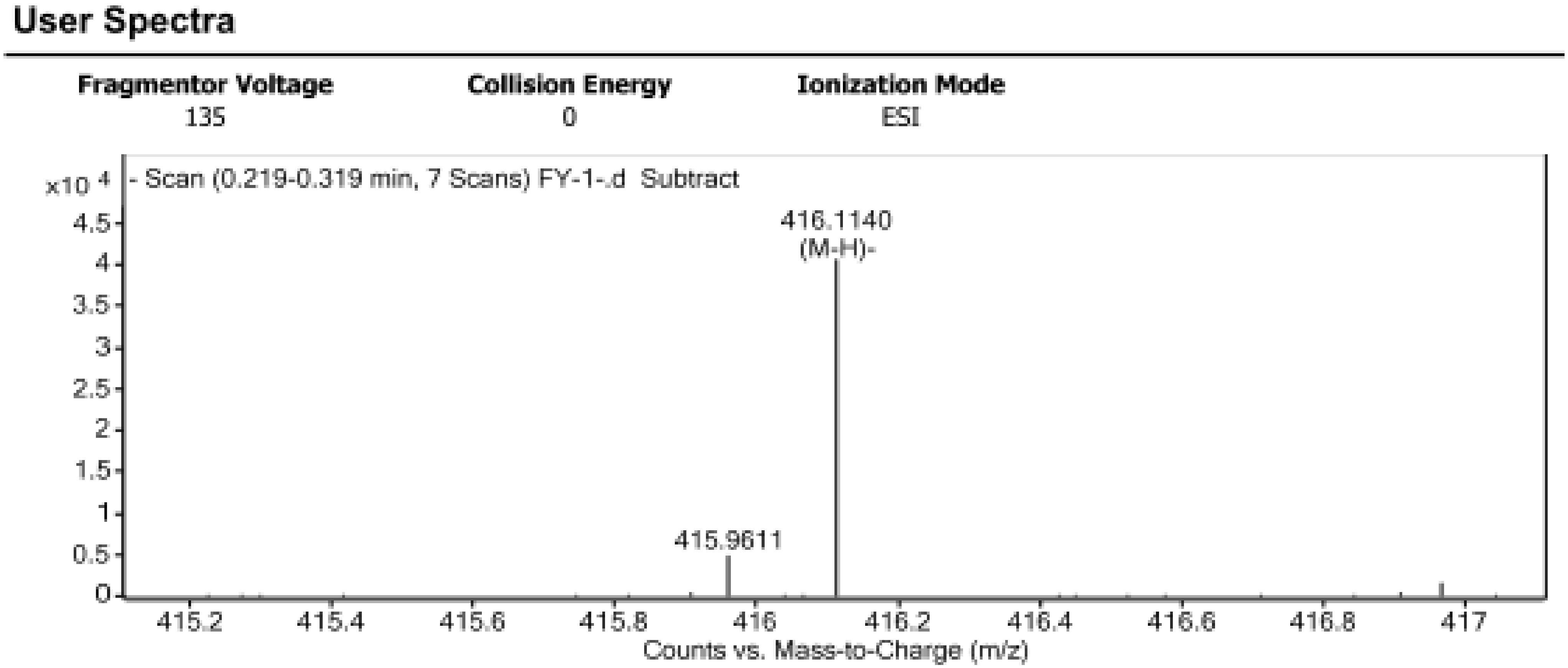

Supplementary Figure 57. HRESIMS analysis of compound **8**.

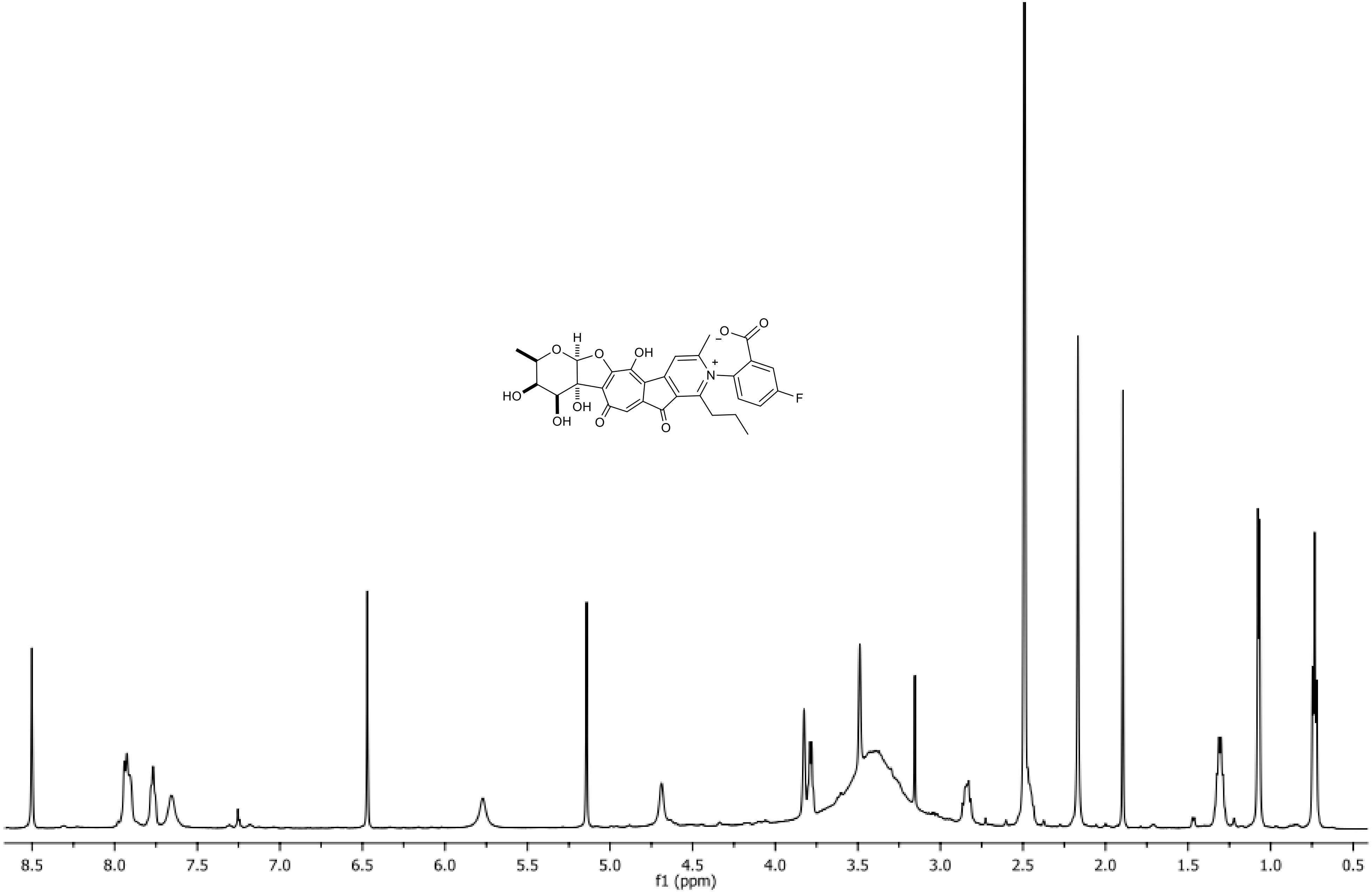

Supplementary Figure 58. ^{1}H NMR spectrum of compound **10** in DMSO-d_6.

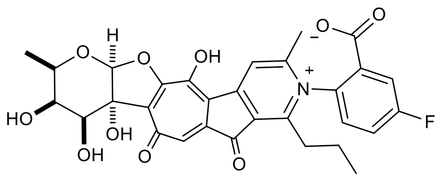

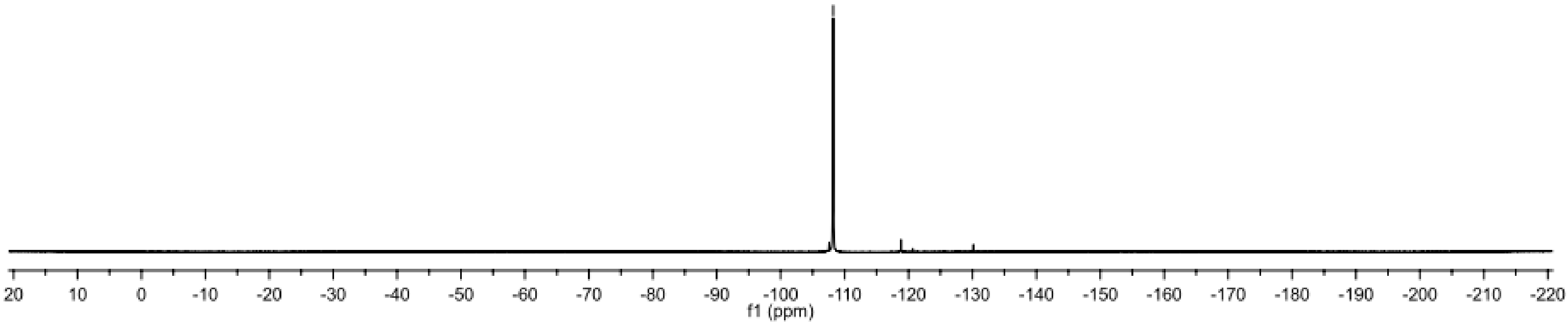

Supplementary Figure 59. ^{19}F NMR spectrum of compound **10** in DMSO-d_6.

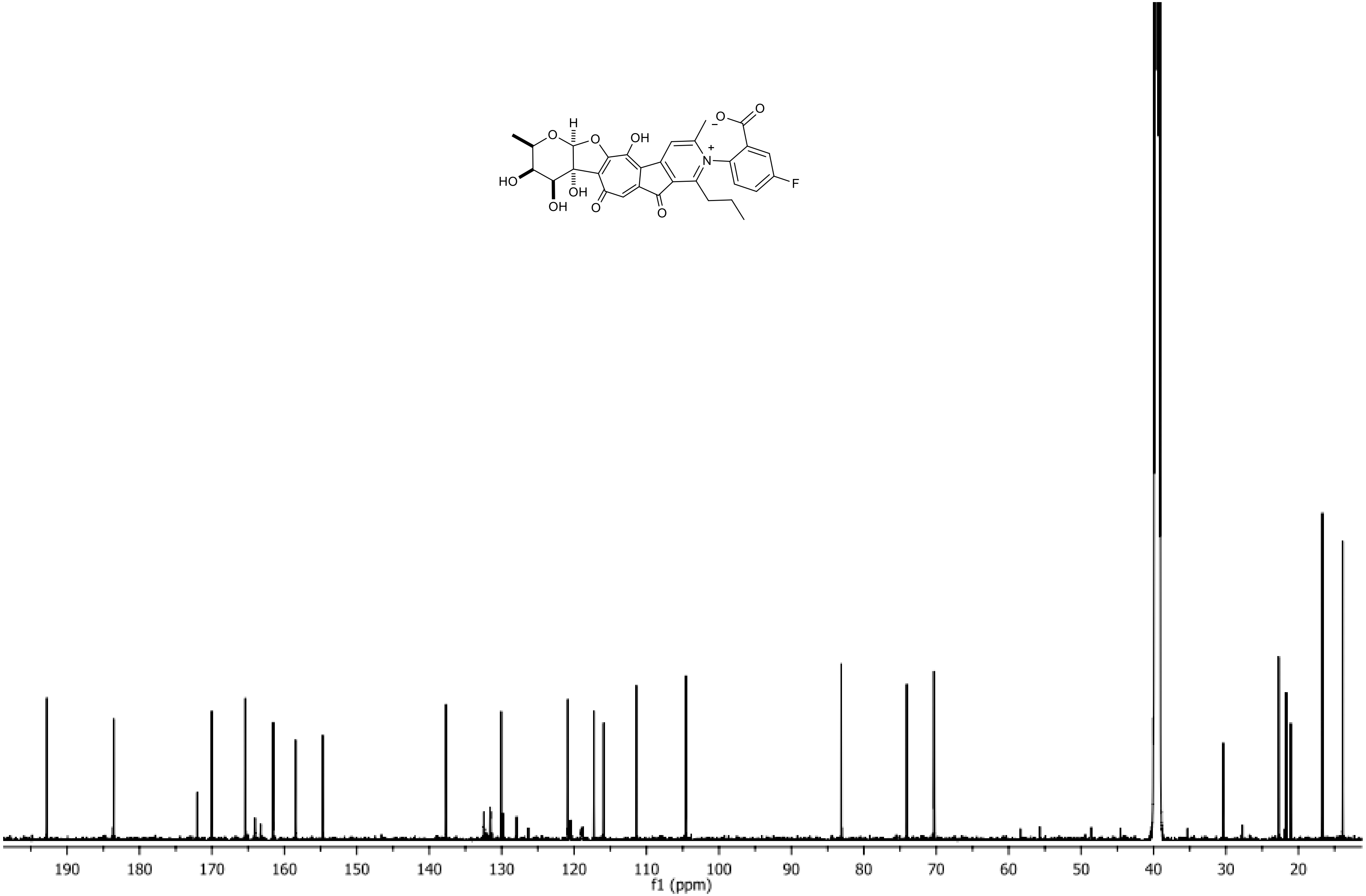

Supplementary Figure 60. ^{13}C NMR spectrum of compound **10** in DMSO-d_6.

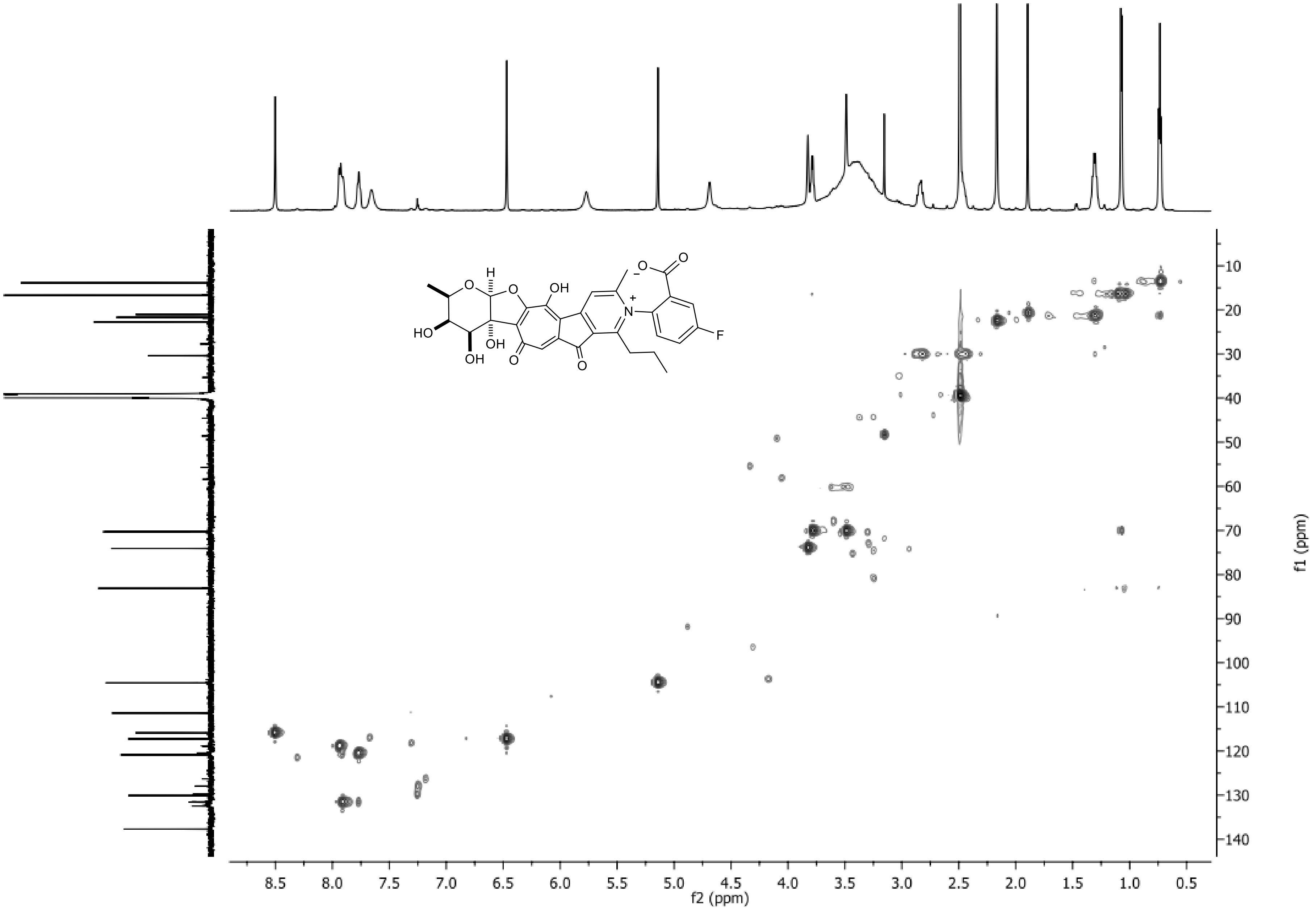

Supplementary Figure 61. HSQC NMR spectrum of compound **10** in DMSO-d_6.

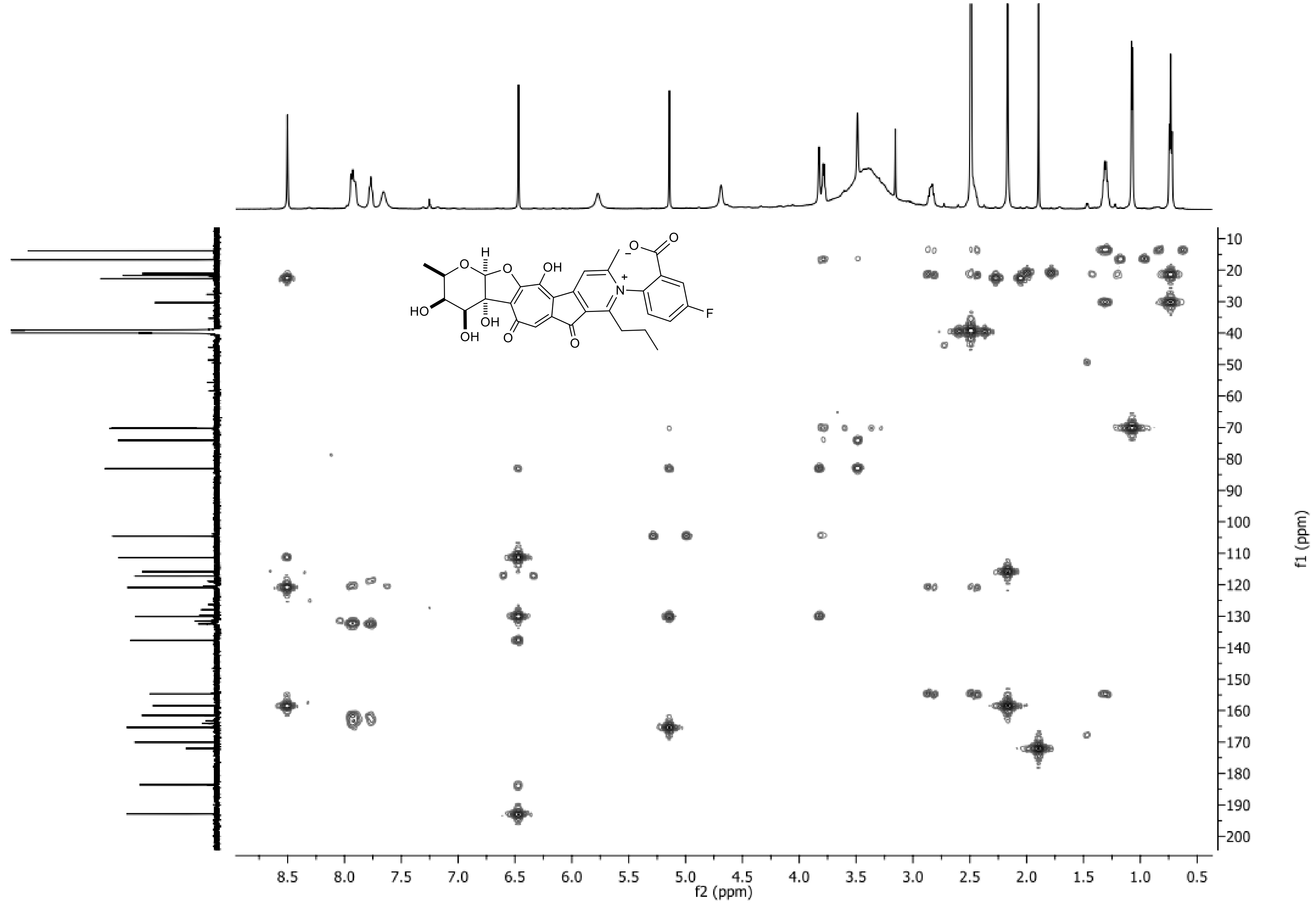

Supplementary Figure 62. HMBC NMR spectrum of compound **10** in DMSO-d_6.

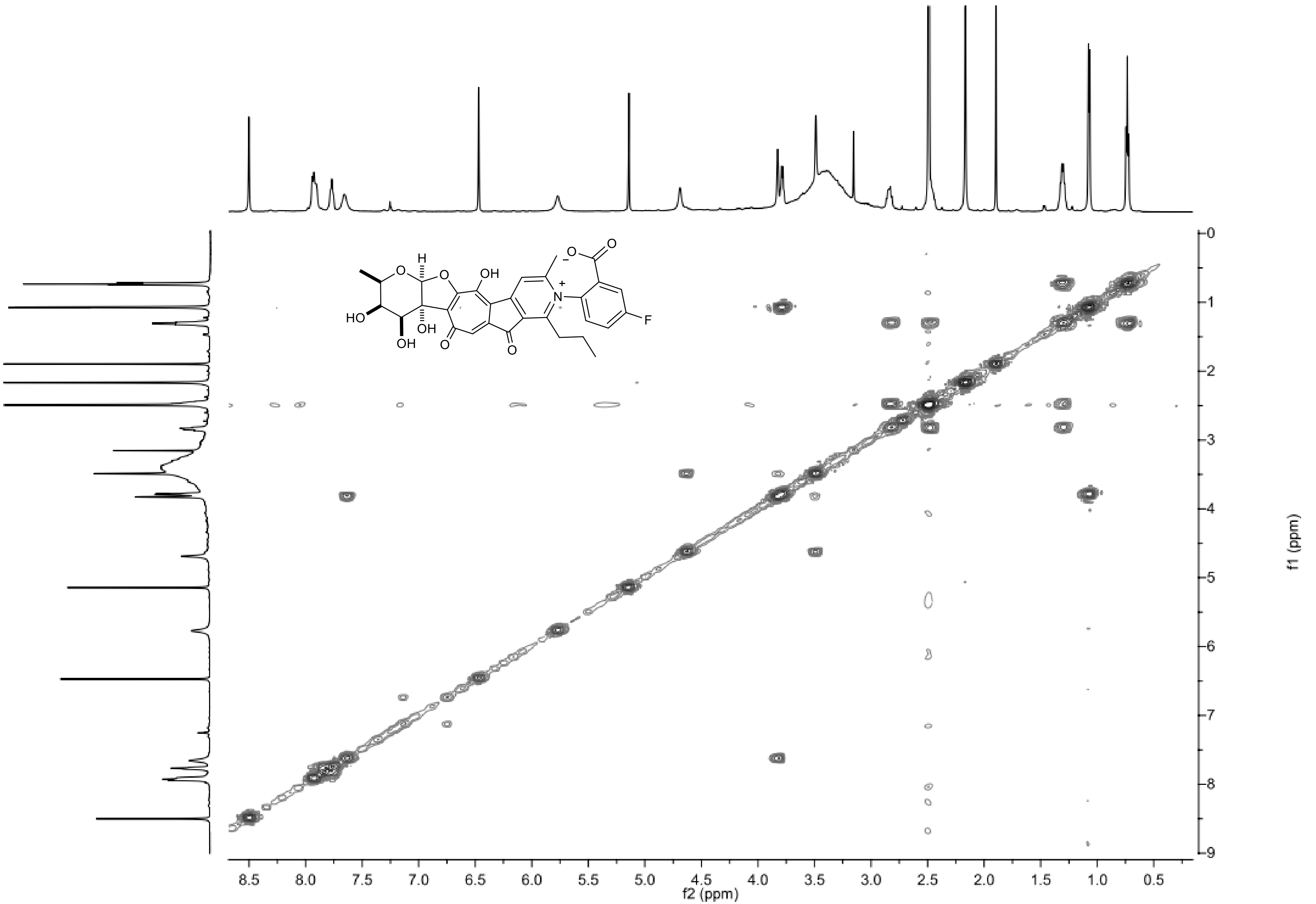

Supplementary Figure 63. ^{1}H-^{1}H COSY NMR spectrum of compound **10** in DMSO-d_6.

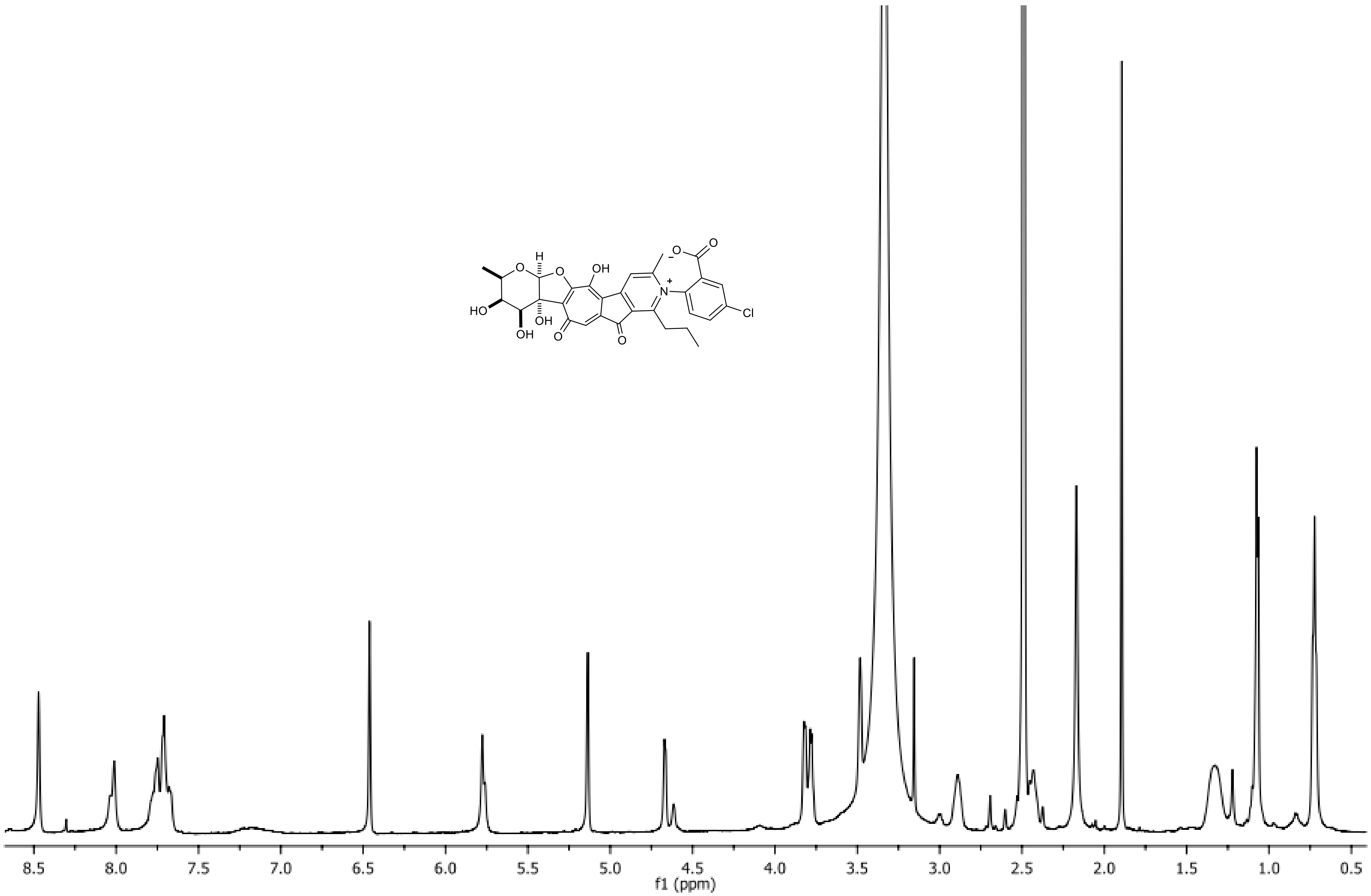

Supplementary Figure 64. ^{1}H NMR spectrum of compound **11** in DMSO-d_6.

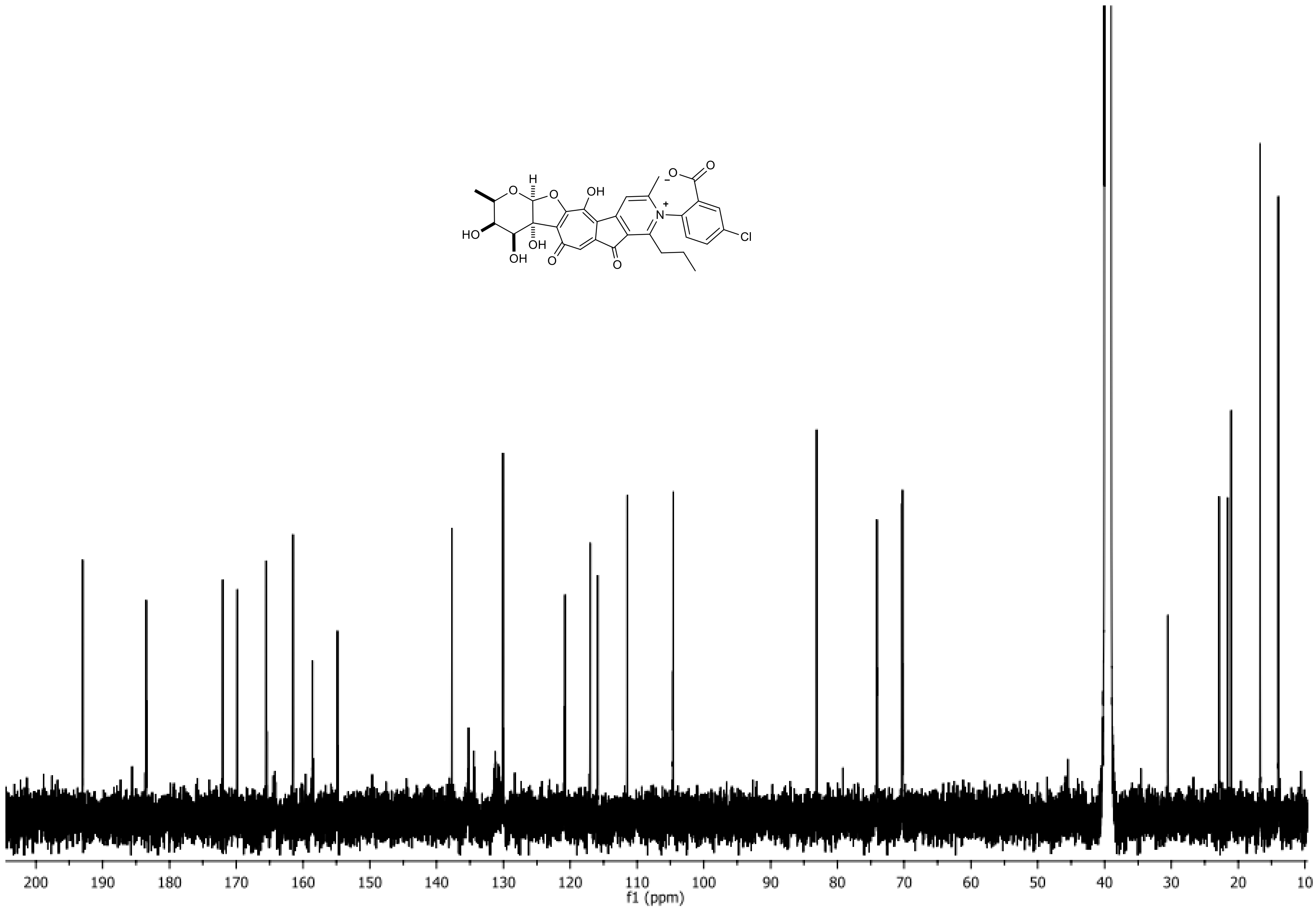

Supplementary Figure 65. ^{13}C NMR spectrum of compound **11** in DMSO-d_6.

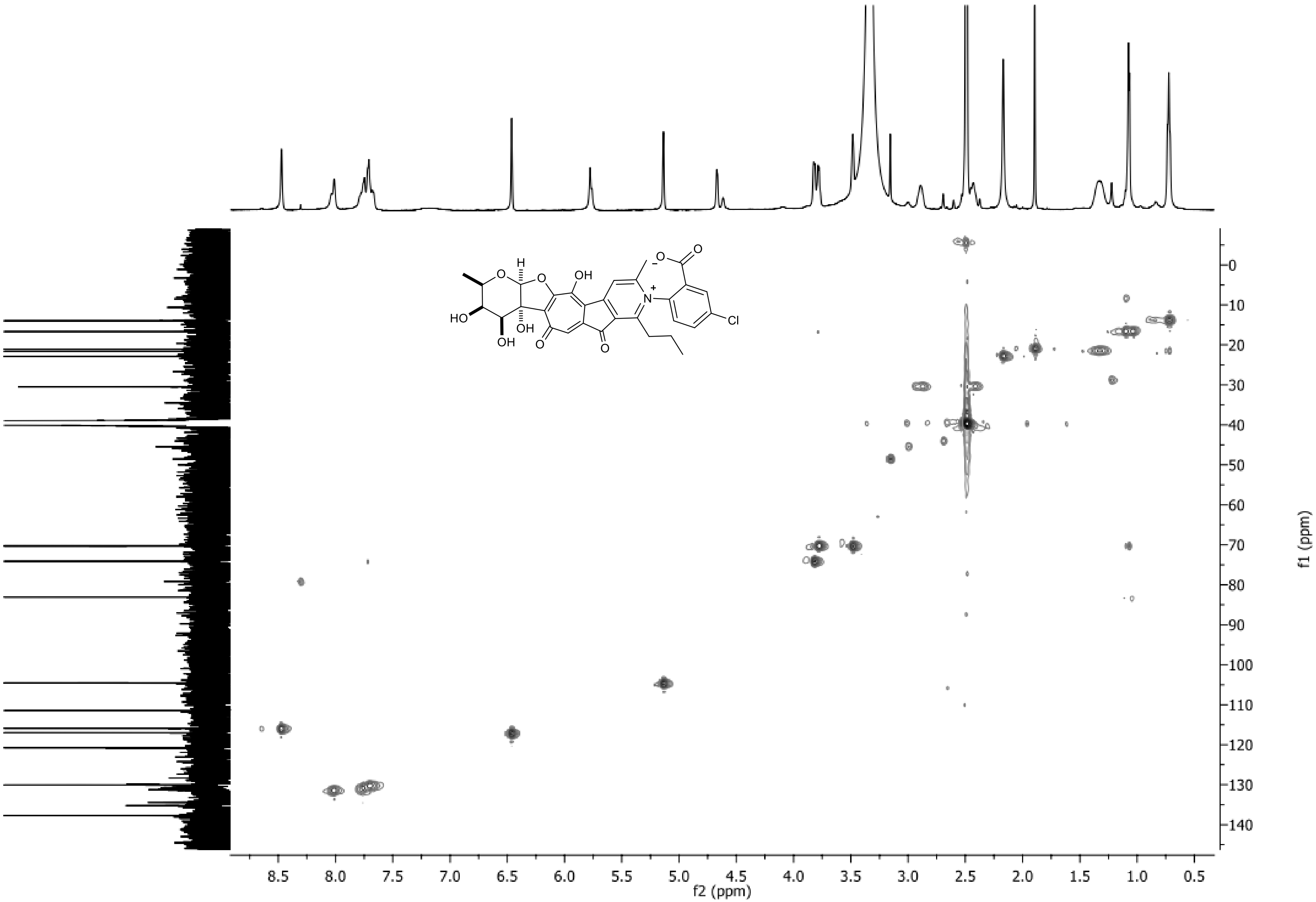

Supplementary Figure 66. HSQC NMR spectrum of compound **11** in DMSO-d_6.

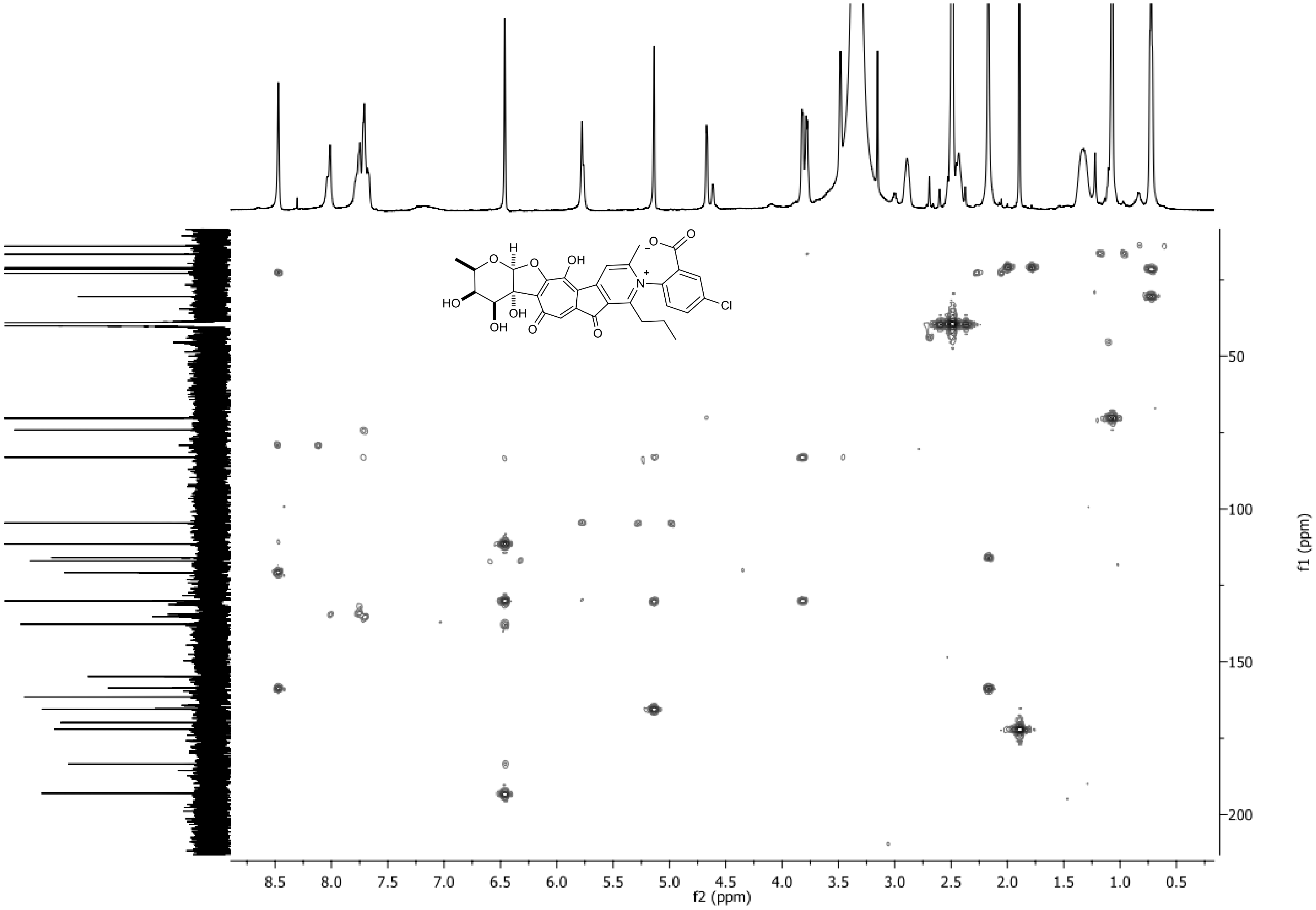

Supplementary Figure 67. HMBC NMR spectrum of compound **11** in DMSO-d_6.

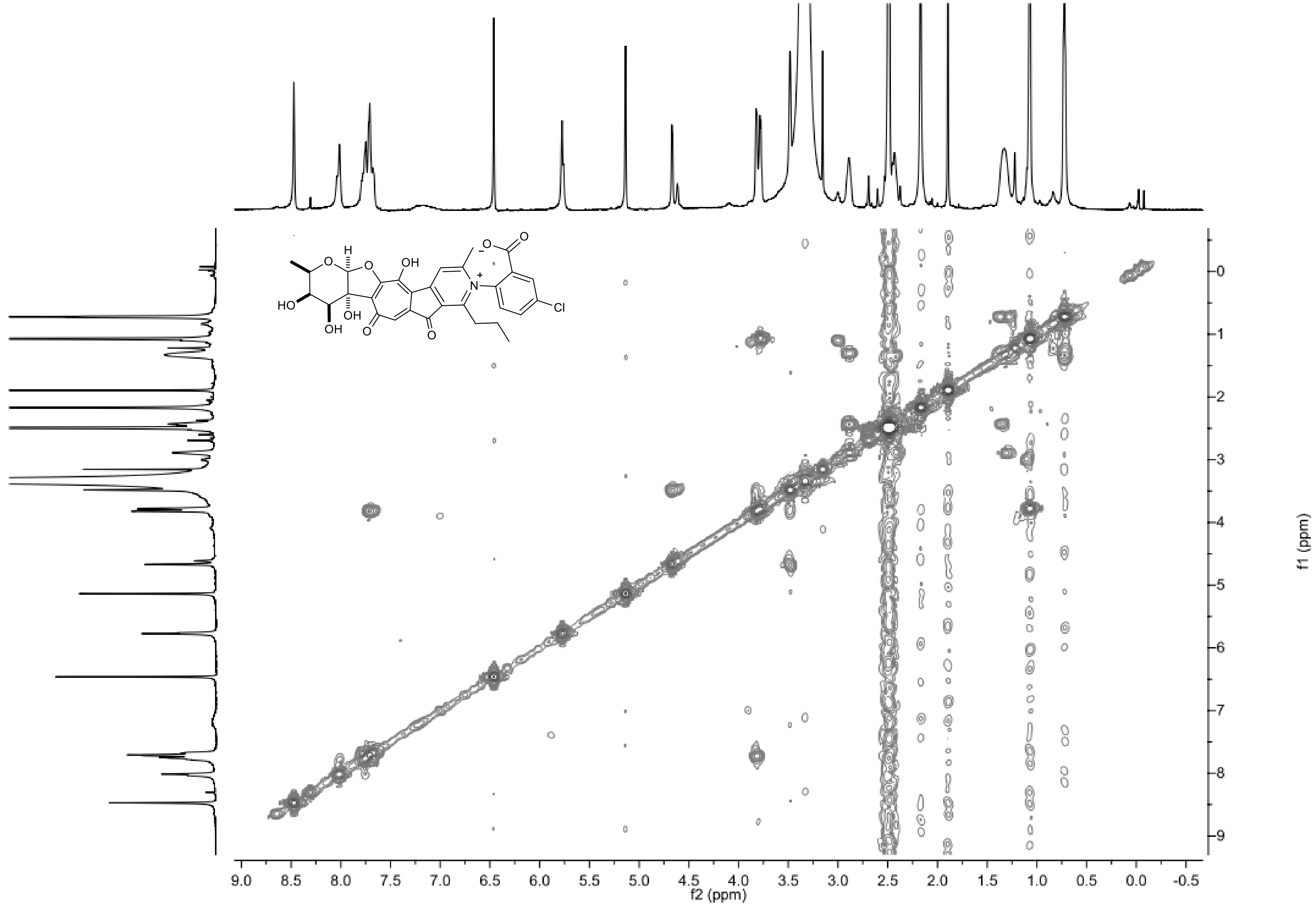

Supplementary Figure 68. ¹H-¹H COSY NMR spectrum of compound **11** in DMSO-d_6.

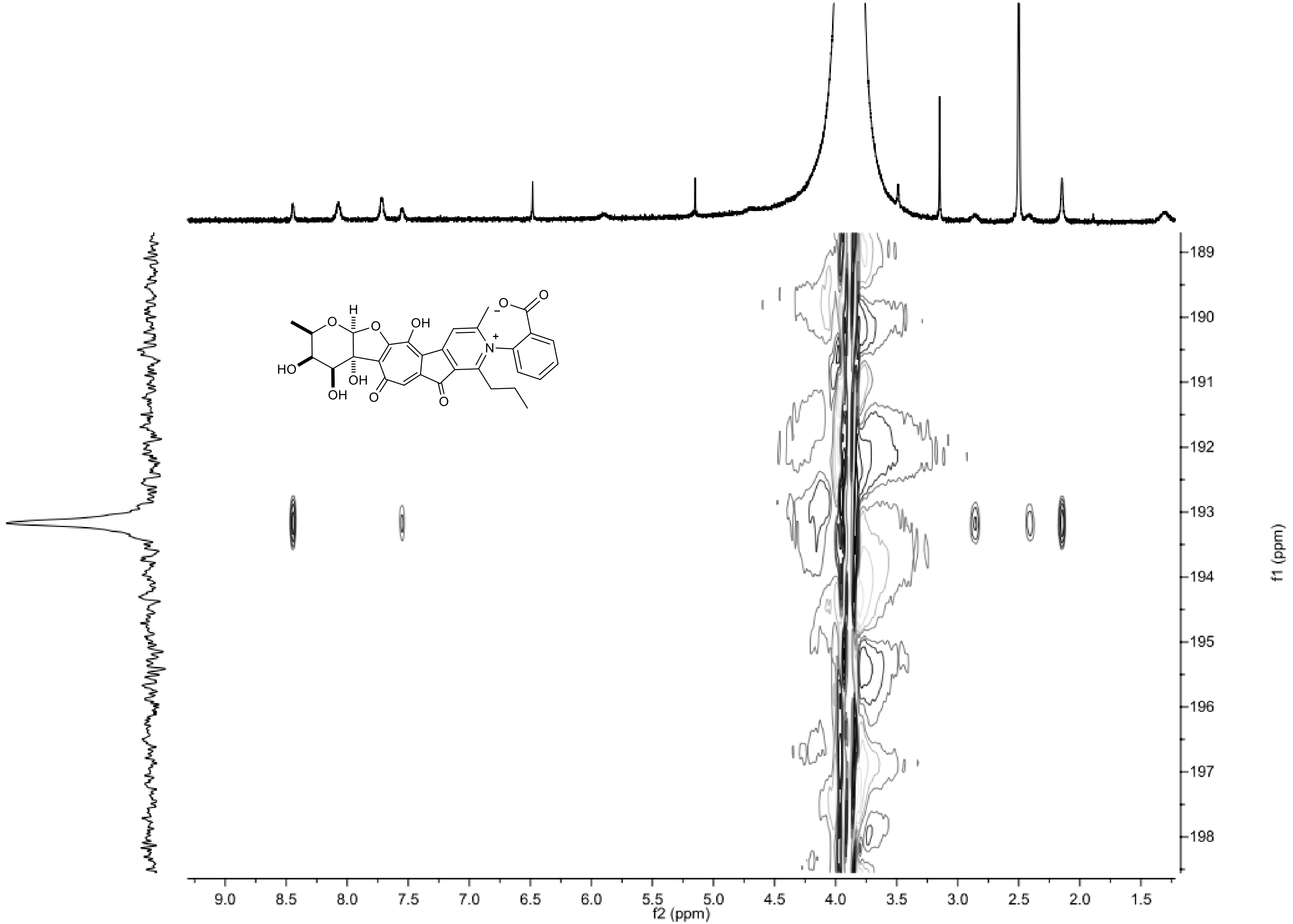

Supplementary Figure 69. Enlarged ^{1}H-^{15}N long range correlations NMR spectrum of ^{15}N-labeled **2** in DMSO-d_6

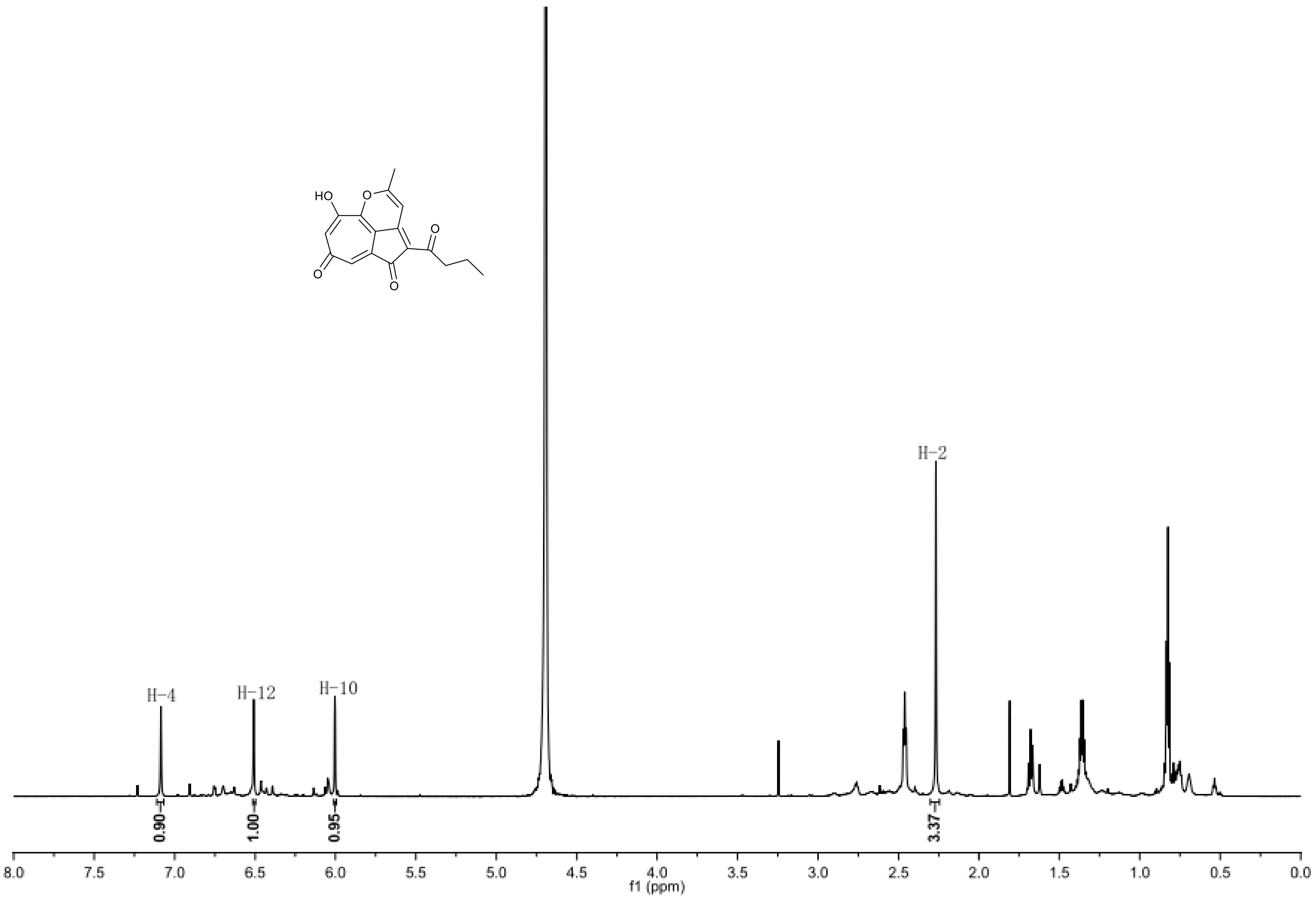

Supplementary Figure 70. ^{1}H NMR spectrum of compound **4** in D_2O after 5 minutes incubation.

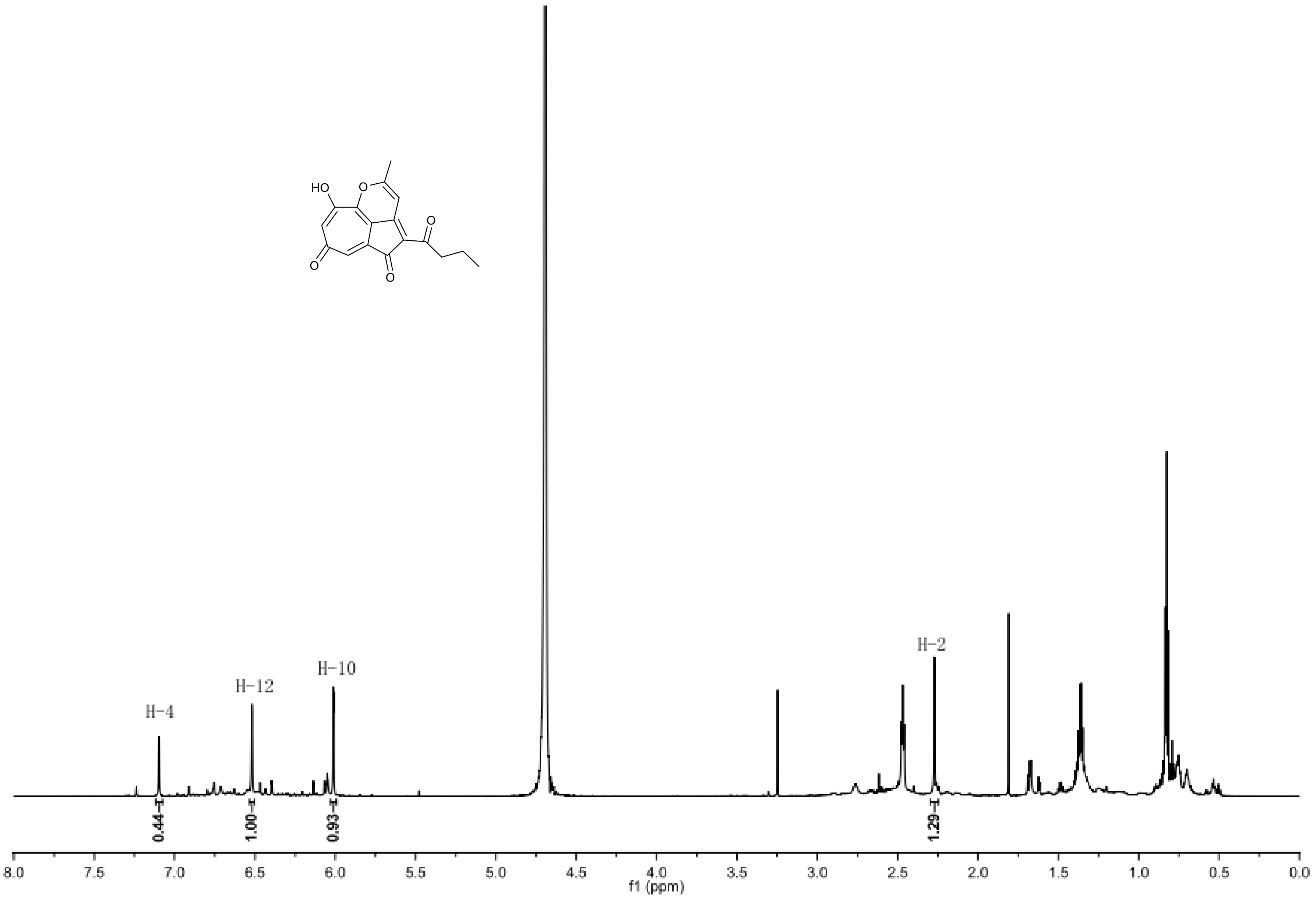

Supplementary Figure 71. ^{1}H NMR spectrum of compound **4** in D_2O after 2 hours incubation.

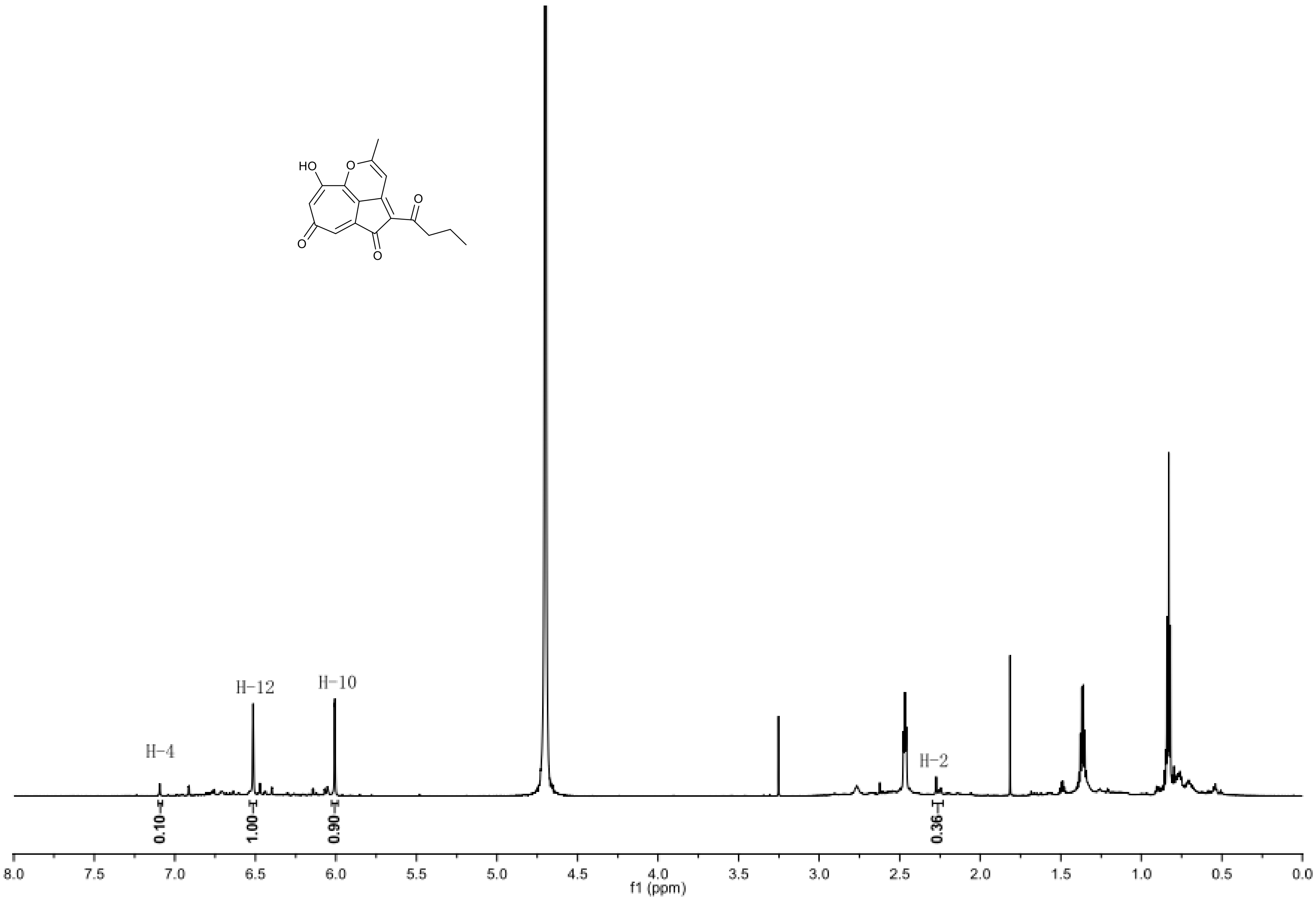

Supplementary Figure 72. ^{1}H NMR spectrum of compound **4** in D_2O after 4 hours incubation.

Supplementary Tables

Supplementary Table 1. Deduced roles of genes in *rub* gene cluster based on sequence homology.

				Closest sequence similarity		
Protein	Size (aa)	Category	Function	Protein, Origin	Positives/ Identities	Accession No.
RubS1	492	sugar	FAD-linked oxidoreductase	AknOx, *Streptomyces galilaeus*	65/52	Q0PCD7
RubN1	135	hypothetical protein	Unknown	Sgcj, *Streptomyces carzinostaticus*	56/43	4OVM
RubS2	204	sugar	dTDP-4-keto-6-deoxy-D-glucose 3,5-epimerase	StrM, *Streptomyces griseus*	63/46	P29783
RubS3	310	sugar	dTDP-glucose-4-ketoreductase	NovS, *Streptomyces niveus*	56/44	Q9L9E9
RubS4	343	sugar	dTDP-glucose 4,6 dehydratase	NovT, *Streptomyces niveus*	75/63	Q9L9E8
RubS5	289	sugar	Glucose-1-phosphate thymidylyltransferase	RfbA, *Shigella flexneri*	75/58	P37779
RubS6	278	sugar	NAD dependent epimerase/dehydratase	AzoB, *Xenophilus azovorans*	45/29	Q8KU07
RubS7	384	sugar	Glycosyltransferase	ElmGT, *Streptomyces olivaceus*	48/36	Q9F2F9
RubR	345	regulation	SARP family transcriptional regulator	DnrI, *Streptomyces peucetius*	54/39	P25047
RubN2	115	hypothetical protein	Unknown	AcrR, *Sciscionella marina*	69/51	WP_020501920
RubA	311	oxygenase	F420-dependent oxidoreductase	SsuD, *Amycolatopsis methanolica* 239	79/68	AIJ25240
RubB	518	oxygenase	Oxygenase	ZhuM, *Streptomyces* sp. R1128	67/55	AAG30200.1
RubC	389	oxygenase	naphthocyclinone hydroxylase	ActVA 5, *Streptomyces coelicolor* A3(2)	59/45	CAA41641
RubE1	319	PKS	β-ketoacylsynthase III (KSIII)	ZhuH, *Streptomyces* sp. R1128	78/61	AAG30194.1
RubE2	91	PKS	Acyl carrier protein (ACP)	ZhuG, *Streptomyces* sp. R1128	73/53	AAG30194.1
RubE3	306	PKS	Acyl transferase (AT)	ZhuC, *Streptomyces* sp. R1128	67/56	AAG30190.1
RubE4	84	PKS	Acyl carrier protein (ACP)	ZhuN, *Streptomyces* sp. R1128	82/68	AAG30201.1
RubE5	164	PKS	Bifunctional cyclase/dehydratase	ZhuJ, *Streptomyces* sp. R1128	76/69	AAG30197.1
RubE6	251	PKS	Cyclase	ZhuI, *Streptomyces* sp. R1128	88/79	AAG30196.1
RubE7	206	PKS	NADH dehydrogenase	McyH1, *Streptomyces flaveolus*	80/69	AFG19428
RubE8	409	PKS	β-ketoacylsynthase II (KSα)	ZhuB, *Streptomyces* sp. R1128	88/79	AAG30189.1
RubE9	400	PKS	Chain length factor (CLF/KSβ)	ZhuA, *Streptomyces* sp. R1128	81/71	AAG30188.1

Supplementary Table 2. NMR data of **3** and **4** in DMSO-d_6, and **5** in CD_3OD (δ in ppm, J in Hz).

No.	**3**	**4**				**5**	
	δ_H	δ_C	δ_H	COSY	HMBC	δ_C	δ_H
2	2.55 (s)	20.82	2.46 (s)		4, 3	125.42	6.89 (s)
3		169.33				163.49	
4	8.12 (s)	106.34	7.61 (s)		6, 3, 2	113.71	7.42 (s)
5		161.41				139.00	
6		122.14				184.54	
7		133.84				136.11	
8		150.12				108.40	7.03 (s)
9		nd				165.73	
10	6.50 (s)	119.54	6.48 (brs)	12	12, 8	109.53	6.48 (s)
11		nd				166.55	
12	6.69 (s)	126.62	7.07 (brs)	10	13, 10, 7, 6	111.94	
13		186.39				189.72	
14		115.07				124.01	
15		195.96				151.51	
16	3.03 (t, 7.1)	43.20	2.81 (t, 7.2)	17	18, 17, 15	39.36	3.09 (t, 7.6)
17	1.65 (m)	16.96	1.53 (m)	18, 16	18, 16, 15	25.42	1.60 (m)
18	0.93 (t, 7.3)	13.80	0.89 (t, 7.4)	17	17, 16	14.86	0.99 (t, 7.3)

[a]nd means not detected in the NMR spectra.

Supplementary Table 3. NMR data of **6** in CD_3OD and **7-8** in DMSO-d_6 (δ in ppm, J in Hz).

No.	6				7				8			
	δ_C	δ_H	COSY	HMBC	δ_C	δ_H	COSY	HMBC	δ_C	δ_H	COSY	HMBC
1	171.86											
2	132.77				51.91	2.12 (dd,13.6, 1.7), 2.80 (overlapped)	4	16, 15, 14, 4, 3	22.77	2.17 (s)		
3	159.72				207.86				157.73			
4	113.41	7.50 (s)		14,13, 6, 5, 3, 2, 1	55.29	2.36 (dd,13.0, 1.6), 2.79 (overlapped)	2	14, 6, 5, 3, 2	116.31	8.51 (s)		
5	138.25				74.25				161.61			
6	183.76				40.04	2.52 (overlapped) 3.30 (d, 16.5),		14, 12, 8, 7, 5, 4	107.54			
7	135.60				146.23				137.69			
8	108.22	6.98 (d, 1.9)	10	13, 12, 10, 7, 6	107.98	6.08 (brs)	10	12, 10, 6	170.87			
9	166.36				164.59				161.22			
10	109.49	6.45 (d, 1.9)	8	12, 9, 8	99.91	6.02 (d, 1.9)	8	9, 8	114.45	6.36 (s)		12, 11, 9, 8
11	165.42				164.60				185.74			
12	111.97				111.35				118.51	6.46 (s)		13, 10, 6
13	189.33				203.21				192.57			
14	123.85				59.65				121.32			
15	147.15				75.95				154.82			
16	35.86	3.18 (t, 7.6)	17	18, 17, 15, 14, 2	43.97	1.40 (td, 3.7, 13.0), 1.63 (td, 4.3, 13.0)	17	18, 17, 15, 14, 2,	30.57	2.82 (m), 2.49 (m)	17	18, 17, 15, 14
17	25.32	1.63 (m)	18, 16	18, 16, 15	17.40	1.26 (m), 1.52 (m)	18, 16	18, 16	21.66	1.32 (m)	18, 16	18, 16, 15
18	15.13	1.07 (t, 7.3)	17	17, 16	14.60	0.86 (t, 7.2)	17	17, 16	13.99	0.71 (t, 6.9)	17	17, 16
1'									129.02			
2'									136.30			
3'									129.03	7.77	4'	
4'									133.80	7.91	3'	
5'									131.53	7.82	6'	1'
6'									132.30	8.21	5'	2', 7'
7'									165.21			
OH-5						5.29 (brs)		14, 6, 5				
OH-11						12.89 (s)		13, 12, 11, 10				
OH-15						4.81 (s)		15, 14, 2				

Supplementary Table 4. NMR data of compounds **10** and **11** in DMSO-d_6 (δ in ppm, J in Hz).

No.	**10**					**11**			
	δ_C	δ_H	δ_F	HMBC	COSY	δ_C	δ_H	HMBC	COSY
2	22.75	2.17 (s)		4, 3		22.87	2.17 (s)	4, 3	
3	158.46					158.57			
4	115.90	8.50 (s)		14, 3, 2		115.90	8.47 (s)	14, 3, 2	
5	161.53					161.53			
6	111.41					111.45			
7	137.69					137.69			
8	170.02					169.83			
9	165.39					165.53			
10	130.07					130.08			
11	183.56					183.49			
12	117.27	6.47 (s)		13, 11, 10, 7, 6		117.00	6.46 (s)	13, 11, 10, 7, 6	
13	192.79					192.98			
14	120.88					120.82			
15	154.70					154.83			
16	30.38	2.48 (m)		18, 17, 14	H-17	30.51	2.48 (m)		H-17
		2.84 (m)					2.89 (m)		
17	21.67	1.30 (m)		18, 16	H-18, H-16	21.59	1.33 (m)		H-18, H-16
18	13.88	0.73 (t, 7.1)		17, 16	H-17	13.97	0.72 (t, 6.1)	17, 16	H-17
19	104.55	5.14 (s)		20, 10, 9		104.55	5.13 (s)	20, 10, 9	
20	83.11					83.09			
21	74.07	3.82 (brs)		20, 10	H-22, OH-21	74.10	3.82 (brs)	20, 10	H-22, OH-21
22	70.31	3.49 (brs)		24, 21, 20	H-23, OH-22, H-21	70.35	3.48 (brs)		OH-22, H-21
23	70.26	3.78 (m)		24, 22, 21, 19	H-24, H-22	70.23	3.78 (m)		H-24, H-22
24	16.69	1.07 (d, 6.2)		23, 22	H-23	16.69	1.07 (d, 5.9)	23, 22	H-23
1’	129.79					128.31			
2’	132.45					134.44			
3’	131.58	7.91 (m)		5’, 1’	H-4’	129.97	7.71 (m)	5’	H-4’
4’	120.44	7.77 (m)		6’, 2’	H-3’	131.23	8.01 (m)	2’	H-3’
5’	162.39		-108.22			135.19			
6’	118.82	7.94 (m)		4’, 2’		130.75	7.75 (m)	4’, 2’	
7’	164.06					165.43			
OH-20		5.77 (s)					5.78 (s)	19	
OH-21		7.66 (brs)			H-21		7.68 (brs)	21, 20	H-21
OH-22		4.69 (s)			H-22		4.67 (s)	23	H-22

Supplementary Table 5. Strains and plasmids used and generated in this study.

Strains/Plasmid	Purpose	Sources
Strains		
E. coli		
DH10B	Host strain for cloning	Invitrogen
BW25113/pIJ790	Host strain for PCR targeting	Ref. 10
ET12567/pUZ8002	Donor strain for conjugation	Ref. 11
XL1-blue MR	Host strain for genomic library	Agilent Technologies
DH5α/BT340	Host strain for in-frame deletion	Ref. 12
Streptomyces		
Streptomyces sp. KIB-H033	Rubrolones wild type producing strain	This study
Streptomyces albus J1074	Host strain for heterologous expression	Ref. 13
S. albus 9B10	*Streptomyces albus* J1074 integrated with plasmid 9B10 which contains *rub* biosynthetic gene cluster	This study
S. albus 9B10-1	*orf1-orf7* inactivation mutant of *S. albus* 9B10	This study
S. albus 9B10-2	*orf30-orf37* inactivation mutant of *S. albus* 9B10	This study
S. albus 9B10-Δ*S1*	*rubS1* inactivation mutant of *S. albus* 9B10	This study
S. albus 9B10-Δ*S2*	*rubS2* inactivation mutant of *S. albus* 9B10	This study
S. albus 9B10-Δ*S3*	*rubS3* inactivation mutant of *S. albus* 9B10	This study
S. albus 9B10-Δ*S7*	*rubS7* inactivation mutant of *S. albus* 9B10	This study
S. albus 9B10-Δ*A*	*rubA* inactivation mutant of *S. albus* 9B10	This study
S. albus 9B10-Δ*B*	*rubB* inactivation mutant of *S. albus* 9B10	This study
S. albus 9B10-Δ*C*	*rubC* inactivation mutant of *S. albus* 9B10	This study
S. albus 9B10-Δ*E7*	*rubE7* inactivation mutant of *S. albus* 9B10	This study
S. albus 9B10-Δ*E9*	*rubE9* inactivation mutant of *S. albus* 9B10	This study
Plasmids		
pJTU2554	Apr^r, Cosmid vector for genomic library construction	Ref. 10
pJTU6722	Ery^r, Vector for PCR targeting	Constructed by Prof. Meifeng Tao
p9B10	Apr^r, Cosmid which contains *rub* biosynthetic gene cluster	This study
p9B10-1	Apr^r, gene inactivation clone used for *orf1-orf7* mutant	This study
p9B10-2	Apr^r, gene inactivation clone used for *orf30-orf37* mutant	This study
p9B10-Δ*S1*	Apr^r, gene inactivation clone used for *rubS1* mutant	This study
p9B10-Δ*S2*	Apr^r, gene inactivation clone used for *rubS2* mutant	This study
p9B10-Δ*S3*	Apr^r, gene inactivation clone used for *rubS3* mutant	This study
p9B10-Δ*S7*	Apr^r, gene inactivation clone used for *rubS7* mutant	This study
p9B10-Δ*A*	Apr^r, gene inactivation clone used for *rubA* mutant	This study
p9B10-Δ*B*	Apr^r, gene inactivation clone used for *rubB* mutant	This study
p9B10-Δ*C*	Apr^r, gene inactivation clone used for *rubC* mutant	This study
p9B10-Δ*E7*	Apr^r, gene inactivation clone used for *rubE7* mutant	This study
p9B10-Δ*E9*	Apr^r, gene inactivation clone used for *rubE9* mutant	This study

Supplementary Table 6. Primers used for constructing and confirming the mutants.

Primers	Targeted genes	Sequences
For PCR targeting		
Tar-H2517-F	*orf1-orf7*	5'-CTCCGACGCCGTCGGTGAAATCTTTTCAGGAGGAAGTCCattccggggatccgtcgacc-3'
Tar-H2511-R		5'-GTGCACCCGCAACAGCGAGTACTGGATCCTGCGCACCGGtgtaggctggagctgcttc-3'
Tar-H2518-F	*rubS1*	5'-ACCGTCCGGCCCGGCGACCCGCGCTACGACGACCTGGCGattccggggatccgtcgacc-3'
Tar-H2518-R		5'-CCGCGCTCTCGATCTCCGTGCGGGCGGCATCGGTGCTCGtgtaggctggagctgcttc-3'
Tar-H2549-F-	*orf30-orf37*	5'-CCGGCACCCTCGCGGCGGGCATCTTCGCGGTCGGCACCCattccggggatccgtcgacc-3'
Tar-H2542-R		5'-TTCGGGTTCCGACCGAGGCAGGACAGCGGGCCGGTCCGCtgtaggctggagctgcttc-3'
Tar-H2541-F	*rubE9*	5'-CCGATGAGGCCGCCCGCAGTCGTCACCGGGATCGGGGTGattccggggatccgtcgacc-3'
Tar-H2541-R		5'-GGGTCCCGCTTCGGGTTCCGACCGAGGGCGGCGGGGTCAtgtaggctggagctgcttc-3'
Tar-H2539-F	*rubE7*	5'-GCAACGACGGCGGCACGGCTCCTGGCGCTGGACGCGGCGattccggggatccgtcgacc-3'
Tar-H2539-R		5'-AGCGCAAACCCGTGCACGAGGTCGCCCAGGCGGAGGTCAtgtaggctggagctgcttc-3'
Tar-H2528-F	*rubA*	5'-CTGCGCAAACCGATCTCTGAAATGGGGTGCTGGGCAATGattccggggatccgtcgacc-3'
Tar-H2528-R		5'-GCCCGGGCCGCCGGGTCCGGGCACCGGGCCCGGCGGTCAtgtaggctggagctgcttc-3'
Tar-H2530-F	*rubB*	5'-ACCCGCGTCGGCTGCCGTCGCCGATGGGAGTGGGAGATGattccggggatccgtcgacc-3'
Tar-H2530-R		5'-GGTCACCACGTCGACGCGTCCGGCCCAGCCGGCGGCCGTtgtaggctggagctgcttc-3'
Tar-H2531-F	*rubC*	5'-CCGAAGGAGACTGATGTGACGCACCCAGTAGCCGCCGGGattccggggatccgtcgacc-3'
Tar-H2531-R		5'-TGCCGGGGACGGCAGGGCGGCGGCCGGGTGCACGGGTCAtgtaggctggagctgcttc-3'
Tar-H2520-F	*rubS2*	5'-ACAACGTGCGGCCGCGCCTCGCCCCCGGAGCCGAAGACCattccggggatccgtcgacc-3'
Tar-H2520-R		5'-GCGGCCGGCACGGCGGACGGGCGCCGGGGGCGGTGTGCGtgtaggctggagctgcttc-3'
Tar-H2521-F	*rubS3*	5'-GCCACAGGGCGAGGTCCGCGTCGGAGGGGCCGCTGTCGGattccggggatccgtcgacc-3'
Tar-H2521-R		5'-GGCAGTCGGCGGTGCGGTCACCGCCCCGGCCCGGGGGGCtgtaggctggagctgcttc-3'
Tar-H2525-F	*rubS7*	5'-ATTGCCGACTCCTCGACAAAGGGCGTATGCGCAGCCATGattccggggatccgtcgacc-3'
Tar-H2525-R		5'-CTCCGCGGTCAGTTGTGTCCTGTCGGCGGCGACACCCAGtgtaggctggagctgcttc-3'
For gene validation		
H2518-check-F	*rubS1*	5'-GTGAGTGTTCCTTGAGCAA-3'
H2518-check-R		5'-GAACTTGCGGATGTCCTC-3'
H2541-check-F	*rubE9*	5'-CAACCTGCACGAACCGGATC-3'
H2541-check-R		5'-AGCACATGACGAAGGGCCTG-3'
H2539-check-F	*rubE7*	5'-TGCGCGAGATGGTCGAGGAGTA-3'
H2539-check-R		5'-CGGAAGTCAGCAGGTCCACGAAT-3'
H2528-check-F	*rubA*	5'-CATGGTCGCATTCTGTCCTCTCAT-3'
H2528-check-R		5'-GCGTTCATCGAGCACTGGAAGT-3'
H2530-check-F	*rubB*	5'-CTGCCTCAGCCATTGCTCAAGA-3'
H2530-check-R		5'-GGTGCGTCACATCAGTCTCCTTC-3'
H2531-check-F	*rubC*	5'-AGCGTGACCGAAGCGATGCA-3'
H2531-check-R		5'-AACACCTCAGCGGCGTCGAT-3'
H2520-check-F	*rubS2*	5'-GGACATCCGCAAGTTCAT-3'
H2520-check-R		5'-CTGCACTACGTCAACACT-3'
H2521-check-F	*rubS3*	5'-GCCTGAAGCTGATCCTGT-3'
H2521-check-R		5'-GATCGACGACACGAGGAT-3'
H2525-check-F	*rubS7*	5'-GCGGCTACTTGACGATGTCCTC-3'
H2525-check-R		5'-GCTACCACGAGTACCTGATGTTCT-3'

Supplementary References

1. Dan, C. *et al.* Cimicifugadine from *Cimicifuga foetida*, a new class of triterpene alklaoids with novel reactivity. *Org. Lett*. **9**, 1813-1816 (2007).
2. Baghdikian, B. *et al.* Two new pyridine monoterpene alkaloids by chemical conversion of a commercial extract of *Harpagophytum procumbens*. *J. Nat. Prod*. **62**, 211-213 (1999).
3. Oshimi, S. *et al*. Cassiarins C-E, antiplasmodial alkaloids from the flowers of *Cassia siamea*. *J. Nat. Prod*. **72**, 1899-1901 (2009).
4. Okatani, T., Koyama, J., Tagahara, K. & Suzuta, Y. Synthesis of sesquiterpene alkaloids, guaipyridine: epiguaipyridine and related compounds. *Heterocycles* **26**, 595-597 (1987).
5. Edwards, O. E. & Elmore, N. F. Fabianine. *Can. J. Chem.* **40**, 256-264 (1962).
6. Tasdemir, D. *et al.* Deoxyamphimedine, a new pyridoacridine alkaloid from two tropical *Xestospongia* sponges. *J. Org. Chem.* **66**, 3246-3248 (2001).
7. Rakhmatullaev, T. U. & Yunusov, S. Y. Alkaloids of *Gentiana olivieri. Chem. Nat. Compd*. **9**, 56-58 (1973).
8. Cash, B. M., Prevost, N., Wagner, F. F. & Comins, D. L. Studies toward the total synthesis of dihydrolycolucine, preparation of AB and CEF ring fragments. *J. Org. Chem*. **79**, 5740-5745 (2014).
9. Jayasinghe, L., Jayasooriya, C. P., Hara, N. & Fujimoto, Y. A pyridine ring-containing ecdysteroid from *Diploclisia glaucescens*. *Tetrahedron Lett*. **44**, 8769-8771 (2003).
10. Li, L. *et al.* The mildiomycin biosynthesis: initial steps for sequential generation of 5-hydroxymethylcytidine 5'-monophosphate and 5-hydroxymethylcytosine in *Streptoverticillium rimofaciens* ZJU5119. *ChemBioChem* **9**, 1286-1294 (2008).
11. Paget, M. S., Chamberlin, L., Atrih, A., Foster, S. J. & Buttner, M. J. Evidence that the extracytoplasmic function sigma factor sigmaE is required for normal cell wall structure in *Streptomyces coelicolor* A3(2). *J. Bacteriol.* **181**, 204-211 (1999).
12. Datsenko, K. A. & Wanner, B. L. One-step inactivation of chromosomal genes in *Escherichia coli* K-12 using PCR products. *Proc. Natl. Acad. Sci. U.S.A.* **97**, 6640-6645 (2000).
13. Chater, K. F. & Wilde, L. C. *Streptomyces albus* G mutants defective in the SalGI restriction-modification system. *J. Gen. Microbiol*. **116**, 323-334 (1980).
